# Identifying Differing Intracellular Cargo Release Mechanisms by Monitoring *In Vitro* Drug Delivery from MOFs in Real Time

**DOI:** 10.1016/j.xcrp.2020.100254

**Published:** 2020-11-18

**Authors:** Panagiota Markopoulou, Nikolaos Panagiotou, Aurelia Li, Rocio Bueno-Perez, David Madden, Sarah Buchanan, David Fairen-Jimenez, Paul G. Shiels, Ross S. Forgan

**Affiliations:** 1 Joseph Black Building, College of Science and Engineering, School of Chemistry, University of Glasgow, Glasgow G12 8QQ, UK; 2 Adsorption & Advanced Materials Laboratory, Department of Chemical Engineering & Biotechnology, University of Cambridge, Cambridge CB3 0AS, UK; 3 Wolfson Wohl Cancer Research Centre, College of Medical, Veterinary, & Life Sciences, Institute of Cancer Sciences, University of Glasgow, Glasgow G61 1QH, UK

**Keywords:** metal-organic frameworks, nanomedicine, drug delivery, anticancer, real-time cell analysis, *in vitro*, doxorubicin

## Abstract

Metal-organic frameworks (MOFs) have been proposed as biocompatible candidates for the targeted intracellular delivery of chemotherapeutic payloads, but the site of drug loading and subsequent effect on intracellular release is often overlooked. Here, we analyze doxorubicin delivery to cancer cells by MIL-101(Cr) and UiO-66 in real time. Having experimentally and computationally verified that doxorubicin is pore loaded in MIL-101(Cr) and surface loaded on UiO-66, different time-dependent cytotoxicity profiles are observed by real-time cell analysis and confocal microscopy. The attenuated release of aggregated doxorubicin from the surface of Dox@UiO-66 results in a 12 to 16 h induction of cytotoxicity, while rapid release of pore-dispersed doxorubicin from Dox@MIL-101(Cr) leads to significantly higher intranuclear localization and rapid cell death. In verifying real-time cell analysis as a versatile tool to assess biocompatibility and drug delivery, we show that the localization of drugs in (or on) MOF nanoparticles controls delivery profiles and is key to understanding *in vitro* modes of action.

## Highlights

Comprehensive analysis confirms *in vitro* biocompatibility of UiO-66 and MIL-101(Cr)
Real-time observation of doxorubicin delivery from MOFs and subsequent cytotoxicity
Location of drug within MOF nanoparticles greatly affects release mechanisms
Tunable anticancer cytotoxicity profiles are possible by modifying drug location

## Graphical Abstract

**Figure:**
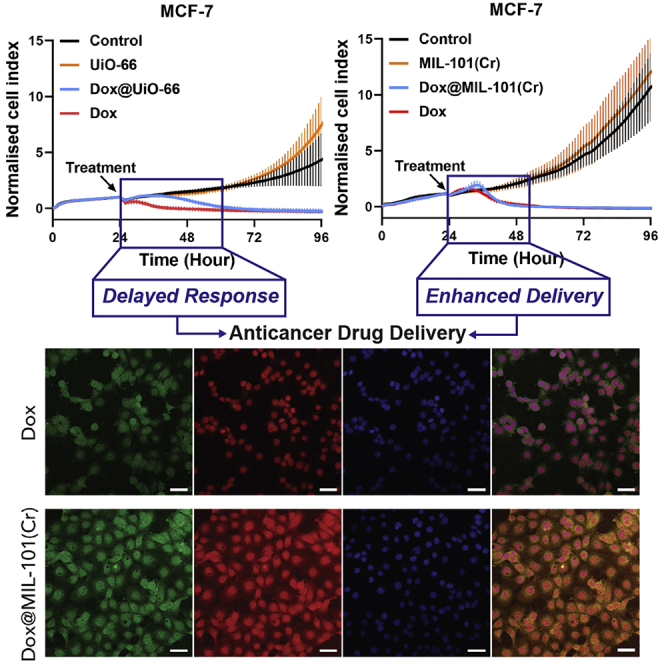

## Teaser

Markopoulou et al. demonstrate that real-time cell analysis can be used to assess biocompatibility and drug delivery from MOFs. Different time-dependent cytotoxicity profiles occur when drugs are pore loaded versus surface loaded, making tunable drug release possible.

## Introduction

Metal-organic frameworks (MOFs), coordination networks of metal ions or clusters linked by organic ligands into potentially porous materials, are being investigated increasingly as potential drug delivery systems (DDSs).^1^, ^2^, ^3^, ^4^, ^5^, ^6^ The ability to control particle size,^7^, ^8^, ^9^ surface chemistry,^10^ and internal porosity^11^^,^^12^ has led to increasingly complex MOF-based materials. These have been designed to target specific cells^13^ and organelles,^14^ transport large specialized cargo such as oligonucleotides and proteins,^15^, ^16^, ^17^, ^18^, ^19^ release these in response to specific stimuli,^20^^,^^21^ and combine drug delivery with other techniques such as imaging^22^, ^23^, ^24^, ^25^, ^26^ or photodynamic therapy.^27^^,^^28^ Despite this diversification of material, the process of postsynthetic drug loading itself is often undercharacterized; cargo is often simply assumed to penetrate the porosity of the MOF despite potential competition from loading solvents. Additionally, binding cargo to the external surface of particles is already an established strategy for the delivery of larger molecules.^15^ Typically, the cytotoxicity and efficacy of drug delivery are monitored *in vitro* by endpoint assays, in which a parameter, typically cell proliferation, is measured after incubation of the DDS with cells for a particular time, therefore only capturing a snapshot of data at a single time point. While these assays can be carried out over differing timescales, this consumes time and additional materials, and by necessity each time point is collected on a different cell population. We show here that real-time cell analysis (RTCA) can be applied not only to assess the real-time biocompatibility of MOFs^29^^,^^30^ but also to monitor the cellular response to drug delivery. The potential of the technique is demonstrated by discriminating between differing mechanisms of intracellular doxorubicin (Dox) release from two benchmark MOF DDSs, and in doing so, uncovering a potential candidate for controlled, enhanced Dox delivery over a number of hours.

Dox is widely used in studies involving novel DDSs, as it has strong absorption (ε ∼10^4^ Lmol^−1^cm^−1^ at λ_max_ = 480 nm) and emission (λ_em_ ∼600 nm, Φ ∼10% over a range of excitation wavelengths),^31^ allowing spectroscopic assessment of loading, release, and intracellular accumulation, and it displays significant anticancer cytotoxicity. It is widely used in the clinic, with liposomal formulations such as Doxil (recognized as the first example of nanoparticulate drug delivery)^32^ and Myocet^33^ used against breast cancer, for example. There are a significant number of studies describing the delivery of Dox by a diverse range of MOFs—it is likely the most commonly used drug molecule in this area—and it has allowed exemplification of strategies such as targeted tumor uptake,^34^^,^^35^ stimuli-responsive release,^36^^,^^37^ multimodal treatments,^38^^,^^39^ and theranostics.^40^^,^^41^ A large number of these reports focus on the delivery of Dox from nanoparticles and composites of ZIF-8,^34^^,^^38^^,^^39^^,^^42^, ^43^, ^44^, ^45^, ^46^, ^47^, ^48^, ^49^, ^50^ in which tetrahedral Zn^2+^ centers connect 2-methylimidazolate linkers into a **sod** net,^51^ and UiO-66,^34^^,^^35^^,^^52^, ^53^, ^54^ in which Zr_6_O_4_(OH)_4_(RCO_2_)_12_ secondary building units (SBUs) connect 1,4-benzenedicarboxylate (BDC) linkers into a **fcu** net.^55^ In both cases, the small pore apertures—3.4 Å for ZIF-8 (up to 12.0 Å when flexibility^56^^,^^57^ is taken into account) and 6.0 Å for UiO-66—seemingly preclude penetration of the Dox molecule, which has a maximum diameter of 15.4 Å,^58^ into the porosity of the MOF. While some reports describe *in situ* encapsulation of Dox during the synthesis of these smaller-pore MOFs,^38^^,^^39^^,^^42^^,^^43^ the size disparity suggests it would be bound on external particle surfaces if loaded postsynthetically, which should modify release mechanisms. The localization of Dox on MOF nanoparticle surfaces would also have a significant impact on external surface modifications, which are often used to induce targeting or stimuli-responsive release mechanisms, but this is rarely discussed.^35^^,^^49^^,^^50^

In this study, we probe Dox (in the form of doxorubicin hydrochloride) loading and release using UiO-66, which has been studied intensely for drug delivery,^59^ as a small-pore MOF (ZIF-8 has been shown to have poor stability to pH < 7^60^ and in certain biological buffers^61^^,^^62^) and MIL-101(Cr), in which BDC linkers connect trimeric Cr_3_O(RCO_2_)_6_(H_2_O)_2_X (X = a monoanion) SBUs into the **mtn** topology with pore apertures of 12.0, 14.7, and 16.0 Å in diameter,^63^ as a large-pore MOF. There are a small number of studies into Dox uptake and delivery by MIL-101(Fe) and derivatives,^34^^,^^64^, ^65^, ^66^ one of which is clearly indicative of pore loading,^67^ but none into MIL-101(Cr). While there is a stigma regarding cytotoxicity associated with Cr, it is thought to be at the very least a nutritionally or pharmacologically beneficial factor or even an essential nutrient,^68^ and a small number of *in vitro*^30^^,^^69^^,^^70^ and *in vivo*^71^ studies on MIL-101(Cr) suggest good biocompatibility. Combined with its renowned chemical stability, which will preserve pore structure, it is an excellent candidate for these mechanistic studies. Using these two MOFs as DDSs, we show that RTCA can discriminate between the differing release mechanisms that result from contrasting Dox loading locations (external surface versus internal porosity), revealing Dox@MIL-101(Cr) as a potential controlled release chemotherapeutic and highlighting the need for multiple complementary *in vitro* experiments rather than endpoint assays in the development of novel DDSs.

## Results and Discussion

### MIL-101(Cr) and UiO-66 Nanoparticles’ Biocompatibility Assessment

UiO-66 and MIL-101(Cr) were obtained (Supplemental Experimental Procedures and Figures S1–S6) as crystalline nanoparticles after minor alterations to established literature procedures.^63^^,^^72^ The MOFs were highly crystalline and thermally stable (Figure S1), while size and external surface morphology characterization indicated that both MOFs were nanoparticulate (Figure S4). After synthesis and characterization, the biocompatibility of the bare MOF nanomaterials was studied *in vitro* using three established experimental cell cultures comprising healthy primary human dermal fibroblasts (HDFs), immortalized human embryonic kidney (HEK-293) cells, and human breast adenocarcinoma (MCF-7) cells (Table S1 and Figures S7 and S8).

Initially, RTCA was used to investigate MOF biocompatibility using the xCELLigence RTCA instrument and E-Plate VIEW 96-well plates. These 96-well electronic microtiter plates are covered with gold microelectrodes at the bottom of the wells, which allow an electrical signal, generated by the instrument, to pass through the electrodes. The presence of adherent cells at the bottom of the wells impedes the electrical signal; the higher the electrical impedance, the more cells are present. Noninvasive electrical impedance monitoring allows quantification of an instrument-generated cell index parameter, which reflects the surface area of the bottom of the well that is covered by cells and essentially the relative number of cells present. The cells were seeded in E-Plate VIEW 96-well plates, incubated at 37°C for 24 h, and the MOF nanoparticles were then administered as a suspension in complete cell culture media in a series of increasing doses (1, 10, and 50 μg mL^−1^). The cells were incubated in the presence of MOFs at 37°C, and cell proliferation, cell loss, cytostatic effects, morphology changes, and attachment quality following MOF addition were measured label-free and in real time for 3 days. Control experiments showed that the MOFs themselves did not interfere with the impedance measurements (Figure S7).

**Figure 1:**
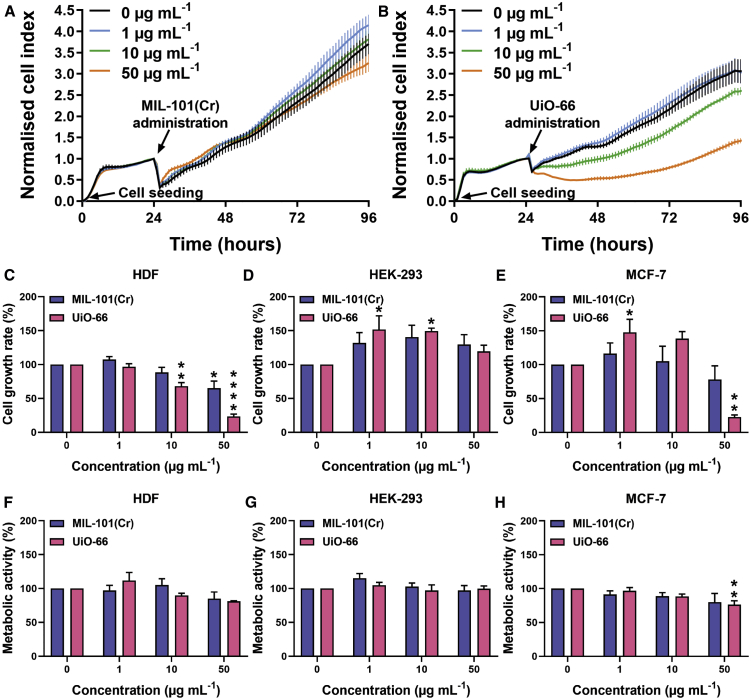
Biocompatibility Assessment of MOFs (A and B) Real-time cell analysis (RTCA) screening of (A) MIL-101(Cr) and (B) UiO-66 in HDFs. Cells were seeded at 0 h and MOFs were administered at 24 h. Cell growth was then monitored until 96 h. The data are presented as means ± SDs (n = 6). (C–E) The cell proliferative capacity was assessed by slope analysis of RTCA data from 24 to 96 h for (C) HDF, (D) HEK-293, and (E) MCF-7 cells. The data are presented as means ± SEMs. One-way ANOVA with Dunnett’s test (n = 3). (F–H) Direct comparison with cell metabolic activity, evaluated with Alamar blue assay, for (F) HDF, (G) HEK-293, and (H) MCF-7 cells, 72 h post-MOFs administration. Data are presented as means ± SEMs; One-way ANOVA with Dunnett’s test (n = 3). ∗p ≤ 0.05, ∗∗p ≤ 0.01, ∗∗∗p ≤ 0.001, and ∗∗∗∗p ≤ 0.0001.

Examples of the real-time data collected for HDF growth are given in Figure 1A (MIL-101(Cr)) and Figure 1B (UiO-66). Rather than compare individual cell growth plots, the RTCA data from these experiments and analogs using MCF-7 and HEK-293 cells (see Supplemental Information for RTCA traces) are interpreted using slope analysis from 24 to 96 h, to quantify cell proliferative capacity following MOF administration as a biocompatibility measure (Figures 1C–1E). Following MIL-101(Cr) administration, cell growth was found to be significantly inhibited only when the highest dose was administered in HDFs, and not at all in HEK-293 or MCF-7 cells. The cell growth rate matched that of the controls for all of the concentrations tested in HEK-293 and MCF-7 cells, suggesting that MIL-101(Cr) is not cytotoxic against these cell lines. For the HDF cells, MIL-101(Cr) did not affect proliferation when 1 and 10 μg mL^−1^ were administered, and cell growth was not significantly different from the untreated controls (Figure 1C). However, a MIL-101(Cr) concentration of 50 μg mL^−1^ had a slightly negative effect on the cell index.

Treatment of HDFs with UiO-66 nanoparticles did not cause any adverse effect at 1 μg mL^−1^, but treatment with higher doses (10 and 50 μg mL^−1^) resulted in a statistically significant decrease in cell proliferative capacity. Interestingly, in HEK-293 cells, the cell index was found to be increased after 1 and 10 μg mL^−1^ MIL-101(Cr) were administered and a significant increase in cell growth rate was calculated (Figure 1D). This was also observed in MCF-7 cells, following the addition of 1 μg mL^−1^ UiO-66 (Figure 1E). We believe this observation, however, is not due to the increased cell number, but is the result of changes in cell morphology, as we elucidate later in this study through flow cytometry experiments. More specifically, the size of these cells increases when nanoparticles are internalized and thus impede the electrical signal at a higher rate.

To compare RTCA with an endpoint assay and to study the effect of MOF administration on the metabolic activity of the three different cell lines, the Alamar blue assay was used (Figures 1F–1H). This assay is very similar to the widely used MTT/MTS assays, as it is based on the NAD(P)H-dependent metabolism of a reagent to generate a spectroscopic reporter; however, it is fluorescence based, whereas MTT/MTS assays are colorimetric, therefore precluding the possibility of interference by absorption from sedimented MOF particles. MIL-101(Cr) did not inhibit the metabolic activity of any of the 3 cell lines tested, while UiO-66 showed a statistically significant reduction in metabolic activity in MCF-7 cells when the highest concentration (50 μg mL^−1^) was administered (Figure 1H). In general, there was good correlation in biocompatibility between RTCA and the Alamar blue measurements for HEK-293 (Figures 1D and 1G) and MCF-7 cells (Figures 1E and 1H). In HDFs, however, the RTCA and subsequent slope analysis (Figure 1C) demonstrated some negative MOF-associated effects on cell growth that the Alamar blue assay (Figure 1F) failed to report. The metabolic assay did not show any significant adverse effects following MOF treatment, whereas the more sensitive RTCA indicated significant disturbances in cell growth. Overall, the results are broadly comparable, showing that MIL-101(Cr) seems to be better tolerated across the cell lines than UiO-66 under these conditions, and confirming that doses of 1 μg mL^−1^ (UiO-66) and 10 μg mL^−1^ (MIL-101(Cr)) are suitable for further drug delivery experiments. They do, however, highlight the power and sensitivity of RTCA in assessing biocompatibility, validating its efficacy, and confirm that multiple techniques should be used to assess *in vitro* cytotoxicity of nanomaterials rather than standard, single-point assays.

### MOFs Are Internalized by Cells and Enhance the Delivery of Calcein

**Figure 2:**
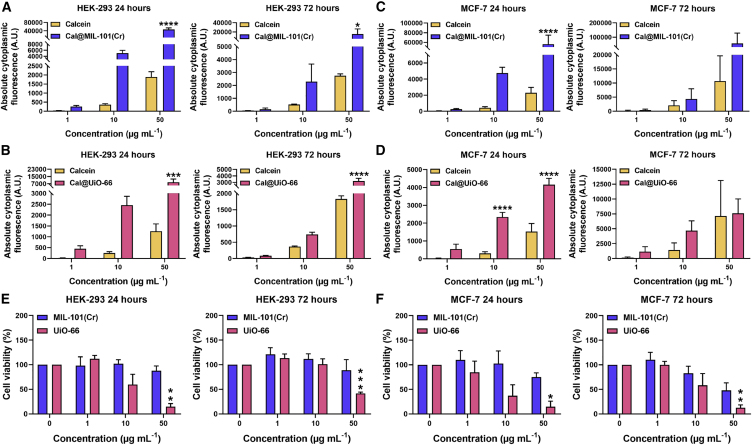
Cal@MIL-101(Cr) and Cal@UiO-66 Are Internalized by Cells and Enhance the Delivery of Calcein (A–D) Flow cytometry of free calcein versus Cal@MOF in HEK-293 cells, for (A) Cal@MIL-101(Cr) and (B) Cal@UiO-66, and in MCF-7 cells, for (C) Cal@MIL-101(Cr) and (D) Cal@UiO-66, measured 24 and 72 h post-administration. The concentration that was used for free calcein corresponds to the amount of calcein loaded in Cal@MIL-101(Cr) and Cal@UiO-66, respectively. The data are presented as means ± SEMs; 1-way ANOVA with Sidak’s test (n = 3). (E and F) Cell viability assessed with flow cytometry in (E) HEK-293 and (F) MCF-7 cells, 24 and 72 h post-MOF administration with Cal@MIL-101(Cr) and Cal@UiO-66. The data are presented as means ± SEMs; 1-way ANOVA with Dunnett’s test (n = 3). ∗p ≤ 0.05, ∗∗p ≤ 0.01, ∗∗∗p ≤ 0.001, and ∗∗∗∗p ≤ 0.0001.

The internalization of MOFs by HEK-293 and MCF-7 cells was investigated by flow cytometry, 24 and 72 h post-administration, using samples that had been calcein (Cal) stained using established protocols^73^^,^^74^ (Figures S9–S12; Tables S2 and S3). Both Cal@UiO-66 (4.8 wt% calcein loading) and Cal@MIL-101(Cr) (6.7 wt% calcein loading) were internalized by both cell types and with dose dependence, which is indicative of successful MOF internalization (Figure S13). In HEK-293 cells, higher levels of internalization were observed after 24 h of treatment compared to 72 h (Figures 2A and 2B), indicating that either the MOFs or their calcein cargo are being externalized over time, an effect that suggests the potential of the low bioaccumulation of MOFs in healthy tissue. In MCF-7 cells, higher internalization levels were observed after 72 h of treatment. This was attributed to the enhanced metabolism of cancer cells, an observation that makes MOFs very promising candidates for use in drug delivery, as higher cargo (i.e., drug) concentrations can be internalized over time (Figures 2C and 2D). In general, both calcein-loaded MOFs resulted in higher cargo internalization compared to the free calcein molecule; improved cargo internalization is essential in drug delivery, as lower drug doses can be used, minimizing unwanted side effects and off-target toxicity. Cal@MIL-101(Cr) outperformed Cal@UiO-66 at concentrations >10 μg mL^−1^, while their calcein delivery efficiency was similar for 1 μg mL^−1^, and the enhanced uptake compared to free calcein was more pronounced after 24 h, suggestive of rapid nanoparticle endocytosis by different mechanisms to the free molecule.

As calcein is not toxic to the cells (confirmed by the flow cytometry experiments with free calcein and previous work^73^^,^^74^) cell viability could also be determined using the flow cytometry data, through live cell number measurements. These values correlate well with the RTCA data and support the hypothesis that the increase in cell index measured by the technique was due to an increase in cell size, rather than an increase in the number of cells (Figures 2E and 2F). Following UiO-66 administration of 1 and 10 μg mL^−1^ in both HEK-293 and MCF-7 cells, the number of cells remained the same as that of the controls, indicating that there was no increase in proliferation rate, despite the apparent increase in growth rate by RTCA. In both cell lines, 50 μg mL^−1^ UiO-66 caused a significant reduction in cell viability and increase in cell loss from as early as 24 h post-treatment. The cell cultures did not recover fully, and this effect was still observable after 72 h (Figures 2E and 2F). MIL-101(Cr), however, did not significantly affect cell viability. For both cell lines and for all of the concentrations that were tested, cell viability matched that of the controls at both time points of investigation. An increase in cell size as a result of MOF internalization was again observed with RTCA in these two cell types following MIL-101(Cr) administration, but this did not produce statistically significant changes in the overall cell growth rate calculation (Figures 1D and 1E). Overall, the flow cytometry data at these two time points correlate well with the RTCA data, further validating its use for assessing biocompatibility.

### Dox Loading in UiO-66 and MIL-101(Cr)

**Figure 3:**
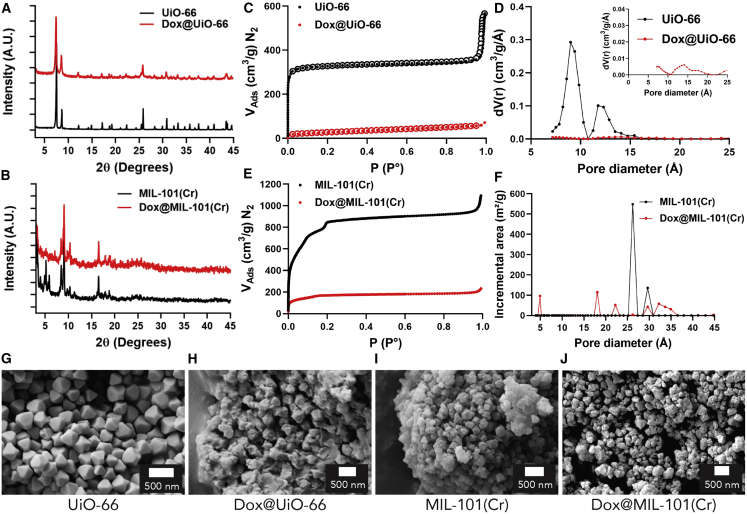
Characterization of Dox@MOF Nanoparticles (A and B) PXRD patterns of (A) UiO-66 and (B) MIL-101(Cr) MOF nanoparticles, before and after Dox loading. (C) N_2_ adsorption isotherm (77 K) of UiO-66 and Dox@UiO-66. (D) Pore size distribution plots taken from these isotherms, with a scaled-up trace for Dox@UiO-66 given as an inset (carbon, slit pore, quenched solid density functional theory [QSDFT], equilibrium calculation model). (E) N_2_ adsorption isotherm (77 K) of MIL-101(Cr) and Dox@MIL-101(Cr). (F) Pore size distribution plots taken from these isotherms (N_2_@77 carbon cylinder pores, multi-wall nanotube [MWNT], non-local DFT [NLDFT] calculation model). (G–J) SEM images of (G) UiO-66, (H) Dox@UiO-66, (I) MIL-101(Cr), and (J) Dox@MIL-101(Cr). Scale bars represent 500 nm.

After the biocompatibility, internalization, and efficacy of cargo delivery by MIL-101(Cr) and UiO-66 were studied, their drug delivery potential and modes of action were tested. Dox was chosen as the chemotherapeutic agent, due to its clinical use against a broad spectrum of cancers, and it is also one of the few anticancer drugs currently administered as part of a DDS.^32^^,^^33^ Both MOFs were postsynthetically loaded with Dox by immersion in a solution in Tris-buffered saline, to yield Dox@MIL-101(Cr) and Dox@UiO-66 (Figures S14–S20; Tables S4–S8). Successful drug loading was indicated by a change in nanoparticle color, from white to red for UiO-66 and from green to red for MIL-101(Cr) (Figure S14). Although some minor peak broadening was observed in the powder X-ray diffraction (PXRD) patterns for both MOFs, no extra peaks were observed, confirming that Dox did not co-crystallize with the MOF nanoparticles (Figures 3A and 3B).

The Dox loading for each MOF was calculated by ultraviolet-visible light (UV-vis) spectroscopic analysis of supernatants, and the drug-loading capacity of the materials was calculated based on grams of drug per grams of drug-loaded material (wt%). This was calculated at 58 wt% for UiO-66 and 10 wt% for MIL-101(Cr), and the difference in loading values clearly indicates different loading mechanisms. The value for MIL-101(Cr) is commensurate with previous work on MIL-101(Fe) and derivatives (11–13 wt%),^64^^,^^65^ while similar very high Dox-loading values have also been reported for UiO-66,^52^ even on samples with previously modified external surfaces.^35^ By thermogravimetric analysis (TGA) analysis, an increase in the organic content was obvious in both cases, indicative of successful drug loading (Figure S16). For Dox@UiO-66, thermal degradation was observed at a lower temperature than the non-loaded MOF, a general trend that has been observed for surface functionalized MOFs.^72^^,^^75^ It is hypothesized that, due to the added organic functionality at the external surface having a lower thermal stability, combustion is initiated at a lower temperature, and that triggers earlier thermal decomposition of the material as a whole. For Dox@MIL-101(Cr) however, thermal decomposition began almost at the same stage as MIL-101(Cr), but the overall MOF degradation occurred at a slightly higher temperature. We hypothesize that this observation is indicative of different modes of Dox loading across the two MOFs; MIL-101(Cr) has large enough pores to accommodate the Dox cargo, and having loaded pores could contribute extra thermal stability to the structure.

To further understand the drug-loading mechanism in each instance, the Brunauer-Emmett-Teller (BET) surface area of the drug-loaded materials was measured from N_2_ adsorption isotherms at 77 K and compared against their non-loaded counterparts. In the case of Dox@UiO-66, the BET surface area showed a dramatic decrease from 1,324 m^2^ g^−1^ for the empty nanoparticles to 95 m^2^ g^−1^ for the drug-loaded MOFs **(**Figure 3C). Bearing in mind that 58 wt% of the Dox@UiO-66 is non-porous Dox, and therefore a significant decrease in gravimetric surface area would be expected in any case, it is clear that the majority of the porosity is blocked by the accumulation of Dox on the external surface of UiO-66; pore loading to this extent would completely remove any residual porosity. This was further validated by examination of the pore size distribution plot (Figure 3D). The average primary pore diameter for the unloaded material was 13 Å and for the secondary pore it was ∼9 Å, correlating well with the theoretical values of 11 and 8 Å, respectively. For Dox@UiO-66, however, while the experimental pore size volume of this material was essentially zero, a small trace with similar pore dimensions could be observed, suggestive of blocking access to the majority of pores rather than modifying their geometry through occupation and further validating that Dox molecules have blocked the porosity of UiO-66 by covering its external surface. For MIL-101(Cr), a decrease in its BET surface area from 3,041 to 623 m^2^ g^−1^ was observed after drug loading (Figure 3E). The fact that a significant proportion of the internal porosity is still accessible after drug loading suggests that, for Dox@MIL-101(Cr), Dox did not completely cover the external surface of the nanoparticles and block pore access, but penetrated the porosity without completely filling it, although some external surface deposition cannot be ruled out. This correlates with the TGA observations, and is further confirmed by the pore size distribution graph of Dox@MIL-101(Cr), where the main peak at 26 Å (representative of the 25 Å pore) in the bimodal distribution disappears and smaller residual peaks appear (Figure 3F), again suggestive of Dox localization within the pores.

A difference in drug-loading mode between the two materials is suggested by the physical characterization data and is also evident by scanning electron microscopy (SEM). The octahedral morphology of UiO-66 (Figure 3G) changes dramatically on loading, with Dox forming visible layers on the external surfaces of the UiO-66 nanoparticles (Figure 3H). For MIL-101(Cr), the MOF morphology (Figure 3I) was maintained on the formation of Dox@MIL-101(Cr), with Dox being mostly encapsulated inside the pores and not notably visible on the external surface of the material (Figure 3J). This is a plausible physicochemical characteristic for such MOFs, as the Dox molecule is too large (maximum diameter 15 Å)^58^ to fit the pores of UiO-66 (pore window 6 Å). Therefore, any attachment to the nanoparticles occurs on their external surfaces, but can penetrate MIL-101(Cr), whose largest pore window is 16.0 Å in diameter, and thus Dox internalization is possible. In addition, the high affinity of Dox for the surface of UiO-66 could be a consequence of electrostatic interactions between the negatively charged surface of UiO-66, as confirmed by zeta potential measurement (Figure S6), and a cargo that would be expected to have residual protonation at neutral pH. In contrast, MIL-101(Cr) was measured to have a positive surface potential. We hypothesize that this major difference in drug-loading modes could result in different drug delivery and cytotoxic efficiency mechanisms between the two systems.

To validate our hypothesis, we used grand canonical Monte Carlo (GCMC) simulations to investigate Dox loading (see Supplemental Experimental Procedures).^76^ When exploring Dox loading in a UiO-66 model devoid of crystalline defects and completely activated, we observed zero uptake as a consequence of the size mismatch between the adsorbate and the pore cavities (i.e., Dox molecules do not fit inside the microporosity of UiO-66). Importantly, a GCMC simulation cannot distinguish between open and closed porosity since the molecules are inserted inside the pores and do not need to be transported, unlike in experiments, though the pore windows. This observation strongly suggests that the loading must occur through adsorption on the external surface of the UiO-66 particles. In contrast, simulations on MIL-101(Cr) yielded a theoretical maximum loading of 1.16 g of Dox per 1 g MOF, which is ∼10 times higher than what we observed experimentally, confirming that the pore adsorption of Dox is possible in MIL-101(Cr). It should be noted that, when running these simulations, we did not take into account competing solvents. Although previous simulations showed that not including the solvent in similar systems had a negligible effect,^76^^,^^77^ this could explain why experimental loading values are lower than the theoretical maximum. In any case, it is also noteworthy that the Dox theoretical maximum loading only occupies 58% of the available pore volume in MIL-101(Cr) due to inefficient packing of the rigid Dox molecule. This suggests that, in Dox@MIL-101(Cr), only 5.3% of the total pore volume is occupied, explaining the sizeable remaining porosity observed in its N_2_ adsorption isotherm. These simulations clearly support the hypothesis of external surface loading in Dox@UiO-66 and pore loading in Dox@MIL-101(Cr).

### Delivery Profiles of Dox@MIL-101(Cr) and Dox@UiO-66

For the drug delivery evaluation experiments, the highest completely non-toxic concentration of each MOF was used, 1 and 10 μg mL^−1^ for UiO-66 and MIL-101(Cr), respectively. This entailed concentrations of 2.38 μg mL^−1^ Dox@UiO-66 and 11.1 μg mL^−1^ Dox@MIL-101(Cr) being used. In a similar manner, the concentrations of equivalent amounts of free Dox were calculated to be 1.38 μg mL^−1^ for Dox@UiO-66 and 1.1 μg mL^−1^ for Dox@MIL-101(Cr). Therefore, due to the significantly lower drug-loading capacity of Dox@MIL-101(Cr) compared to Dox@UiO-66, both systems could essentially transfer a similar amount of Dox to the cells at these concentrations. To identify the mode of action of the different DDSs, the effect of Dox@MOFs administration over time was monitored by RTCA for 72 h in 3 different cancer cell lines, comprising MCF-7, HepG2, and human ovarian carcinoma (A2780ADR) (see Supplemental Experimental Procedures).

**Figure 4:**
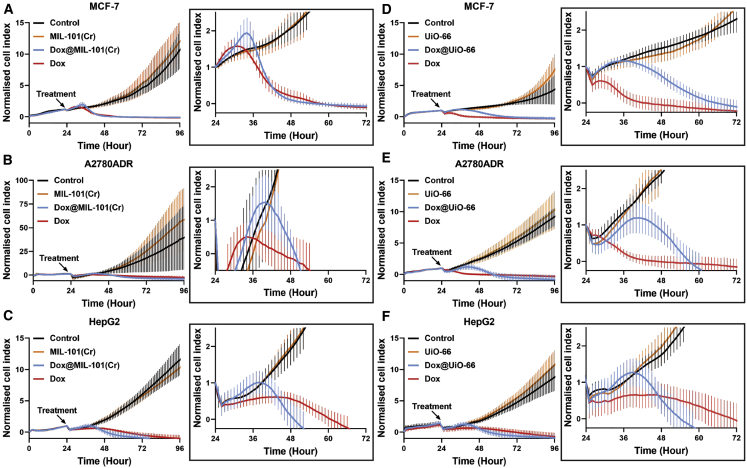
Dox@MIL-101(Cr) and Dox@UiO-66 Are Therapeutically Efficacious and Have Different Dox Delivery Profiles (A–C) RTCA of 3 cancer cell lines treated with empty MIL-101(Cr), Dox@MIL-101(Cr), and free Dox. The cancer cell lines were allowed to grow for 24 h after the initial cell seeding at time point 0. Treatment administration took place at 24 h. The data are presented as means ± SDs (n = 6). (D–F) RTCA of 3 cancer cell lines treated with empty UiO-66, Dox@UiO-66, and free Dox. The cancer cell lines were allowed to grow for 24 h after the initial cell seeding at time point 0. Treatment administration took place at 24 h. The data are presented as means ± SDs (n = 6).

The administration of free Dox inhibited cell growth quickly, at ∼4–12 h after addition depending on cell line, and killed all the cells present in the cultures (Figure 4). Dox@MIL-101(Cr) treatment, in comparison to free Dox, exhibited a small delay of 10–16 h before cytotoxicity was observed (Figures 4A–4C). After an immediate decrease in the cell index due to the agitation of the well plates upon the addition of material, which occurs in all of the experiments, an initial small increase in the cell index following Dox@MIL-101(Cr) administration was observed, which closely matched that of the empty MIL-101(Cr) control. This can be attributed to cells internalizing the drug-loaded nanoparticles and causing an increase in cell size, as well as uninhibited cell growth before Dox release. Hence, the observed delay in cytotoxicity is explained by the necessity for the release of the Dox cargo inside the cells and the subsequent delay in its transfer into the cell nucleus, where Dox is active. Nevertheless, once sufficient amounts of the Dox reached the cell nucleus, cell death ensued and the cell index quickly returned to a profile aligned with that of free Dox, or below it.

In contrast, Dox@UiO-66 demonstrated a significantly longer delay in mediating cytotoxicity in all of the cancer cell lines tested (Figures 4D–4F), compared to both free Dox and Dox@MIL-101(Cr). After the addition of Dox@UiO-66, the cell index continued to increase for 14–20 h, depending on the cell line, similar to the controls. This increased uninhibited cell growth period could be attributed to a slower release rate of Dox. The initial cell growth was eventually followed by inhibition, a subsequent cytotoxicity-associated decrease in cell index, and finally, cell death. The cell index profiles returned to track those of free Dox after ∼36 h incubation for MCF-7 (Figures 4A and 4D) and A2780ADR (Figures 4B and 4E) cells, and after ∼24 h incubation for HepG2 cells (Figures 4C and 4F). It is notable that the difference in cytotoxicity onset time between the two Dox-loaded MOFs is less significant for HepG2 cells, but still apparent, and both drug-loaded MOFs induce cytotoxicity faster overall compared to free Dox. Complete cell death takes ∼24–26 h for Dox@MIL-101(Cr) and 34–36 h for Dox@UiO-66. Single-point assays are typically measured 24 and 72 h after incubation with DDSs; it is, therefore, worthwhile to note that, in this case, assays after 24 h of incubation would suggest some cell-specific selectivity in anticancer efficacy for Dox@UiO-66, despite this not being true overall, and if assessing cytotoxicity after 72 h this difference in behavior between Dox@UiO-66 and Dox@MIL-101(Cr) would not have been apparent. Nevertheless, both materials were therapeutically efficacious in facilitating cancer drug delivery and cytotoxicity toward cancer cells.

Since both materials are very efficiently internalized by cells (Figure 2), their different time-dependent cytotoxicities could be attributed to different Dox release mechanisms. The apparent faster drug release from Dox@MIL-101(Cr) compared to Dox@UiO-66 is likely a consequence of the different mode of drug loading in each system. In the instance of Dox@UiO-66, Dox is deposited primarily on the outer surface of the nanoparticles. The particles, therefore, present an external surface layer of insoluble, aggregated Dox, which is likely to be difficult to digest once inside the cells, as this process would involve disassociation/dissolution of the strongly aggregated drug molecules. For Dox@MIL-101(Cr), Dox is primarily located inside the porosity of the framework and presumably well dispersed, as it only takes up a small fraction of the pore volume, making its release easier and faster, either through desorption or intracellular digestion of the MOF. To test this hypothesis, we used time-dependent confocal microscopy to monitor the intracellular release and subsequent nuclear localization of Dox from Dox@MIL-101(Cr) and Dox@UiO-66 in MCF-7 cells.

### Dox@MIL-101(Cr) Enhances Delivery and Subsequent Release of Dox in MCF-7 Cells

**Figure 5:**
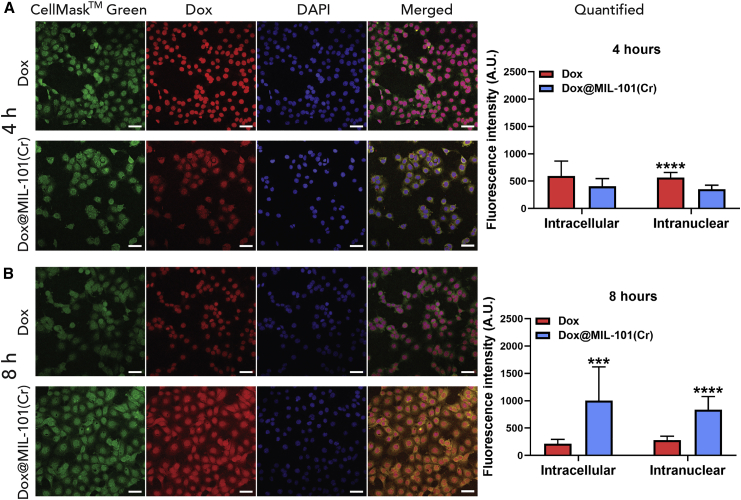
Dox@MIL-101(Cr) Enhances the Delivery and Subsequent Release of Dox in MCF-7 Cells Confocal microscopy and fluorescence quantification of free Dox and Dox@MIL-101(Cr) delivery in MCF-7 cells (A) 4 and (B) 8 h post-treatment administration. The concentration that was used for free Dox corresponds to the amount of Dox loaded in Dox@UiO-66. Green: cell membrane stain, red: Dox, blue: nuclear DAPI stain. Scale bars, 50 μm. Quantification of Dox delivery capacity through measurements of total intracellular and intranuclear fluorescence. The data are presented as means ± SDs; Student’s t test (n = 10). ∗p ≤ 0.05, ∗∗p ≤ 0.01, ∗∗∗p ≤ 0.001, and ∗∗∗∗p ≤ 0.0001 (comparison between Dox and Dox@MIL-101(Cr) treatments).

Quantification of the intracellular and intranuclear fluorescence intensity of Dox by confocal microscopy (see Supplemental Experimental Procedures), following administration in MCF-7 cells as a free agent or as part of the MIL-101(Cr) drug delivery system, was performed at both 4 and 8 h post-administration, as RTCA demonstrated that significant cytotoxicity becomes apparent at ∼12 h after incubation. After 4 h of treatment, the intracellular concentration of Dox was equivalent for both the free molecule and the DDS (Figure 5A). Nuclear quantification at 4 h revealed that intranuclear Dox was significantly higher for the free molecule, co-localizing with the DAPI stain, while Dox@MIL-101(Cr) could be seen in both cytoplasmic and nuclear localization. The red emission is visually more diffuse across the cell for Dox@MIL-101(Cr) compared to free Dox, but this is not directly reflected in our quantified data, as the intensity of Dox fluorescence only from the cytoplasm cannot be measured with a high level of confidence by our experimental protocol. This is consistent with Dox being held within the nanocarrier in the cell cytoplasm and then migrating to the nucleus on release, and in keeping with a thesis of an immediate cytotoxic effect for the free drug, supporting our hypothesis that a drug-release step delays Dox@MIL-101(Cr)-mediated cytotoxicity. At 8 h post-treatment, both intracellular and intranuclear Dox concentrations were significantly higher for Dox@MIL-101(Cr), outperforming the free molecule (Figure 5B). The exceptional internalization capacity of MIL-101(Cr), along with the relatively rapid intracellular Dox release, result in higher amounts of Dox reaching the nucleus when administered as part of the DDS compared to free molecule administration. This suggests that lower concentrations of Dox could be used, and therefore its intrinsic off-target toxicity could be minimized or avoided.

### Dox@UiO-66 Delays the Delivery and Subsequent Release of Dox in MCF-7 Cells

**Figure 6:**
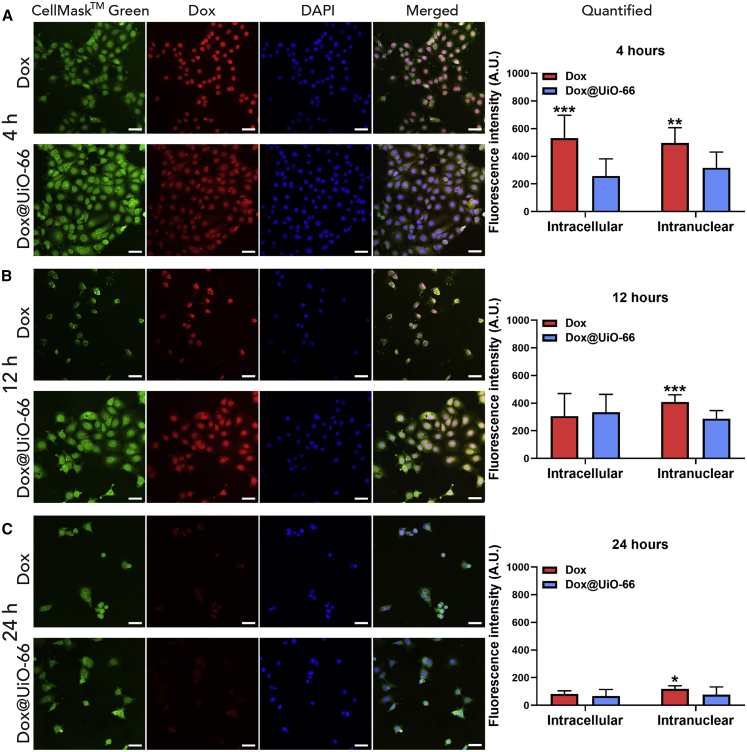
Dox@UiO-66 Delays the Delivery and Subsequent Release of Dox in MCF-7 Cells (A–C) Confocal microscopy and fluorescence quantification of free Dox and Dox@UiO-66 delivery in MCF-7 cells (A) 4, (B) 12, and (C) 24 h post-treatment administration. The concentration that was used for free Dox corresponds to the amount of Dox loaded in Dox@UiO-66. Green: cell membrane stain, red: Dox, blue: nuclear DAPI stain. Scale bars, 50 μm. Quantification of Dox delivery capacity through measurements of total intracellular and intranuclear fluorescence. The data are presented as means ± SDs; Student’s t test (n = 10). ∗p ≤ 0.05, ∗∗p ≤ 0.01, ∗∗∗p ≤ 0.001 (comparison between Dox and Dox@UiO-66 treatments).

As with Dox@MIL-101(Cr), quantification of the intracellular and intranuclear Dox levels in MCF-7 cells after incubation with Dox@UiO-66 was achieved by calculation of the fluorescence intensity of Dox after co-localization with the cytoplasmic or nuclear dye, respectively (see Supplemental Experimental Procedures). After 4 h, the absolute intracellular fluorescence value of free Dox was significantly higher when administered as a free drug compared to the DDS (Figure 6A). However, after 12 (Figure 6B) and 24 h (Figure 6C) of treatment (times based on the delay and onset of cytotoxicity by RTCA), there was no statistical difference in Dox fluorescence between the free drug and Dox@UiO-66. This indicates that prolonged incubation times yield equally potent intracellular Dox concentrations as the free drug.

In addition, at all time points, free Dox was found primarily in the cell nucleus, strongly co-localizing with the nuclear stain, while Dox from Dox@UiO-66 was seen in both the nucleus and the cytoplasm of the cells, with a diffuse red emission similar to Dox@MIL-101(Cr), and indicative of Dox being associated with the nanocarrier in the cytoplasm before or during release. These observations suggest that Dox@UiO-66 releases Dox slowly into the cytoplasm, notably slower than Dox@MIL-101(Cr), further strengthening our hypothesis that the delayed cytotoxic effects observed with RTCA are associated with the drug-release profile of the material in question. The difference in intracellular Dox release kinetics points to different release mechanisms for the two MOFs, which reflects the fact that Dox is loaded in the internal porosity of Dox@MIL-101(Cr) and on the external surface of Dox@UiO-66. Moreover, at all time points, Dox demonstrated higher nuclear internalization when administrated as a free molecule than as part of Dox@UiO-66, explaining the faster cytotoxic effect observed previously with the RTCA experiments.

By using a suite of complementary *in vitro* techniques, we have shown that RTCA is a highly informative technique for assessing the biocompatibility of nanoscale MOFs. Proof-of-concept work has shown that MIL-101(Cr) and UiO-66 have excellent biocompatibility, internalization efficiency, and significantly enhanced calcein cargo delivery across a series of cell types. Dox was chosen as a chemotherapeutic probe for drug delivery, and was found to attach to the external surface of UiO-66 nanoparticles, forming layers on top of them, while the larger pore cavities of MIL-101(Cr) allowed for penetration to the internal porosity. RTCA was used to monitor drug delivery, demonstrating that these differences in loading have a direct effect on the drug-release rate within cancer cells, affecting cytotoxic efficacy in a time-dependent manner that could easily be missed by the single-point assays commonly used to probe cytotoxicity. Confocal fluorescence microscopy confirmed different intracellular Dox release rates that closely corresponded to the RTCA measurements. Due to the Dox loading mode of Dox@UiO-66, drug release more likely resembles the dissolution of a Dox nanoparticle than the degradation of the MOF carrier, subsequently delaying the release of the drug and its cytotoxic action. Conversely, Dox@MIL-101(Cr) nanoparticles display a more rapid drug release, occurring via a combination of Dox desorption and degradation of the MOF carrier, leading to a significantly large intracellular accumulation of Dox compared to the free drug. The characterization of drug loading in MOFs rarely extends to pinpointing its locality; our data indicate that the relationship between drug-loading mode and MOF-mediated drug delivery clearly must be taken into consideration in the development of therapeutically efficacious MOF DDSs, and that surface loading Dox on MOF nanoparticles may explain slow release trends observed previously.

This more controlled killing effect of Dox@MOFs, in contrast to the immediate effect of the free cancer drug, makes the translatability of these materials very promising for use as DDSs. As current DDSs do not typically achieve enhanced Dox efficiency, MIL-101(Cr) is a very promising candidate for this type of application, as higher levels of Dox internalization can be achieved. This indicates that a lower drug concentration could be used, allowing for the development of a therapeutic strategy with the potential to limit the undesirable off-target acute toxicity of chemotherapy. It is also compatible with locoregional drug administration strategies, such as in head and neck cancers, where it may enable the sustained local release of a chemotherapeutic while limiting collateral damage to surrounding healthy tissue. While these proof-of-concept mechanistic experiments have been carried out on bare MOFs, the pore loading of Dox in MIL-101(Cr) is also compatible with further functionalization to enhance targeting and biodistribution as we move toward *in vivo* experiments to assess clinical translatability.

## Experimental Procedures

### Resource Availability

#### Lead Contact

Further information and requests for resources and reagents should be directed to and will be fulfilled by the Lead Contact, Prof. Ross Forgan (ross.forgan@glasgow.ac.uk).

#### Materials Availability

All solvents and reagents were purchased from Alfa Aesar, Acros Organics, Sigma-Aldrich, Merck, Tokyo Chemical Industry, Thermo Fisher Scientific, and Zymo Research USA, and used without further purification (see Supplemental Experimental Procedures).

#### Data and Code Availability

All of the data are presented within the article and Supporting Information and are available to download from https://dx.doi.org/10.5525/gla.researchdata.1072.

### Synthesis and Characterization

All of the experimental procedures are listed in the Supplemental Experimental Procedures.

## Supplemental Information

Document S1. Supplemental Experimental Procedures, Figures S1–S20, and Tables S1–S8

Document S2. Article plus Supplemental Information
