# Supplementary material for: Identifying Differing Intracellular Cargo Release Mechanisms by Monitoring In Vitro Drug Delivery from MOFs in Real Time
Source: Cell Rep Phys Sci. 2020 Nov 18;1(11):100254. doi: 10.1016/j.xcrp.2020.100254 (PMC7674849; doi:10.1016/j.xcrp.2020.100254)
Supplement: Document S1. Supplemental Experimental Procedures, Figures S1–S20, and Tables S1–S8 [file mmc1.pdf]

Cell Reports Physical Science, Volume *1*

Supplemental Information

Identifying Differing Intracellular Cargo Release Mechanisms by Monitoring *In Vitro* Drug Delivery from MOFs in Real Time

Panagiota Markopoulou, Nikolaos Panagiotou, Aurelia Li, Rocio Bueno-Perez, David Madden, Sarah Buchanan, David Fairen-Jimenez, Paul G. Shiels, and Ross S. Forgan

SUPPLEMENTAL EXPERIMENTAL PROCEDURES

General Experimental Remarks

Powder X-ray diffraction (PXRD): PXRD measurements were carried out at 298 K using a PANalytical X'Pert PRO diffractometer (λ (CuKα) = 1.4505 Å) on mounted bracket sample stage. Data were collected over the range 3-45˚. PXRD patterns were predicted from single crystal data using Mercury 4.0.[S1]

Thermogravimetric analysis (TGA): Measurements were carried out using a TA instruments Q500 Thermogravimetric Analyser. Measurements were collected from room temperature to 800˚C with a heating rate of 10˚C / min under an air atmosphere. Data were analysed with TA Instruments Universal Analysis 2000.

Gas uptake: For UiO-66 samples, N_2 adsorption isotherms were carried out at 77 K on a Quantachrome Autosorb IQ gas sorption analyser. Samples were degassed under vacuum at 393 K for 20 h on the instrument prior to analysis. For MIL-101(Cr) samples, N_2 isotherms were collected using a Micromeritics 3Flex at 77 K. Prior to analysis, samples were degassed in a vacuum oven at 393 K for 12 h. In situ degas (393 K, 12 h) was further performed after sample loading into the instrument,

Pore size distribution: : For UiO-66 samples, pore size distributions were calculated using the N_2 at 77 K on a carbon (slit pore, QSDFT, equilibrium model) calculation model within the Quantachrome ASiQwin operating software. For MIL-101(Cr) samples, the N_2@77-Carb Cyl Pores, MWNT, NLDFT wthin the Micromeritics operating software was used.

Dynamic light scattering (DLS): Colloidal stability analysis was performed by DLS with a Zetasizer Nano ZS potential analyser equipped with non-invasive Backscatter optics (NIBS) and 50 mW laser at 633 nm. Data were analysed with Zetasizer Nano software v3.30.

Scanning electron microscopy (SEM): The powder samples were deposited onto carbon tabs and coated with Pd for 150 seconds using Polaron SC7640 sputter coater and imagined using a Carl Zeiss Sigma Variable Pressure Analytical SEM with Oxford Microanalysis. Particle size distribution was analysed manually using ImageJ v1.52 software.

Fourier transform infrared spectroscopy (FT-IR): Infrared spectra of solids were collected using Schimadzu Fourier Transform Infrared Spectrometer, FTIR-8400S, fitted with a Diamond ATR unit.

UV-Vis spectroscopy: UV-Vis spectra were recorded using a Schimadzu UV-1800 and analysis was carried out using the software UVProbe v2.51. In some cases, a ThermoFischer Nanodrop One was used (stated) and data were analysed with Graphpad Prism 7.

Real time cell analysis (RTCA): RTCA cell proliferation assays were performed on an xCELLigence® RTCA MP machine, with E-plate VIEW 96. The RTCA normalised cell index over time data, which were collected, were analysed with the use of the RTCA software version 1.2.1 (ACEA Biosciences Inc.).

Plate reader: The UV-Vis spectrometry and fluorescence intensity experiments for the biological assays were performed on a CLARIOstar plate reader (BMG LABTECH). Data were analysed with Graphpad Prism 7.

Flow cytometry: All the flow cytometry experiments were carried out with an Attune NxT (Thermo Fischer). Excitation laser 488 nm, configuration filters FITC 530/30 nm and PerpCP-Cy5.5 695/40 nm. The analysis was carried out with FCS express 6 software.

Confocal microscopy: For confocal microscopy, a ZEISS LSM 780 Confocal microscope was employed. Images were captured with the aid of ZEN black software. Analysis was performed with the custom Fiji macro BatchQuantify software.[S2]

MOF Synthesis and Characterisation

All solvents and reagents were purchased from Alfa Aesar, Acros Organics, Sigma-Aldrich, Merck, Tokyo Chemical Industry, Thermo Fischer Scientific and Zymo Research USA, and used without further purification. All MOFs were dried *in vacuo* at room temperature for at least 24 hours and subsequently in the oven at 120 ˚C overnight before any characterisation or further experiments took place.

Synthesis of UiO-66: Zirconium (IV) chloride (0.6839 g, 3 mmol) was dissolved in 30 mL DMF (sonication until complete dissolution). In a separate container, terephthalic acid (0.4486 g, 3 mmol) was dissolved in 30 mL DMF. The two solutions were mixed in a glass pyrex jar, 4.2 mL (7% tot. vol.) acetic acid was added, and the jar was heated at 120 ˚C for 24 hours. The jar was allowed to cool down to ambient temperature, the nanoparticles were collected by centrifugation (4500 rpm for 20 minutes), washed with DMF (×2) and MeOH (×3) (sonication and centrifugation cycles) and were dried *in vacuo* for 72 hours. This synthesis was adapted from a literature source.[S3]

Synthesis of MIL-101(Cr): Chromium (III) nitrate nonahydrate (1.6 eq, 8 mmol) was dissolved in 25 mL deionised water (sonication until complete dissolution). Then, terephthalic acid (1 eq, 5 mmol) was added and the suspension was sonicated for another 10 minutes. Acetic acid (1 eq, 5 mmol) was added and the suspension was transferred into a Teflon lined steel autoclave bomb and was heated at 220 °C for 6 hours. The autoclave bomb was left to cool down to ambient temperature and the nanoparticles formed were collected with centrifugation and dried *in vacuo* for 24 hours. The solid was loaded into a 100 mL round bottom flask and 50 mL DMF were added. The suspension was refluxed under stirring at 140 °C for 4 hours. Then it was centrifuged and dried *in vacuo* overnight. The nanoparticles were washed once with DMF and once with MeOH (sonication and centrifugation cycles) and were dried *in vacuo* for 72 hours. This synthesis was adapted from a literature source.[S4]

Powder X-Ray Diffraction (PXRD): The first part of characterisation of the materials involved verifying their crystallinity and validating their identity. Both goals were achieved with powder X-ray diffraction (PXRD). **Figure S1** shows the PXRD patterns of the materials compared to the calculated patterns based on the crystal structures found in the Cambridge Crystallographic Data Centre (CCDC) database. The high crystallinity of all the samples is prominent and a very close match of the experimentally acquired patterns to the calculated ones is obvious in both cases.

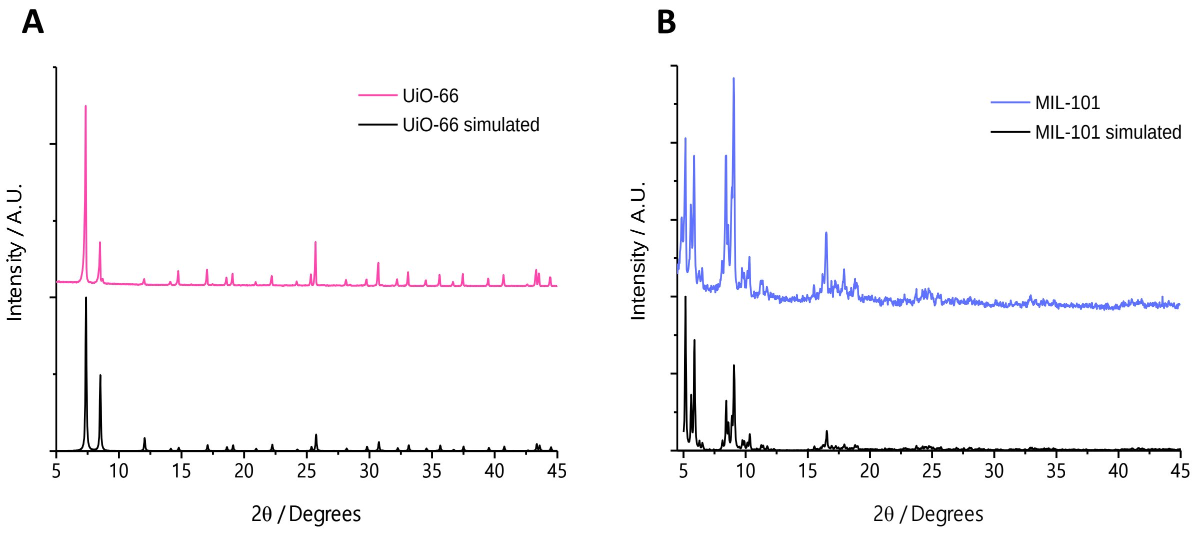

Figure S1: PXRD patterns of the as synthesised MOFs compared to their corresponding calculated patterns. (A) UiO-66. (B) MIL-101(Cr).

Thermogravimetric Analysis (TGA): The thermal stability of the materials was tested with TGA in air. As expected, UiO-66 was the sample with the higher thermal stability with a thermal decomposition temperature of 534 °C, which also matches the thermal stability reported for this material in the literature.[S3] There was not a mass loss step before 200 °C, which indicated that there were no residues of the synthesis or washing solvents. A small mass loss step was present at 250–300°C. This is characteristic for the UiO family MOFs and is attributed to the dehydration of the zirconium oxoclusters, $[Zr_6O_4(OH)_4L_6]$ (where L=ligand) losing two water molecules and adopting the formation $[Zr_6O_6L_6]$.[S5] The thermal decomposition of MIL-101(Cr) started at approximately 345 °C. Cr^{3+} MOFs are generally very chemically stable as well, due to Cr^{3+} being a hard Lewis acid and therefore having a favourable stable bond with the hard Lewis basic groups of the carboxylate linker. A mass loss step of 7.5% was present at a lower temperature. This was due to evaporation of adsorbed ambient moisture **(Figure S2)**. This mass loss step could not correspond to residual DMF (synthesis solvent) as it begins at ambient temperature which is very low for DMF evaporation. The chance for this step corresponding to residual methanol (washing solvent) was also eliminated as the material was thoroughly dried *in vacuo* and at 120 °C before the characterisation took place. However, the sample was not stored *in vacuo* or under dry conditions and therefore ambient moisture could be adsorbed. This was also confirmed by FT-IR spectroscopy where the characteristic peak of absorbed water is present at 3250 cm^{-1} **(Figure S3)**.

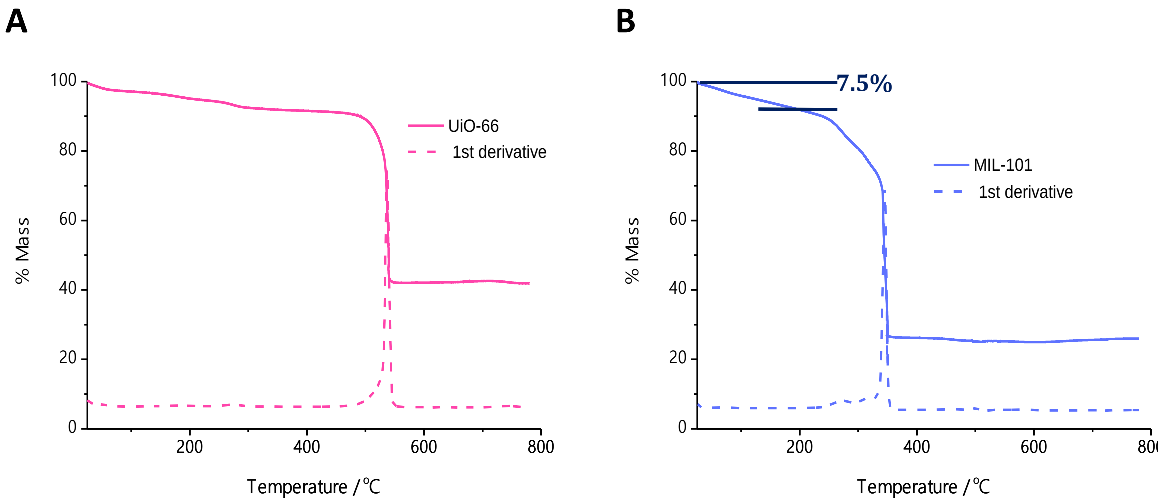

Figure S2: TGA profiles and first derivative. (A) UiO-66 and (B) MIL-101(Cr).

FT-IR: Further characterisation of the materials was achieved with solid state FT-IR. from the FT-IR spectra, the absence of solvent traces was further validated. A wide peak at approximately 3000-3500 cm^{-1} is present, this corresponds to adsorbed water on the MOF structure **(Figure S3)**.

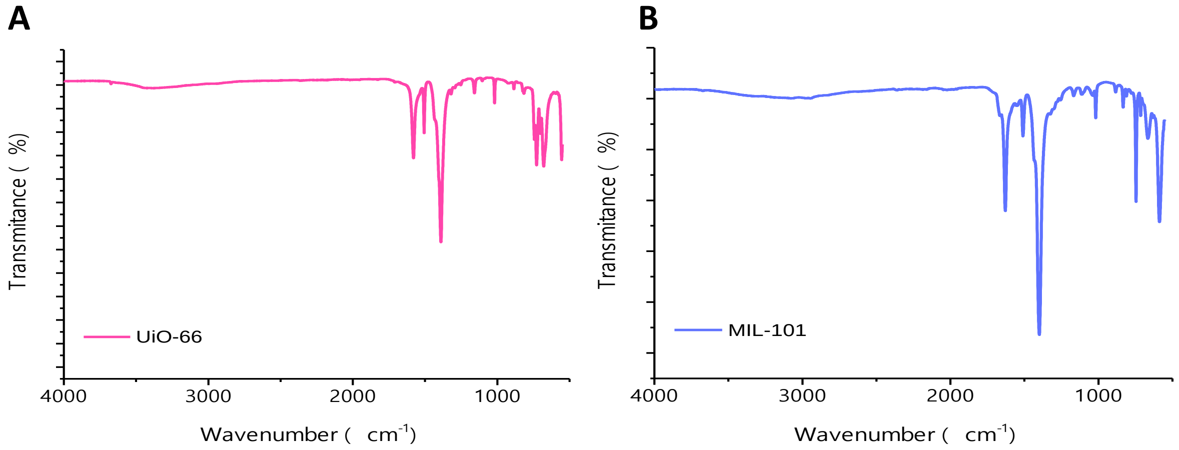

Figure S3: Solid state FT-IR spectra. (A) UiO-66 and (B) MIL-101(Cr).

SEM: The size and surface morphology of the synthesised materials was studied with SEM. The average diameter of the nanoparticles was calculated manually with the aid of ImageJ software. The diameter of an average of 200 nanoparticles was measured for each material and bins of 20 nm were used. For both materials, a characteristic octahedral shape was observed and the average particle dimeter for UiO-66 was 391±103 nm and for MIL-101(Cr) was 188±47 nm **(Figure S4)**.

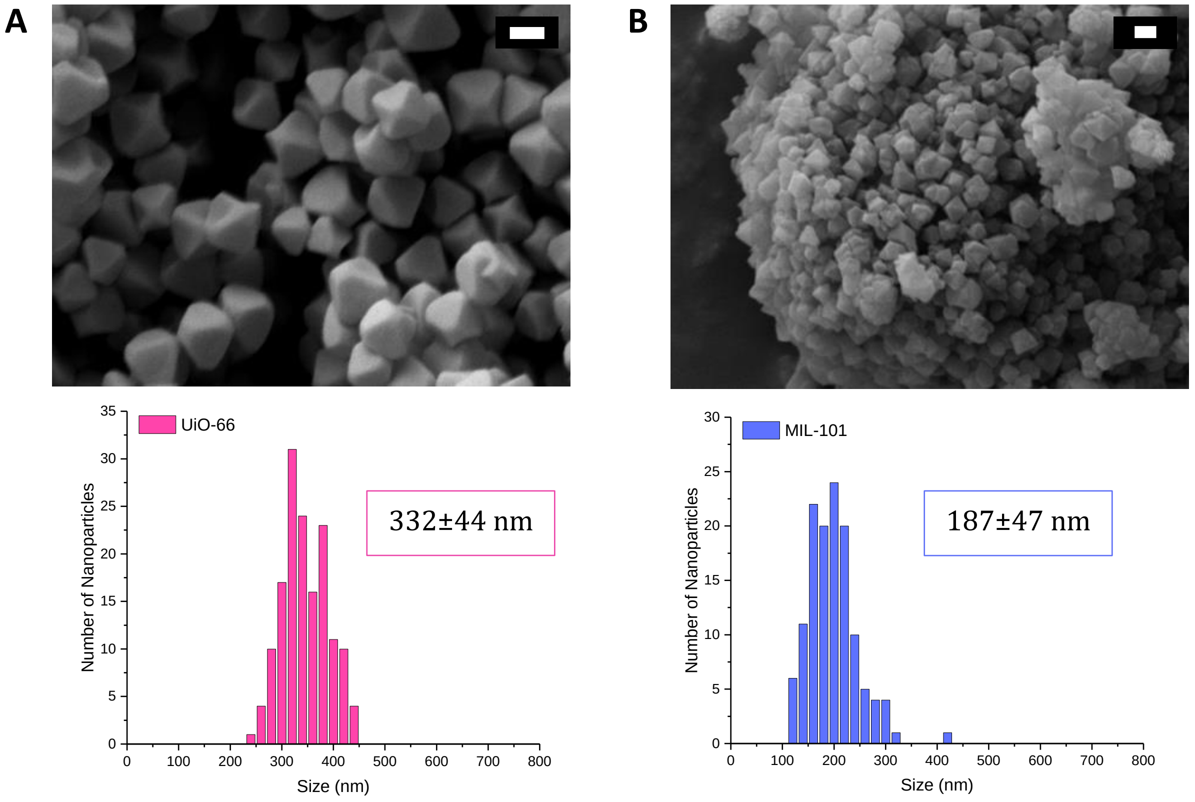

Figure S4: SEM images of (A) UiO-66 and (B) MIL-101(Cr). Scale bars represent 200 nm. Diameter size distribution graphs and average diameter size of (A) UiO-66 (PDI = 0.380) and (B) MIL-101(Cr) (PDI = 0.462) particles. Data represented as mean ± SD.

Dynamic Light Scattering (DLS): The hydrodynamic radius of the nanoparticles was also measured with DLS. In the case of UiO-66, minimal aggregation was observed between the three measurements. This was not significant however, as the intensity of the three consecutive measurements is not decreasing, as it is expected when serious aggregation occurs and the PDI value of the measurements was low (0.380). The average hydrodynamic radius of this material was 904 nm indicating that the colloidal suspension of the nanoparticles consists of small aggregates and not individual nanoparticles as their diameter was approximately 330 nm according to SEM. MIL-101(Cr) nanoparticles showed an interesting behaviour. Initially, during the first measurement, two populations were observed, one with a diameter of approximately 140 nm, which is very close to the diameter of individual nanoparticles according to SEM, and one of aggregates with diameter of approximately 955 nm. However, in the following measurements, the large hydrodynamic radius population disappeared. Moreover, the intensity of the population with small hydrodynamic radius increased, indicating that the nanoparticles were getting better suspended with potential large aggregate clusters breaking apart and reaching equilibrium at aggregates with lower hydrodynamic size of approximately 369 nm. No further aggregation of the material was observed which along with the low PDI of the measurement suggest that the material forms a stable colloidal suspension **(Figure S5)**.

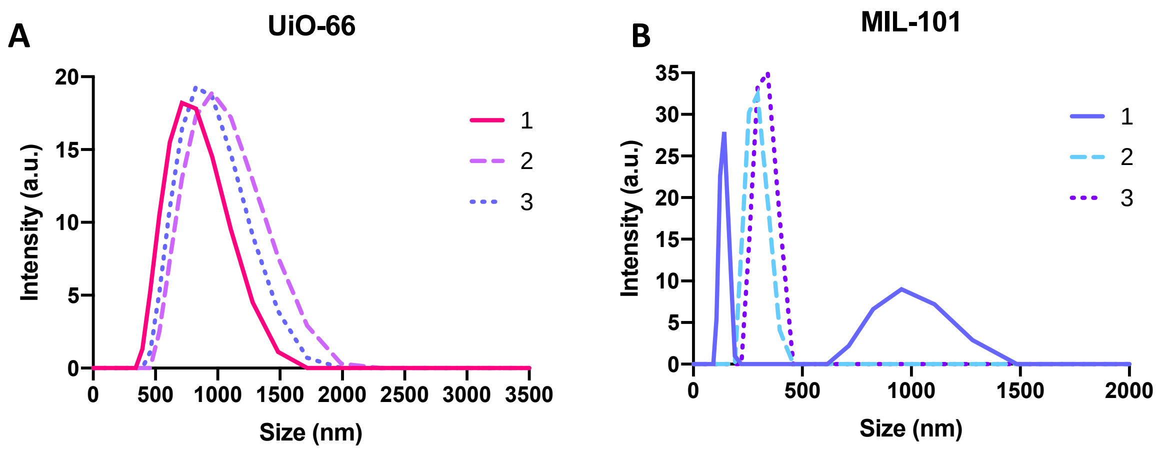

Figure S5: Three consecutive DLS measurements of MOF nanoparticles suspended in PBS 1X pH 7.4 spiked with 2% v/v FBS. (A) UiO-66 and (B) MIL-101(Cr).

Zeta potentials in deionized water and cell culture media were collected **(Figure S6)**, showing that while significant differences in the bare MOFs are evident, on suspension in media a protein corona formed to yield similar surface charge for both MOFs.[S6-S8]

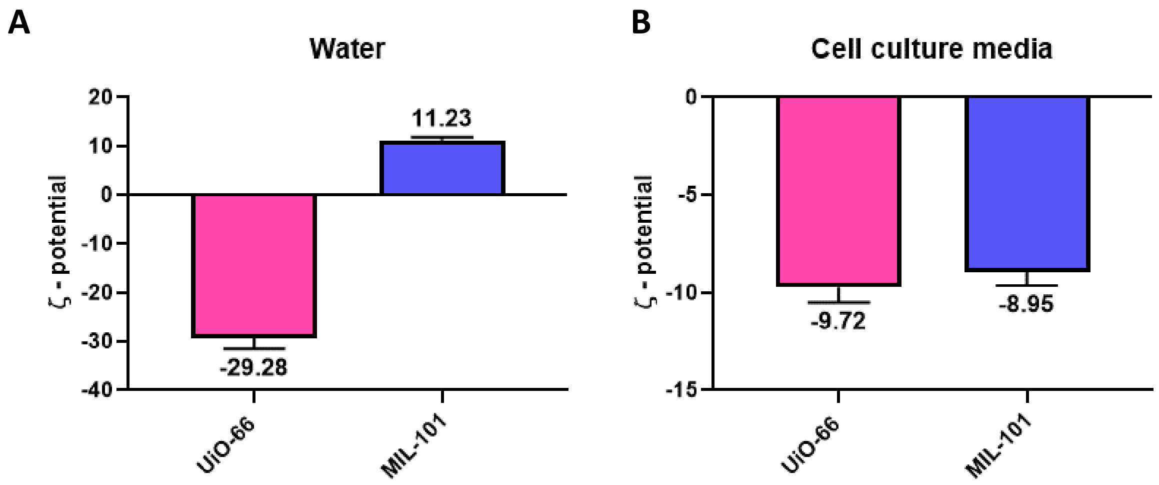

Figure S6: Surface ζ-potential measurements of MOF nanoparticles suspended in (A) water and (B) cell culture media.

In Vitro Biocompatibility Assays

Cell culture: All cell culture procedures were performed under sterile conditions. The cells that were used were cultured in Corning® T75 flasks at 37 ˚C, in a humidified incubator atmosphere maintained at 5% CO_2. For passaging, the cells were washed twice with PBS 1X and trypsinised at 37 °C for 7 minutes with Trypsin-EDTA (0.25%). Complete media in a 3:1 ratio was used to deactivate the effect of Trypsin, which was removed by centrifugation, and then the cells were seeded in new Corning® T75 flasks. The corresponding cell growth media used for each cell line as well as the seeding ratios can be found in **Table S1**.

Table S1: Cell growth media used for each cell line and their passaging seeding ratios.

Cell type	Media and supplements	Seeding ratio
HDF (primary)	DMEM with GlutaMAX™-I and 10% (v/v) heat inactivated FBS, 100 units mL^{-1} penicillin, 100 units mL^{-1} streptomycin, 1% Amphotericin B	1:3
HEK-293 (ECACC 85120602)	DMEM-high glucose and 10% (v/v) heat inactivated FBS, 2 mM L-glutamine, 100 units mL^{-1} penicillin, 100 units mL^{-1} streptomycin	1:10
MCF-7 (ECACC 86012803)		1:5
A2780ADR (ECACC 93112520)	RPMI-1640 and 10% (v/v) heat inactivated FBS, 2 mM L-glutamine, 100 units mL^{-1} penicillin, 100 units mL^{-1} streptomycin	1:5
HepG2 (ECACC 85011430)	MEM and 10% (v/v) heat inactivated FBS, 2 mM L-glutamine, 100 units mL^{-1} penicillin, 100 units mL^{-1} streptomycin, 1% (v/v) non-essential amino acids solution (100X)	1:5

RTCA: RTCA assays require the use of xCELLigence® RTCA instrument and E-plate VIEW 96 well plates. These plates are covered with gold microelectrodes at the bottom of the wells and an electrical signal generated by the instrument is allowed to pass through the electrodes. The presence of cells disrupts and delays the electrical signal. Hence, the disruption of the signal correlates very well with the number of cells present. By measuring this delay, we can estimate cell index, which reflects the number of cells present at a given point. Measuring cell index overtime allows us to observe cell proliferation, cell loss or cytostatic effects, as well as cell morphology. Control experiments showed that the MOFs themselves did not interfere with the impedance measurements (Figure S7). 100 µL complete media per well were added to the E-plate VIEW 96 and it was incubated at room temperature for 10 minutes. The plate was added to the RTCA instrument in the incubator (37˚C, 5% CO_2), to measure background impedance. The cells were counted and added to the plate at a concentration of 5×10^3 cells per well to a total volume of 200 µL per well. The plate was returned to the RTCA instrument and the cells were allowed to sediment for 30 minutes before the start of the measurement. After 24 hours of incubation, the media were carefully aspirated. The MOF nanoparticles were suspended in complete media and were sonicated for 5

minutes in the various concentrations. The MOF suspension treatment was added (200 µL per well) - 6 technical replicates for each concentration. Complete media were added in the untreated controls (200 µL per well) – 6 technical replicates. Typically, the addition of material and agitation results in a temporary small drop in cell index, which recovers quickly. The plate was returned to the RTCA instrument where cell growth was recorded for a further 72 hours. Overall, 3 biological replicates were performed. The RTCA traces for an indicative biological repeat using human dermal fibroblasts (HDFs) are shown in the main manuscript **(Figures 1A and 1B)** and for MCF-7 and HEK-293 cells in **Figure S8**. The slope analyses in **Figures 1C–1E** of the main manuscript combine all biological replicates.

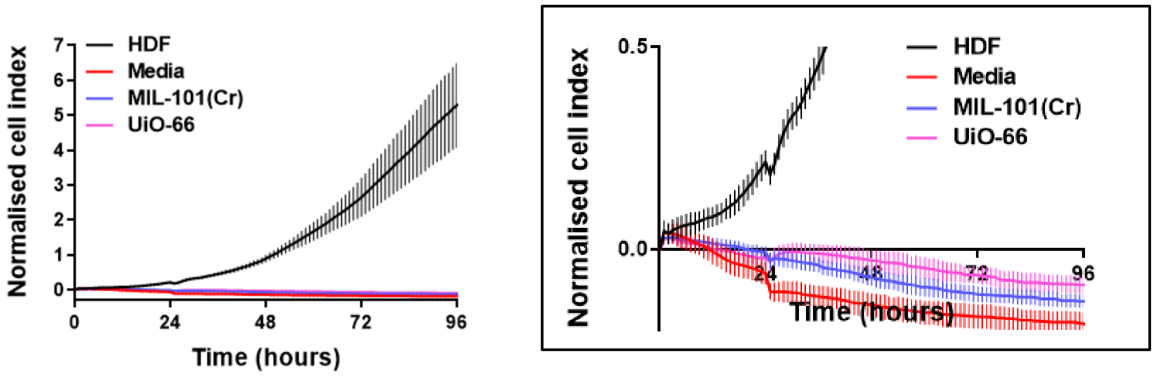

Figure S7: MOF addition does not increase cell index in the absence of HDF cells (right – inset).

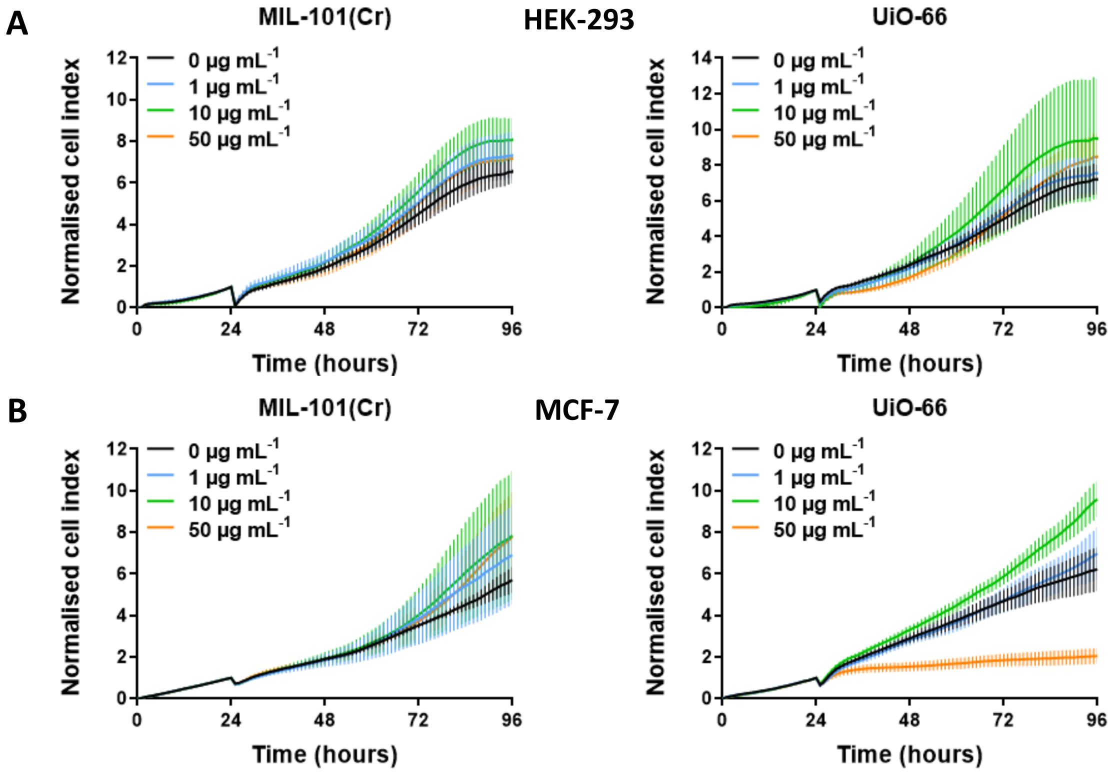

Figure S8: Biocompatibility assessment of MOFs. Real time cell analysis screening of MIL-101(Cr) and UiO-66 in (A) HEK-293 and (B) MCF-7 cells.

Alamar blue assays: Cells were seeded into a VWR treated 96 well plate at a density of 5×10^3 cells per well (200 µL media per well). The cells were incubated for 24 hours in a humidified incubator atmosphere maintained at 37 ˚C and 5% CO_2. The MOF nanoparticles were suspended in complete media and were sonicated for 5 minutes in the various concentrations. The media were aspirated from the cells and the MOF suspension treatment was added (200 µL per well) - 3 to 6 technical replicates for each concentration. Complete media were added in the untreated controls (200 µL per well) - 6 technical replicates. The cells were incubated for 24 or 72 hours in a humidified incubator atmosphere maintained at 5% CO_2. To measure the metabolic activity, 20 µL per well Alamar Blue™ Cell Viability Reagent were added and the cells were incubated for 3.5-5 hours (depending on the cell line) in a humidified incubator atmosphere with 5% CO_2. Then the fluorescence intensity was measured on the plate reader (Exc 557±10 nm, Ems 593±10 nm). Overall, 3 biological replicates were performed. The data are presented in **Figures 1F–1H** of the main manuscript.

Calcein Loading and Characterisation

Synthesis of Cal@MOFs: The MOF nanoparticles **(Table S2)** were suspended in a calcein solution in ethanol/water 1:1 *v/v* at a concentration of 1 $mgmL^{-1}$. The suspension was sonicated for 10 minutes and then was left stirring at room temperature for 96 hours. The nanoparticles were isolated with centrifugation (4500 rpm, 20 min) and were washed with ethanol until the supernatant was clear. For calcein loading determination the nanoparticles were degraded in PBS 1X pH 5.5 for 5 days and their corresponding calcein content was calculated with UV-Vis spectroscopy with the use of a calibration curve.

Table S2: MOF mass and calcein solution volume.

**MOF**	**Mass (g)**	**Calcein solution volume (mL)**
UiO-66	0.06709	30
MIL-101(Cr)	0.05177	25

The successful calcein staining was visually identified by the colour change of the materials from white to orange-yellow for UiO-66 and from green to brown for MIL-101(Cr) **(Figure S9)**.

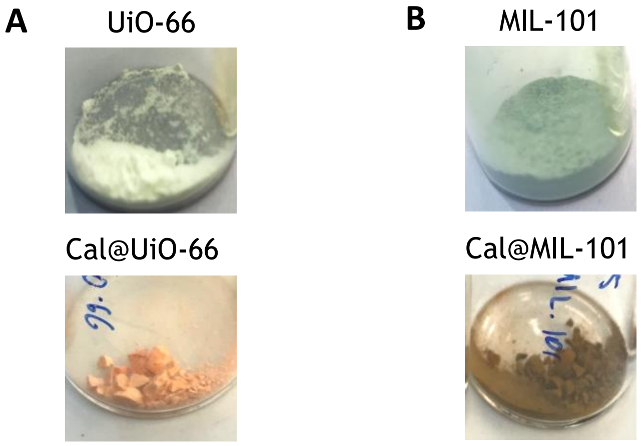

Figure S9: Colour change of the MOF nanoparticle powders after calcein staining. (A) UiO-66 and Cal@UiO-66. (B) MIL-101(Cr) and Cal@MIL-101(Cr).

The calcein loaded materials were highly crystalline and their structure was not altered as a very similar pattern to the as synthesised MOFs was obtained by PXRD **(Figure S10)**.

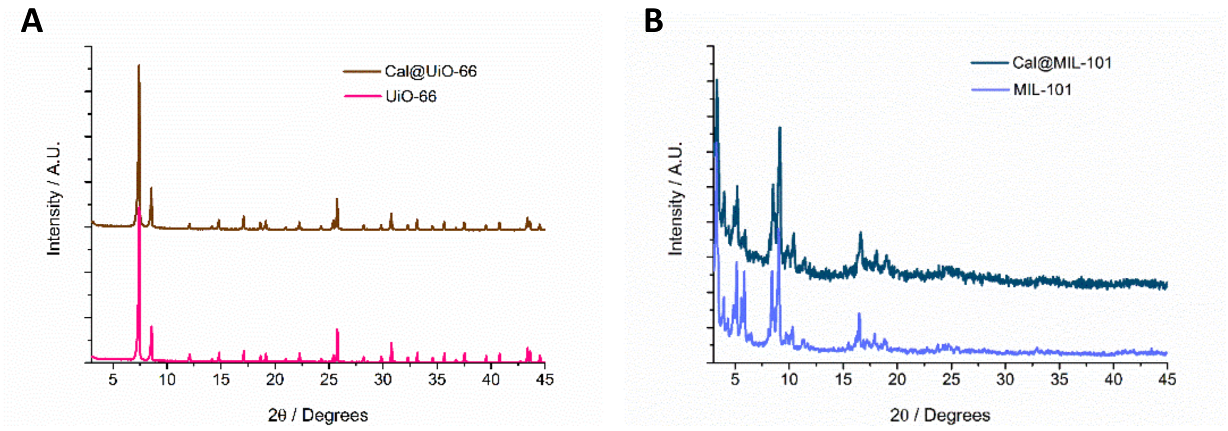

Figure S10: PXRD patterns. (A) UiO-66 and (B) MIL-101(Cr) nanoparticles before and after calcein staining.

The calcein stained materials had a very similar thermal stability as their "empty" precursors. This, along with the crystallinity of the materials, further confirms that the MOF structure was not compromised during calcein loading. The 100% mass of each material was normalised to 150°C to avoid taking solvent evaporation into account of organic mass loss. A small organic mass addition is observed indicating the successful incorporation of calcein to the MOF structure **(Figure S11)**.

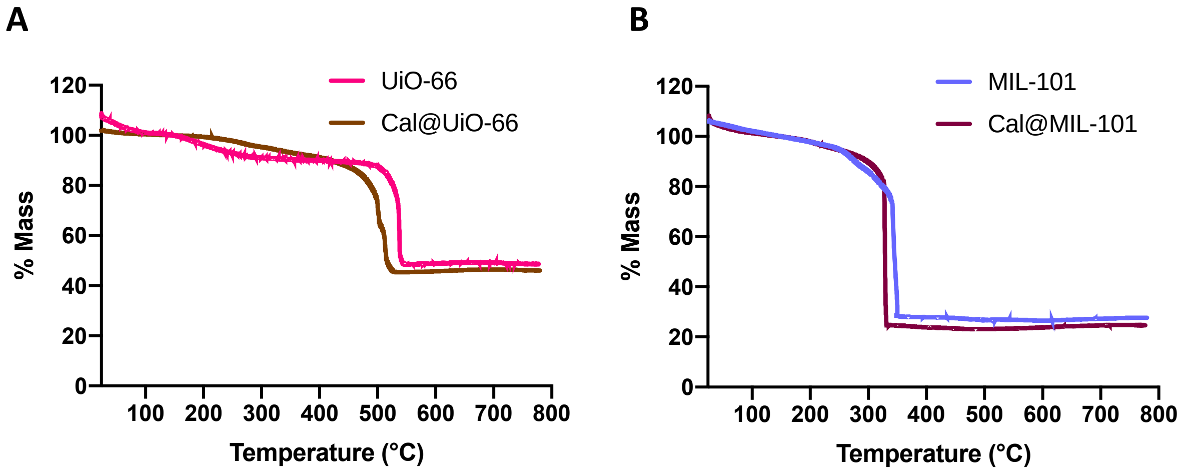

Figure S11: Thermogravimetric analysis profiles. (A) UiO-66 and (B) MIL-101(Cr) nanoparticles before and after calcein staining.

The calcein content of each MOF was calculated quantitatively by UV-Vis spectroscopy. The calcein stained nanoparticles were suspended in PBS 1X pH 5.5 in a concentration of 0.5 mgmL^{-1} with sonication for 10 minutes. The suspension was left stirring at room temperature until the solid residue was not stained orange (5-8 days). Then, the solid was removed with centrifugation and the calcein content of the nanoparticles was calculated based on the absorbance of the supernatant with the use of the calibration curve in **Figure S12** and using equation **S1**. The exact calcein content of each material can be found in **Table S3**.

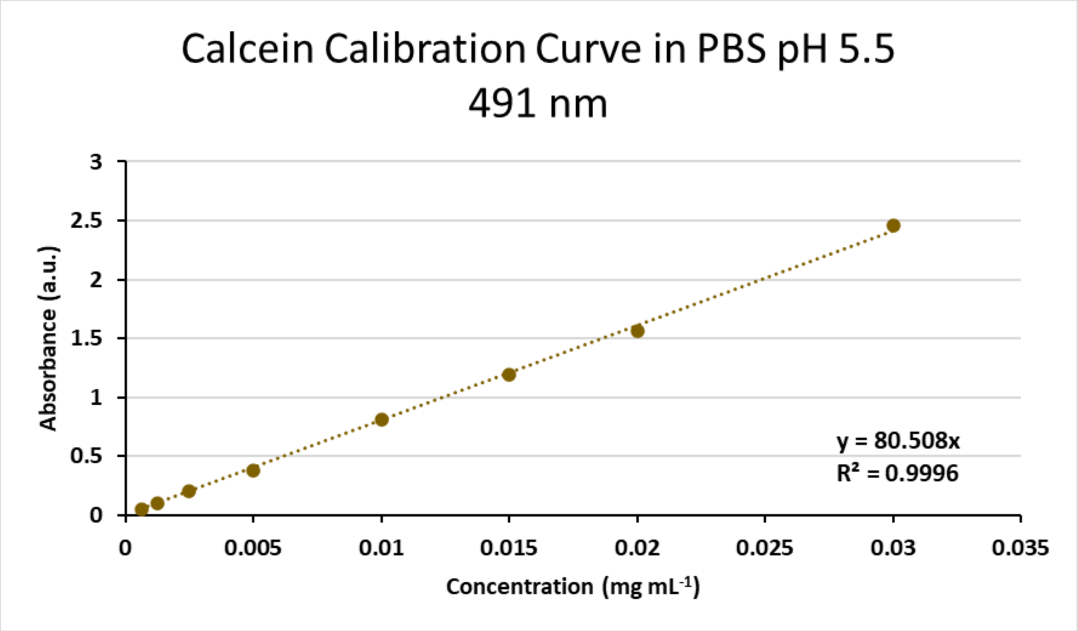

Figure S12: Calcein calibration curve in PBS 1X pH 5.5 based on absorbance at 491 nm.

$$\text{Calcein Content } (\%\, w/w) = \frac{\text{Loaded calcein mass}}{\text{MOF initial mass}} \times 100\% = \frac{\text{Abs@491 nm}/80.508}{0.5\ \text{mgmL}^{-1}} \times 100\% \quad \textbf{(S1)}$$

Table S3: Calcein content of each stained MOF.

MOF	Calcein content (*wt%*)
Cal@UiO-66	4.8
Cal@MIL-101(Cr)	6.7

Internalisation and Biocompatibility by Flow Cytometry

Flow cytometry: Cells were seeded into a VWR treated 96 well plate at a density of 5×10^3 cells per well (200 µL media per well). The cells were incubated for 24 hours in a humidified incubator atmosphere maintained at 37 ˚C and 5% CO_2. The MOF nanoparticles were suspended in complete media and were sonicated for 5 minutes in the various concentrations. The media were aspirated from the cells and the MOF suspension treatment was added (200 µL per well) – 3 to 6 technical replicates for each concentration. Complete media were added to the untreated controls (200 µL per well) – 6 technical replicates. The cells were incubated for 24 or 72 hours in a humidified incubator atmosphere maintained at 37 ˚C and 5% CO_2. The media were carefully aspirated and 50 µL per well Trypsin-EDTA (0.25%) were added. The plate was incubated (37 ˚C, 5% CO_2) for 5 minutes. 150 µL per well fresh media were added and the cells were resuspended by pipetting and transferred to a Corning® 96 round bottom well plate. Then, the cells were centrifuged at 1200 rpm for 10 minutes. The supernatant media was removed by flipping the plate over a piece of tissue paper. 200 µL of the Flow Buffer (PBS 1X, 2% FBS and 2 mM EDTA diluted in PBS 1X) per well were added with SYTOX™ AADvanced™ Dead Cell Stain at a concentration of 1 µL of 1 mM stain per mL of Flow Buffer. In the samples of untreated cells and FITC channel control used for the compensation of the instrument, the Flow Buffer solution added did not contain the SYTOX™ AADvanced™ Dead Cell Stain. For the sample used for the PeprCp-Cy5.5 channel control used for the compensation, 160 µL of the Flow Buffer containing the SYTOX™ AADvanced™ Dead Cell Stain and 40 µL of DMSO were added per well. Overall, 3 biological replicates were performed using the gating strategy in **Figure S13**.

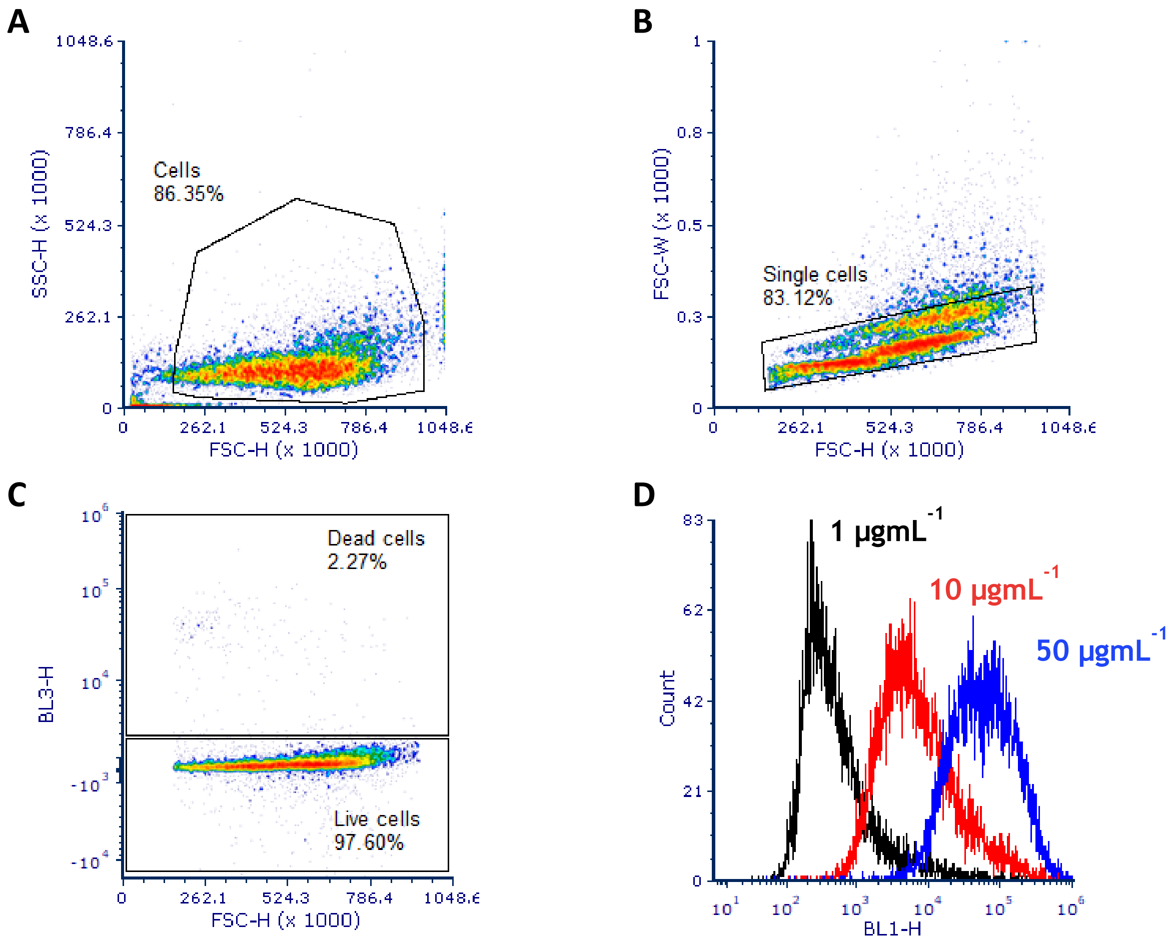

Figure S13: Gating strategy. (A) Gating of cells. (B) Gating of single cells. (C) Gating of live and dead cells. (D) Fluorescence intensity histogram of calcein stained live cells, with representative example of dose depended cytoplasmic fluorescence intensity increase after treatment with increasing concentrations of Cal@MIL-101(Cr).

The cell viability measurements were based on the absolute number of data points in the “live cells” gate instead of the percentage. This is due to the fact that the percentage represents “live cells” as a fraction of the total “cells” gate data points. In the case however where a treatment is causing a cytotoxic effect, the total “cells” data points are significantly less compared to the untreated control. This however does not necessarily mean that the percentage of “live cells” in the sample, as a fraction of the total “cells” number, is going to be significantly lower compared to the untreated control. Moreover, it needs to be noted that before running the experiment in the flow cytometer, a series of multiple preparation steps take place. During these steps, already dead cells are removed as they are no longer adherent to the bottom of the wells and this results in samples with significantly different total “cells” populations having similar percentages of “live cells”. Hence absolute numbers were used for the cell viability analysis.

Doxorubicin Loading and Characterisation

Synthesis of Dox@MOFs: 0.1 g doxorubicin was dissolved in 60 mL Tris buffered saline (sonication until complete dissolution). The MOF nanoparticles **(Table S4)** were added and the suspension was sonicated at room temperature for ten minutes and then was left stirring at room temperature for 5 days protected from light. The doxorubicin loaded nanoparticles **(Figure S14)** were isolated with centrifugation (4500 rpm for 20 minutes) and washed with Tris buffered saline until the supernatant was colourless. The supernatant loading solution and all the washings were collected and diluted up to 500 mL with Tris buffered saline. The drug loading capacity mass percentage (DLC% w/w) was calculated based on the concentration difference of the drug solution before and after the loading. This was done with UV-Vis spectroscopy with the use of a calibration curve. The drug loaded nanoparticles were dried *in vacuo* for 48 hours and then at 120 °C overnight before any characterisation or further experiments took place.

Table S4: MOF weight and doxorubicin solution volume.

MOF	Weight (g)	Doxorubicin solution volume (mL)
**UiO-66**	**0.0055**	**60**
**MIL-101(Cr)**	**0.0070**	**60**

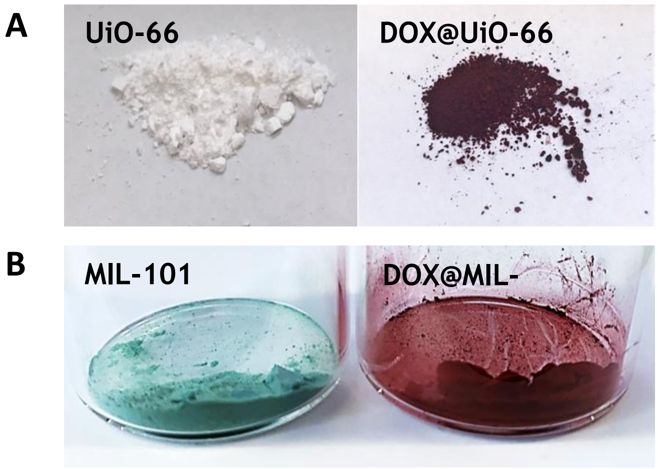

Figure S14: Colour change of the MOF nanoparticle powders after doxorubicin loading. (A) UiO-66 and Dox@UiO-66. (B) MIL-101(Cr) and Dox@MIL-101(Cr).

The mass-based drug loading capacity (DLC% wt) of each MOF was calculated based on the doxorubicin supernatant solution concentration difference before and after the loading. The doxorubicin concentration was calculated by UV-Vis spectroscopy with the use of the calibration curve in **Figure S15**.

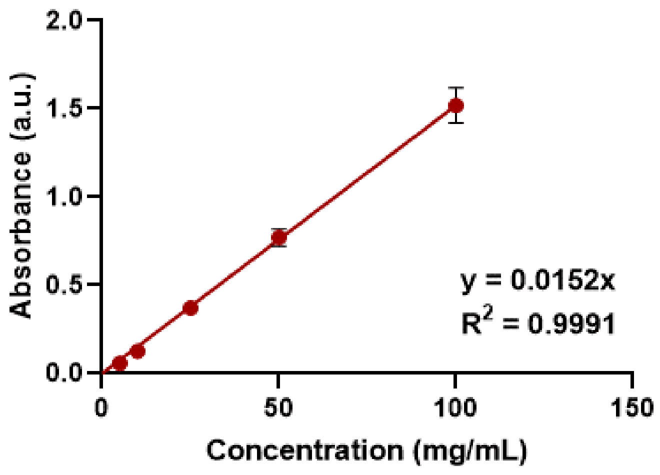

Figure S15: Doxorubicin calibration curve in Tris Buffered Saline pH 7.4 based on absorbance at 481 nm. Data represented as mean ± SD (n=3).

The wt% drug loading capacity of the materials was calculated based on grams of drug per grams of loaded material, using equation **S2**, and were 58% wt for UiO-66 and 10% wt for MIL-101(Cr).

$$\text{DLC\% wt} = \frac{\text{weight of loaded doxorubicin}}{\text{weight of loaded doxorubicin + MOF initial weight}} \times 100\% \quad \textbf{(S2)}$$

By TGA analysis, an increase of the organic content was obvious in both cases, indicative of successful drug loading **(Figure S16)**. For Dox@UiO-66, thermal degradation was observed at a lower temperature than the non-loaded MOF. This is a general trend that has been observed for surface functionalised MOFs.67,68 It is hypothesised that, due to the added organic surface functionality having a lower thermal stability, combustion is initiated at a lower temperature and that triggers earlier thermal decomposition of the material as a whole. For Dox@MIL-101(Cr) however, thermal decomposition began almost at the same stage as MIL-101(Cr), but the overall MOF degradation occurred at a slightly higher temperature.

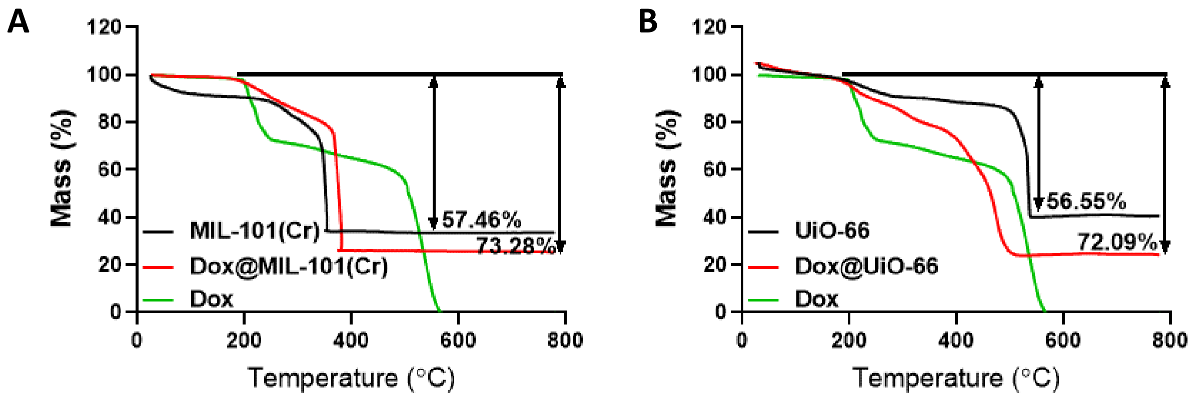

Figure S16: Thermogravimetric analysis profiles of **(G)** UiO-66 and **(H)** MIL-101(Cr), before and after Dox loading and compared to the profile of free Dox.

To computationally assess the different Dox uptakes in the two MOFs, we performed grand canonical Monte Carlo (GCMC) simulations of doxorubicin in UiO-66 and MIL-101(Cr) at 310 K with the multi-purpose code RASPA.[S9] We used an atomistic model of UiO-66 and MIL-101(Cr) for which the framework atoms were kept fixed at the crystallographic positions. We used the standard Lennard-Jones (LJ) 12-6 potential to model the Van der Waals interactions, using Lorentz-Berthelot mixing rules to define the interactions between the framework and adsorbate atoms, and a Coulomb potential to describe the electrostatic interactions. The parameters for the framework atoms **(Table S5)** were derived from the Universal Force Field[S10] and the Dreiding Force Field.[S11] Doxorubicin was modelled with the OPLS-AA forcefield **(**

Table S*6*),[S12] as a flexible model **(Figure S17)**, except for the rings that were kept rigid. The bonds, bends and torsion definitions used in RASPA for this molecule are described in

Table S7. EQeq was used to assign the partial charges of the framework.[S13] LJ interactions beyond 12.8 Å were neglected. The Ewald sum method was used to compute the electrostatic interactions in the system. Up to 200,000 Monte Carlo cycles were performed, the first 10% of which were used for equilibration, and the remaining steps were used to calculate the ensemble averages. Monte Carlo moves consisted of insertions, deletions, displacements, and rotations. In a cycle, *N* Monte Carlo moves are attempted, where *N* is defined as the maximum of 20 or the number of adsorbates in the system.

Table S5: Force field parameters for MIL-101 (Cr) and UiO-66.

Atom label	ε k_B^{-1} / K	σ / Å	Average charge (e^-)
**MIL-101(Cr)**			
Cr	7.54829	2.69319	1.529
C1	47.8562	3.47299	0.496
C2	47.8562	3.47299	-0.062
O1	48.1581	3.03315	-0.853
O2	48.1581	3.03315	-0.574
O3	48.1581	3.03315	-0.438
F	36.4834	3.0932	-0.547
H	7.64893	2.84642	0.108
**UiO-66**			
Zr	34.7221	2.78317	3.197
C	47.8562	3.47299	0.103
O	48.1581	3.03315	-0.844
H	7.64893	2.84642	0.0492

Table S6: Force field parameters for doxorubicin.

Atom label	Atom type	$\varepsilon\ k_B^{-1}$ / K	σ / Å	Charge (e^-)
C_ARO	CA	35.22	3.55	-0.115
C_ANI	CA	35.22	3.55	0.085
C_ANIt	CA	35.22	3.55	0.170
C_NAF	CA	35.22	3.55	0.0
C_KET	C_2	52.83	3.75	0.470
C_FEN	CA	35.22	3.55	0.150
C_PET	CT	33.21	3.5	0.170
C_CH2	CT	33.21	3.5	-0.120
C_CH3	CT	33.21	3.5	-0.180
C_CH3t	CT	33.21	3.5	-0.065
C_CHOH	CT	33.21	3.5	0.205
C_CH2OH	CT	33.21	3.5	-0.120
C_COH	CT	33.21	3.5	-0.265
C_PAM	CT	33.21	3.5	0.120
O_DET	OS	70.45	2.90	-0.300
O_ANI	OS	70.45	2.90	-0.285
O_KET	O_2	105.67	2.96	-0.470
O_FEN	OH	85.54	3.07	-0.585
O_OH	OH	85.54	3.12	-0.683
H_ARO	HA	15.09	2.42	0.115
H_FEN	HO	0.0	0.0	0.435
H_ET	HC	15.09	2.5	0.030
H_C	HC	15.09	2.5	0.060
H_OH	HO	0.0	0.0	0.418
H_CPA	HC	7.548	2.5	0.060
H_NPA	H	0.0	0.0	0.36
N_PAM	NT	85.54	3.3	-0.9

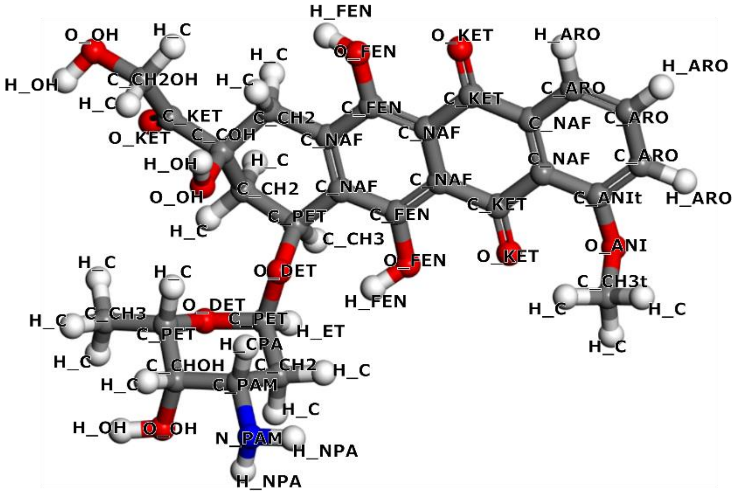

Figure S17: Indexing of the atoms in the doxorubicin model.

Table S7: Bonds, bends and torsion definitions for doxorubicin.

**Harmonic bond[1]**	**k_{eq} / K Å^{-2}**	**r_{eq} / Å**		
CT CT	269724.87	1.529		
CT HC	342188.27	1.090		
CT OH	322059.55	1.410		
OH HO	556558.68	0.945		
CT OS	322059.55	1.41		
CT C_2	319040.24	1.522		
CT NT	384458.59	1.448		
NT H	436793.27	1.010		
C_2 O_2	573668.57	1.229		
**Harmonic bend[2]**	**k_{eq} / K rad^{-2}**	**ϑ_{eq} / °**		
CT CT CT	58725.57	112.7		
CT CT HC	37741.35	110.7		
HC CT HC	33212.26	107.8		
CA CT HC	35225.26	109.5		
CT CT OH	50321.80	109.5		
CT OH HO	55353.99	108.5		
HC CT OH	35225.26	109.5		
CA OS CT	75482.71	111.0		
CT CT OS	50321.80	109.5		
HC CT OS	35225.26	109.5		
CT CT C_2	63405.47	110.10		
OH CT C_2	50321.80	109.5		
CT C_2 CT	70450.53	116.0		
CT C_2 O_2	80514.89	120.40		
HC CT C_2	35225.26	109.5		
CT CT NT	56561.64	109.47		
HC CT NT	35225.26	109.5		
CT NT H	35225.26	109.5		
H NT H	43880.64	106.4		

Torsion[3]	K_0 / kcal mol^{-1}	K_1 / kcal mol^{-1}	K_2 / kcal mol^{-1}	K_3 / kcal mol^{-1}
CT CT CT CT	1.299	-0.500	0.200	0.0
CT CT CT HC	0.0	0.0	0.300	0.0
HC CT CT HC	0.0	0.0	0.300	0.0
CT CT CT OH	1.710	-0.500	0.663	0.0
HC CT CT OH	0.0	0.0	0.468	0.0
CT CT OH HO	-0.356	-0.174	0.492	0.0
HC CT OH HO	0.0	0.0	0.450	0.0
CT CT OS CT	0.650	-0.250	0.670	0.0
HC CT OS CT	0.0	0.0	0.759	0.0
HC CT CT OS	0.0	0.0	0.468	0.0
OH CT CT OS	4.316	0.0	0.0	0.0
CA CA OS CT	0.0	2.998	0.0	0.0
CA OS CT HC	0.0	0.0	0.759	0.0
CA CA CT HC	0.0	0.0	0.0	0.0
CA CT CT HC	0.0	0.0	0.462	0.0
C_2 CT CT HC	0.0	0.0	-0.076	0.0
CT C_2 CT CT	1.453	-0.144	-0.774	0.0
CT C_2 CT HC	0.0	0.0	0.275	0.0
C_2 CT OH HO	-0.899	0.0	0.0	0.0
CT C_2 CT OH	1.710	-0.500	0.663	0.0
CT CT C_2 O_2	-0.277	1.227	-0.694	0.0
HC CT C_2 O_2	0.0	0.0	0.0	0.0
OH CT C_2 O_2	0.500	0.0	0.0	0.0
NT CT CT HC	-1.012	-0.708	0.468	0.0
CT CT NT H	-0.190	-0.417	0.418	0.0
HC CT NT H	0.0	0.0	0.400	0.0
NT CT CT OH	7.994	0.0	0.0	0.0

(1) Harmonic bond: $U = \frac{1}{2}k_{eq}\left(r_{ij} - r_{eq}\right)^2$

(2) Harmonic bend: $U = \frac{1}{2}k_{eq}\left(\theta_{ijk} - \theta_{eq}\right)^2$

(3) Torsion: $U = \frac{1}{2}K_0 + \frac{1}{2}K_1\left[1 + \cos\left(\varphi_{ijkl}\right)\right] + \frac{1}{2}K_2\left[1 - \cos\left(2\varphi_{ijkl}\right)\right] + \frac{1}{2}K_3\left[1 + \cos\left(3\varphi_{ijkl}\right)\right]$

The GCMC simulations carried out at 310 K and up to 150,000 Pa show zero uptake in UiO-66. **Figure S18** shows two snapshots of doxorubicin in UiO-66. These snapshots show that doxorubicin hardly fits inside the porosity of UiO-66, thus explaining the zero uptake.

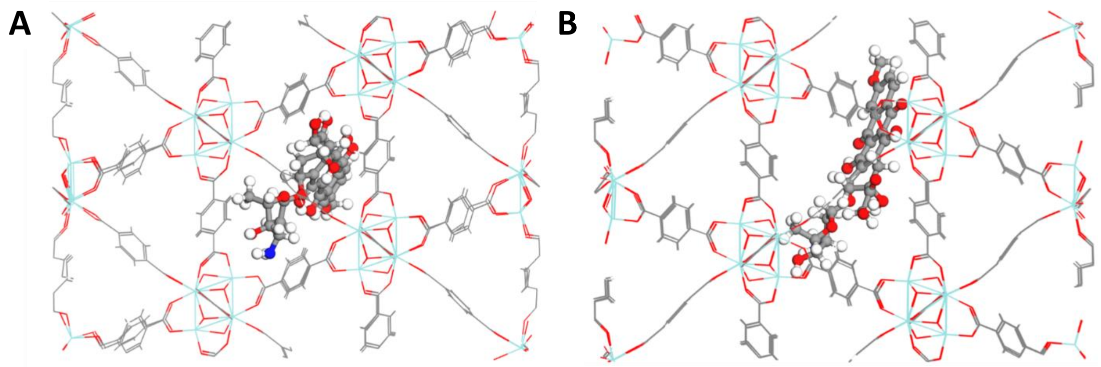

Figure S18: Snapshots of doxorubicin in UiO-66 from two perspectives.

The GCMC simulations on MIL-101(Cr) **(Figure S19)** lead to a saturation uptake of 2.1 mol kg^{-1} (or 1160 g kg^{-1}), which is ten times the amount adsorbed experimentally (110 g kg^{-1}). According to our calculation, the molecules adsorbed in the structure occupy 55.8% (1.046 cm^3 g^{-1}) of the pore volume (1.874 cm^3 g^{-1}). Therefore, given that the experimental adsorption is 9.5% of the maximum theoretical adsorption, this could occupy around 5.3% of the pore volume.

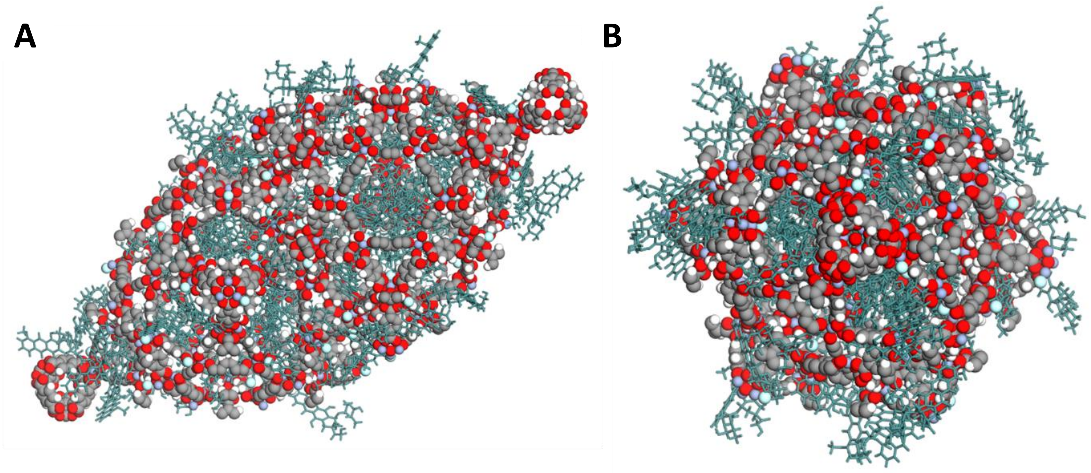

Figure S19: Snapshots of doxorubicin in the primitive cell of MIL-101(Cr) from two perspectives.

In these snapshots, the free volume within the structure seems completely full, which might be unexpected given that the 55.8% of the pore volume is occupied. However, the rigidity of the molecules and the difficulty to pack within the pores of the channel means some space is left between molecules, which explains the value of occupied pore volume.

For the drug delivery evaluation experiments the highest non-toxic concentration of each MOF was used. These were 1 μgmL^{-1} and 10 μgmL^{-1} for UiO-66 and MIL-101(Cr) respectively. In the case of the drug loaded materials (Dox@MOF), a normalisation of the weight to those previous concentrations was performed by taking into consideration their corresponding drug loading capacities, using equation **S3**.

$$\text{Dox@MOF}(\mu gmL^{-1}) = \frac{100 \text{ x } (\text{MOF } \mu gmL^{-1})}{(\text{MOF\% wt in Dox@MOF})} = \frac{100 \text{ x } (\text{MOF } \mu gmL^{-1})}{(100\text{-DLC\% wt})} \quad \textbf{(S3)}$$

This yielded concentrations of 2.38 μgmL^{-1} Dox@UiO-66 and 11.1 μgmL^{-1} Dox@MIL-101(Cr) being used. In a similar manner, the concentrations of equivalent amounts of free doxorubicin were calculated to be 1.38 μgmL^{-1} for Dox@UiO-66 and 1.1 μgmL^{-1} for Dox@MIL-101(Cr). A summary of the concentrations used for each material can be found on **Table S8**.

Table S8: Summary of concentrations used for each material for drug delivery experiments.

MOF	DLC (% wt)	MOF concentration (μgmL^{-1})	Dox@MOF concentration (μgmL^{-1})	Equivalent Dox concentration (μgmL^{-1})
UiO-66	58	1	2.38	1.38
MIL-101(Cr)	10	10	11.1	1.1

Drug release control: Fluorescence-based drug release experiments in cell culture media were carried out as a control. This was done to establish that any effect observed from the following experiments in this study corresponds to internalisation of the drug loaded nanoparticles and not to doxorubicin being released extracellularly and then being internalised by the cells.

A suspension of 100 μgmL^{-1} of DDS nanoparticles in complete Dulbecco's Modified Eagle cell culture Medium (DMEM) without phenol red was prepared with sonication for 15 minutes. A solution of an equivalent amount of free doxorubicin was also prepared for each system. The concentrations of doxorubicin used were 58 μgmL^{-1} for Dox@UiO-66 and 10 μgmL^{-1} for Dox@MIL-101(Cr). The fluorescence of the free drug was measured to establish the maximum fluorescence that could potentially be obtained from each DDS sample. Then, the fluorescence of the DDS suspension was measured over the time course of 8-9 days. In both cases there was a minimal initial burst release of about 20% and 10% of the drug for Dox@UiO-66 and Dox@MIL-101(Cr) respectively. This was to be expected as these are bare nanoparticles with no surface functionalisation adding protection against burst drug release. However, for both DDSs even after 8 days the drug release does not surpass 32% for Dox@UiO-66 and 20% for Dox@MIL-101(Cr) **(Figure S20)**. This like due to a combination of the stability of the MOF / drug coating or proteins present in the cell culture media (mainly albumin) forming a protective corona around the nanoparticles, inhibiting further drug release.[6-8]

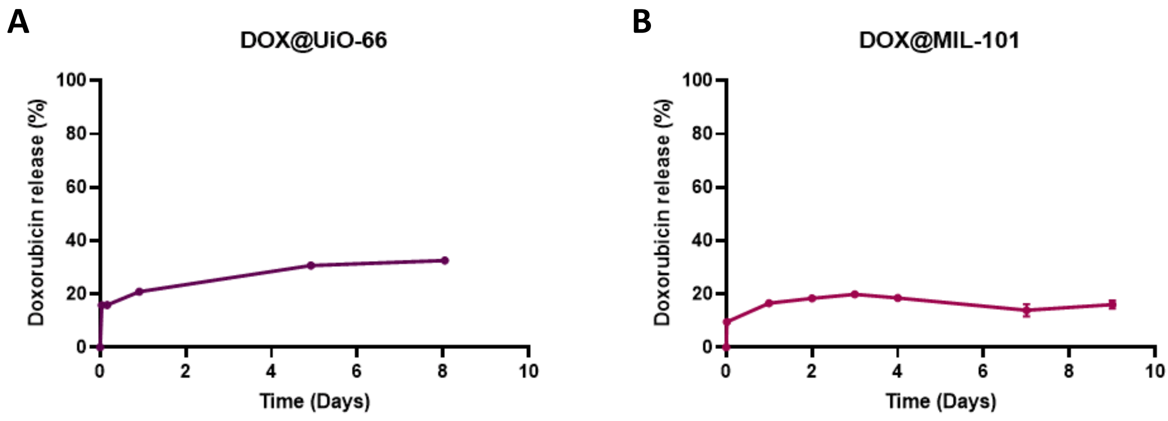

Figure S20: Fluorescence based doxorubicin release from (A) Dox@UiO-66 and (B) Dox@MIL-101(Cr) in complete cell culture media without phenol red. Data represented as mean ± SD (n=3).

Monitoring Drug Delivery by RTCA and Confocal Microscopy RTCA: 100 µL per well complete cell culture media were added to the E-plate VIEW 96 and it was incubated at room temperature for 10 minutes. The plate was added to the RTCA instrument in the incubator (37 ˚C, 5% CO_2), to measure background impedance. The cells were counted and added to the plate at a concentration of 5×10^3 cells per well to a total volume of 200 µL per well. The plate was returned to the RTCA instrument and the cells were allowed to sediment for 30 minutes before the start of the measurement. After 24 hours of incubation, the media were carefully aspirated. The MOF and Dox@MOF nanoparticles were suspended in complete media and were sonicated for 10 minutes in the appropriate concentrations. Free doxorubicin was dissolved in complete cell culture media at the appropriate concentration and was sonicated until complete dissolution. The MOF suspension or free Dox treatment was added (200 µL per well) - 6 technical replicates for each concentration. Complete media were added in the untreated controls (200 µL per well) - 6 technical replicates. The plate was returned to the RTCA instrument where cell growth was recorded for a further 72 hours. Overall, 3 biological replicates were performed.

Confocal microscopy: MCF-7 cells were seeded in a Nunc™ Lab-Tec™ II CC2™ Chamber Slide with removable walls system at a density of $2x10^5$ cells per well (400 µL media per well). The cells were incubated for 24 hours in a humidified incubator atmosphere maintained at 37 ˚C and 5% CO_2. The Dox@UiO-66 and Dox@MIL-101(Cr) nanoparticles were suspended in complete media and were sonicated for 10 minutes in the appropriate concentrations. Free doxorubicin was dissolved in complete cell culture media at the appropriate concentration and was sonicated until complete dissolution. The media were removed from the wells by turning the slide over a beaker containing Virkon disinfectant. Complete cell culture media were added to the controls (400 µL per well) and the Dox@MOF suspension or free drug solution treatment was added to the cells (400 µL per well) - 2 technical replicates. The cells were incubated in a humidified incubator atmosphere maintained at 37 ˚C and 5% CO_2 for the appropriate time (4, 6 and 12 hours for Dox@UiO-66 and 4 and 8 hours for Dox@MIL-101(Cr)). The media were removed from the wells by turning the slide over a beaker containing Virkon disinfectant. The cells were washed twice with PBS 1X with $CaCl_2$ and $MgCl_2$ (400 µL per well). Then, 400 µL per well CellMask™ Green plasma membrane stain diluted 1:500 in warm media were added to all the samples apart from the nucleus stain controls where 400 µL of complete media were added. The cells were incubated for 30 min in a humidified incubator atmosphere maintained at 37 ˚C and 5% CO_2. The stain was removed by flipping over Virkon disinfectant and the cells were washed twice with PBS 1X with $CaCl_2$ and $MgCl_2$ (400 µL per well). Subsequentially, 100% methanol was added to the wells (400 µL per well) and the cells were incubated at room temperature for 5 minutes. The methanol was removed by flipping over Virkon disinfectant and the cells were washed twice with PBS 1X with $CaCl_2$ and $MgCl_2$ (400 µL per well). After the final wash, the wall framework of the slide was removed and one drop of ProLong™ Glass Antifade Mountant with NucBlue™ nuclei stain was added over each sample and the slide was covered with a thin glass slide. The slide was let dry in the dark at room temperature overnight and then was stored at 4 ˚C. Overall, 3 biological replicates were performed.

The cells were imaged using a ZEISS LSM 780 Confocal Microscope and ZEN Black software. The NucBlue™ nuclear stain was observed using laser line 405, emitting at 410-504 nm. The CellMask™ Green membrane stain was observed using laser line 514, emitting at 517-553 nm. Doxorubicin was observed using laser line 488, emitting at 569-627 nm. All images were captured using a 20X Plan Apochromat objective and the gain was set at 700. During image analysis, doxorubicin fluorescence intensity was quantified by employing the macro Batch_quantify_v3392[S2] on Fiji. To quantify doxorubicin fluorescence intensity within the cells, the CellMask™ Green stain was used as

colocalization mask, while the NucBlue stain was used as a colocalization mask to quantify doxorubicin within the cell nucleus.

Supplemental References

S1. Macrae, C.F., et al. (2020). Mercury 4.0: from visualization to analysis, design and prediction. J. Appl. Cryst., *53*, 226-235.

S2. Johansson, J., et al. (2019). RAL GTPases Drive Intestinal Stem Cell Function and Regeneration through Internalization of WNT Signalosomes. Cell Stem cell, *24*, 592-607.e7.

S3. Cavka, J.H., Jakobsen, S., Olsbye, U., Guillou, N., Lamberti, C., Bordiga, S., and Lillerud, K.P. (2008). A New Zirconium Inorganic Building Brick Forming Metal Organic Frameworks with Exceptional Stability. J. Am. Chem. Soc., *130*, 13850-13851.

S4. Férey, G., Mellot-Draznieks, C., Serre, C., Millange, F., Dutour, J., Surblé, S., and Margiolaki, I. (2005). A Chromium Terephthalate-Based Solid with Unusually Large Pore Volumes and Surface Area. Science, *309*, 2040.

S5. Valenzano, L., Civalleri, B., Chavan, S., Bordiga, S., Nilsen, M.H., Jakobsen, S., Lillerud, K.P., and Lamberti, C. (2011). Disclosing the Complex Structure of UiO-66 Metal Organic Framework: A Synergic Combination of Experiment and Theory. Chem. Mater., *23*, 1700-1718.

S6. Nazarenus, M., et al. (2014). In vitro interaction of colloidal nanoparticles with mammalian cells: What have we learned thus far? Beilstein J. Nanotechnol., *5*, 1477-1490.

S7. del Pino, P., Pelaz, B., Zhang, Q., Maffre, P., Nienhaus, G.U., and Parak, W.J. (2014). Protein corona formation around nanoparticles – from the past to the future. Mater. Horiz., *1*, 301-313.

S8. Rojas, S., Carmona, F.J., Maldonado, C.R., Horcajada, P., Hidalgo, T., Serre, C., Navarro, J.A.R., and Barea, E. (2016). Nanoscaled Zinc Pyrazolate Metal–Organic Frameworks as Drug-Delivery Systems. Inorg. Chem., *55*, 2650-2663.

S9. Dubbeldam, D., Calero, S., Ellis, D.E., and Snurr, R.Q. (2016). RASPA: molecular simulation software for adsorption and diffusion in flexible nanoporous materials. Mol. Simulat., *42*, 81-101.

S10. Rappe, A.K., Casewit, C.J., Colwell, K.S., Goddard, W.A., and Skiff, W.M. (1992). UFF, a full periodic table force field for molecular mechanics and molecular dynamics simulations. J. Am. Chem. Soc., *114*, 10024-10035.

S11. Mayo, S.L., Olafson, B.D., and Goddard, W.A. (1990). DREIDING: a generic force field for molecular simulations. J. Phys. Chem., *94*, 8897-8909.

S12. Jorgensen, W.L., Maxwell, D.S., and Tirado-Rives, J. (1996). Development and Testing of the OPLS All-Atom Force Field on Conformational Energetics and Properties of Organic Liquids. J. Am. Chem. Soc., *118*, 11225-11236.

S13. Rappe, A.K. and Goddard, W.A. (1991). Charge equilibration for molecular dynamics simulations. J. Phys. Chem., *95*, 3358-3363.
