# Supplementary material for: Identifying Differing Intracellular Cargo Release Mechanisms by Monitoring In Vitro Drug Delivery from MOFs in Real Time
Source: Cell Rep Phys Sci. 2020 Nov 18;1(11):100254. doi: 10.1016/j.xcrp.2020.100254 (PMC7674849; doi:10.1016/j.xcrp.2020.100254)
Supplement: Document S2. Article plus Supplemental Information [file mmc2.pdf]

Cell Reports
Physical Science

CellPress
OPEN ACCESS

Article

Identifying Differing Intracellular Cargo Release Mechanisms by Monitoring *In Vitro* Drug Delivery from MOFs in Real Time

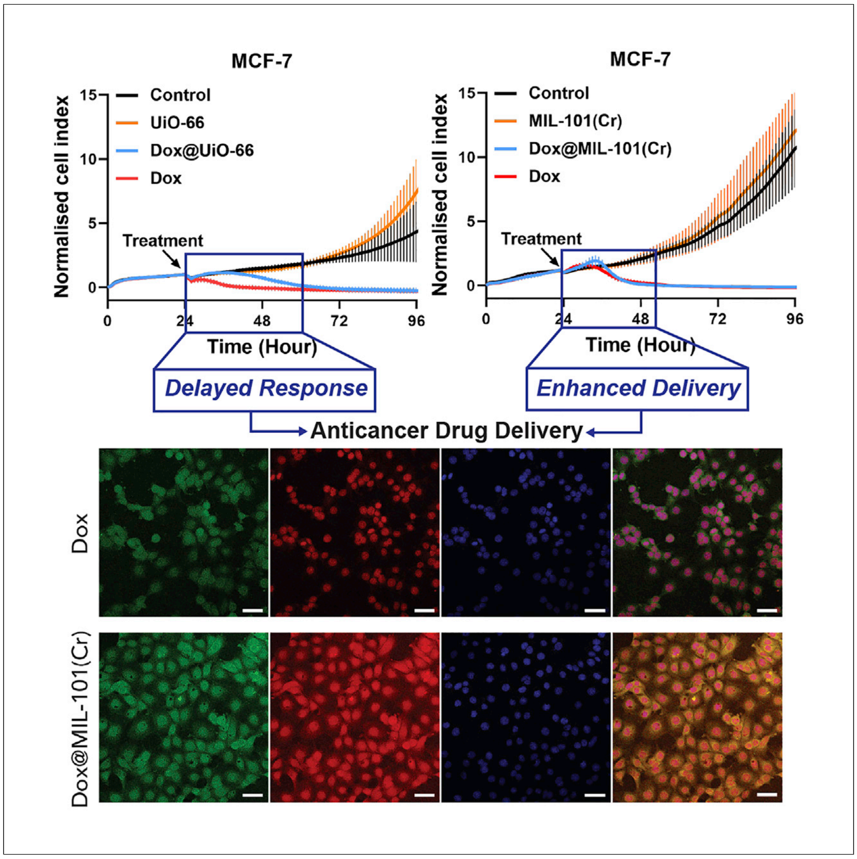

Markopoulou et al. demonstrate that real-time cell analysis can be used to assess biocompatibility and drug delivery from MOFs. Different time-dependent cytotoxicity profiles occur when drugs are pore loaded versus surface loaded, making tunable drug release possible.

Panagiota Markopoulou, Nikolaos Panagiotou, Aurelia Li, ..., David Fairen-Jimenez, Paul G. Shiels, Ross S. Forgan

paul.shiels@glasgow.ac.uk (P.G.S.)
ross.forgan@glasgow.ac.uk (R.S.F.)

HIGHLIGHTS

Comprehensive analysis confirms *in vitro* biocompatibility of UiO-66 and MIL-101(Cr)

Real-time observation of doxorubicin delivery from MOFs and subsequent cytotoxicity

Location of drug within MOF nanoparticles greatly affects release mechanisms

Tunable anticancer cytotoxicity profiles are possible by modifying drug location

Markopoulou et al., Cell Reports Physical Science *1*, 100254
November 18, 2020 © 2020 The Author(s).
https://doi.org/10.1016/j.xcrp.2020.100254

Cell Reports
Physical Science

CellPress
OPEN ACCESS

Article

Identifying Differing Intracellular Cargo Release Mechanisms by Monitoring *In Vitro* Drug Delivery from MOFs in Real Time

Panagiota Markopoulou,[1,4] Nikolaos Panagiotou,[1,3,4] Aurelia Li,[2] Rocio Bueno-Perez,[2] David Madden,[2] Sarah Buchanan,[3] David Fairen-Jimenez,[2] Paul G. Shiels,[3,5,*] and Ross S. Forgan[1,5,6,*]

SUMMARY

Metal-organic frameworks (MOFs) have been proposed as biocompatible candidates for the targeted intracellular delivery of chemotherapeutic payloads, but the site of drug loading and subsequent effect on intracellular release is often overlooked. Here, we analyze doxorubicin delivery to cancer cells by MIL-101(Cr) and UiO-66 in real time. Having experimentally and computationally verified that doxorubicin is pore loaded in MIL-101(Cr) and surface loaded on UiO-66, different time-dependent cytotoxicity profiles are observed by real-time cell analysis and confocal microscopy. The attenuated release of aggregated doxorubicin from the surface of Dox@UiO-66 results in a 12 to 16 h induction of cytotoxicity, while rapid release of pore-dispersed doxorubicin from Dox@MIL-101(Cr) leads to significantly higher intranuclear localization and rapid cell death. In verifying real-time cell analysis as a versatile tool to assess biocompatibility and drug delivery, we show that the localization of drugs in (or on) MOF nanoparticles controls delivery profiles and is key to understanding *in vitro* modes of action.

INTRODUCTION

Metal-organic frameworks (MOFs), coordination networks of metal ions or clusters linked by organic ligands into potentially porous materials, are being investigated increasingly as potential drug delivery systems (DDSs).[1–6] The ability to control particle size,[7–9] surface chemistry,[10] and internal porosity[11,12] has led to increasingly complex MOF-based materials. These have been designed to target specific cells[13] and organelles,[14] transport large specialized cargo such as oligonucleotides and proteins,[15–19] release these in response to specific stimuli,[20,21] and combine drug delivery with other techniques such as imaging[22–26] or photodynamic therapy.[27,28] Despite this diversification of material, the process of postsynthetic drug loading itself is often undercharacterized; cargo is often simply assumed to penetrate the porosity of the MOF despite potential competition from loading solvents. Additionally, binding cargo to the external surface of particles is already an established strategy for the delivery of larger molecules.[15] Typically, the cytotoxicity and efficacy of drug delivery are monitored *in vitro* by endpoint assays, in which a parameter, typically cell proliferation, is measured after incubation of the DDS with cells for a particular time, therefore only capturing a snapshot of data at a single time point. While these assays can be carried out over differing timescales, this consumes time and additional materials, and by necessity each time point is collected on a different cell population. We show here that real-time cell analysis (RTCA) can be applied not only to assess the real-time biocompatibility of MOFs[29,30] but also to monitor the cellular response to drug delivery. The potential of the technique is demonstrated by

[1]Joseph Black Building, College of Science and Engineering, School of Chemistry, University of Glasgow, Glasgow G12 8QQ, UK

[2]Adsorption & Advanced Materials Laboratory, Department of Chemical Engineering & Biotechnology, University of Cambridge, Cambridge CB3 0AS, UK

[3]Wolfson Wohl Cancer Research Centre, College of Medical, Veterinary, & Life Sciences, Institute of Cancer Sciences, University of Glasgow, Glasgow G61 1QH, UK

[4]These authors contributed equally

[5]Senior author

[6]Lead Contact

*Correspondence:
paul.shiels@glasgow.ac.uk (P.G.S.),
ross.forgan@glasgow.ac.uk (R.S.F.)

https://doi.org/10.1016/j.xcrp.2020.100254

This is an open access article under the CC BY license (http://creativecommons.org/licenses/by/4.0/).

discriminating between differing mechanisms of intracellular doxorubicin (Dox) release from two benchmark MOF DDSs, and in doing so, uncovering a potential candidate for controlled, enhanced Dox delivery over a number of hours.

Dox is widely used in studies involving novel DDSs, as it has strong absorption ($\varepsilon \sim 10^4$ $Lmol^{-1}cm^{-1}$ at λ_{max} = 480 nm) and emission (λ_{em} ~600 nm, Φ ~10% over a range of excitation wavelengths),[31] allowing spectroscopic assessment of loading, release, and intracellular accumulation, and it displays significant anticancer cytotoxicity. It is widely used in the clinic, with liposomal formulations such as Doxil (recognized as the first example of nanoparticulate drug delivery)[32] and Myocet[33] used against breast cancer, for example. There are a significant number of studies describing the delivery of Dox by a diverse range of MOFs—it is likely the most commonly used drug molecule in this area—and it has allowed exemplification of strategies such as targeted tumor uptake,[34,35] stimuli-responsive release,[36,37] multimodal treatments,[38,39] and theranostics.[40,41] A large number of these reports focus on the delivery of Dox from nanoparticles and composites of ZIF-8,[34,38,39,42–50] in which tetrahedral Zn^{2+} centers connect 2-methylimidazolate linkers into a **sod** net,[51] and UiO-66,[34,35,52–54] in which $Zr_6O_4(OH)_4(RCO_2)_{12}$ secondary building units (SBUs) connect 1,4-benzenedicarboxylate (BDC) linkers into a **fcu** net.[55] In both cases, the small pore apertures—3.4 Å for ZIF-8 (up to 12.0 Å when flexibility[56,57] is taken into account) and 6.0 Å for UiO-66—seemingly preclude penetration of the Dox molecule, which has a maximum diameter of 15.4 Å,[58] into the porosity of the MOF. While some reports describe *in situ* encapsulation of Dox during the synthesis of these smaller-pore MOFs,[38,39,42,43] the size disparity suggests it would be bound on external particle surfaces if loaded postsynthetically, which should modify release mechanisms. The localization of Dox on MOF nanoparticle surfaces would also have a significant impact on external surface modifications, which are often used to induce targeting or stimuli-responsive release mechanisms, but this is rarely discussed.[35,49,50]

In this study, we probe Dox (in the form of doxorubicin hydrochloride) loading and release using UiO-66, which has been studied intensely for drug delivery,[59] as a small-pore MOF (ZIF-8 has been shown to have poor stability to pH < 7[60] and in certain biological buffers[61,62]) and MIL-101(Cr), in which BDC linkers connect trimeric $Cr_3O(RCO_2)_6(H_2O)_2X$ (X = a monoanion) SBUs into the **mtn** topology with pore apertures of 12.0, 14.7, and 16.0 Å in diameter,[63] as a large-pore MOF. There are a small number of studies into Dox uptake and delivery by MIL-101(Fe) and derivatives,[34,64–66] one of which is clearly indicative of pore loading,[67] but none into MIL-101(Cr). While there is a stigma regarding cytotoxicity associated with Cr, it is thought to be at the very least a nutritionally or pharmacologically beneficial factor or even an essential nutrient,[68] and a small number of *in vitro*[30,69,70] and *in vivo*[71] studies on MIL-101(Cr) suggest good biocompatibility. Combined with its renowned chemical stability, which will preserve pore structure, it is an excellent candidate for these mechanistic studies. Using these two MOFs as DDSs, we show that RTCA can discriminate between the differing release mechanisms that result from contrasting Dox loading locations (external surface versus internal porosity), revealing Dox@MIL-101(Cr) as a potential controlled release chemotherapeutic and highlighting the need for multiple complementary *in vitro* experiments rather than endpoint assays in the development of novel DDSs.

RESULTS AND DISCUSSION

MIL-101(Cr) and UiO-66 Nanoparticles' Biocompatibility Assessment

UiO-66 and MIL-101(Cr) were obtained (Supplemental Experimental Procedures and Figures S1–S6) as crystalline nanoparticles after minor alterations to established

CellPress
OPEN ACCESS

literature procedures.[63,72] The MOFs were highly crystalline and thermally stable (Figure S1), while size and external surface morphology characterization indicated that both MOFs were nanoparticulate (Figure S4). After synthesis and characterization, the biocompatibility of the bare MOF nanomaterials was studied *in vitro* using three established experimental cell cultures comprising healthy primary human dermal fibroblasts (HDFs), immortalized human embryonic kidney (HEK-293) cells, and human breast adenocarcinoma (MCF-7) cells (Table S1 and Figures S7 and S8).

Initially, RTCA was used to investigate MOF biocompatibility using the xCELLigence RTCA instrument and E-Plate VIEW 96-well plates. These 96-well electronic microtiter plates are covered with gold microelectrodes at the bottom of the wells, which allow an electrical signal, generated by the instrument, to pass through the electrodes. The presence of adherent cells at the bottom of the wells impedes the electrical signal; the higher the electrical impedance, the more cells are present. Noninvasive electrical impedance monitoring allows quantification of an instrument-generated cell index parameter, which reflects the surface area of the bottom of the well that is covered by cells and essentially the relative number of cells present. The cells were seeded in E-Plate VIEW 96-well plates, incubated at 37°C for 24 h, and the MOF nanoparticles were then administered as a suspension in complete cell culture media in a series of increasing doses (1, 10, and 50 $\mu g\ mL^{-1}$). The cells were incubated in the presence of MOFs at 37°C, and cell proliferation, cell loss, cytostatic effects, morphology changes, and attachment quality following MOF addition were measured label-free and in real time for 3 days. Control experiments showed that the MOFs themselves did not interfere with the impedance measurements (Figure S7).

Examples of the real-time data collected for HDF growth are given in Figure 1A (MIL-101(Cr)) and Figure 1B (UiO-66). Rather than compare individual cell growth plots, the RTCA data from these experiments and analogs using MCF-7 and HEK-293 cells (see Supplemental Information for RTCA traces) are interpreted using slope analysis from 24 to 96 h, to quantify cell proliferative capacity following MOF administration as a biocompatibility measure (Figures 1C–1E). Following MIL-101(Cr) administration, cell growth was found to be significantly inhibited only when the highest dose was administered in HDFs, and not at all in HEK-293 or MCF-7 cells. The cell growth rate matched that of the controls for all of the concentrations tested in HEK-293 and MCF-7 cells, suggesting that MIL-101(Cr) is not cytotoxic against these cell lines. For the HDF cells, MIL-101(Cr) did not affect proliferation when 1 and 10 $\mu g\ mL^{-1}$ were administered, and cell growth was not significantly different from the untreated controls (Figure 1C). However, a MIL-101(Cr) concentration of 50 $\mu g\ mL^{-1}$ had a slightly negative effect on the cell index.

Treatment of HDFs with UiO-66 nanoparticles did not cause any adverse effect at 1 $\mu g\ mL^{-1}$, but treatment with higher doses (10 and 50 $\mu g\ mL^{-1}$) resulted in a statistically significant decrease in cell proliferative capacity. Interestingly, in HEK-293 cells, the cell index was found to be increased after 1 and 10 $\mu g\ mL^{-1}$ MIL-101(Cr) were administered and a significant increase in cell growth rate was calculated (Figure 1D). This was also observed in MCF-7 cells, following the addition of 1 $\mu g\ mL^{-1}$ UiO-66 (Figure 1E). We believe this observation, however, is not due to the increased cell number, but is the result of changes in cell morphology, as we elucidate later in this study through flow cytometry experiments. More specifically, the size of these cells increases when nanoparticles are internalized and thus impede the electrical signal at a higher rate.

To compare RTCA with an endpoint assay and to study the effect of MOF administration on the metabolic activity of the three different cell lines, the Alamar blue

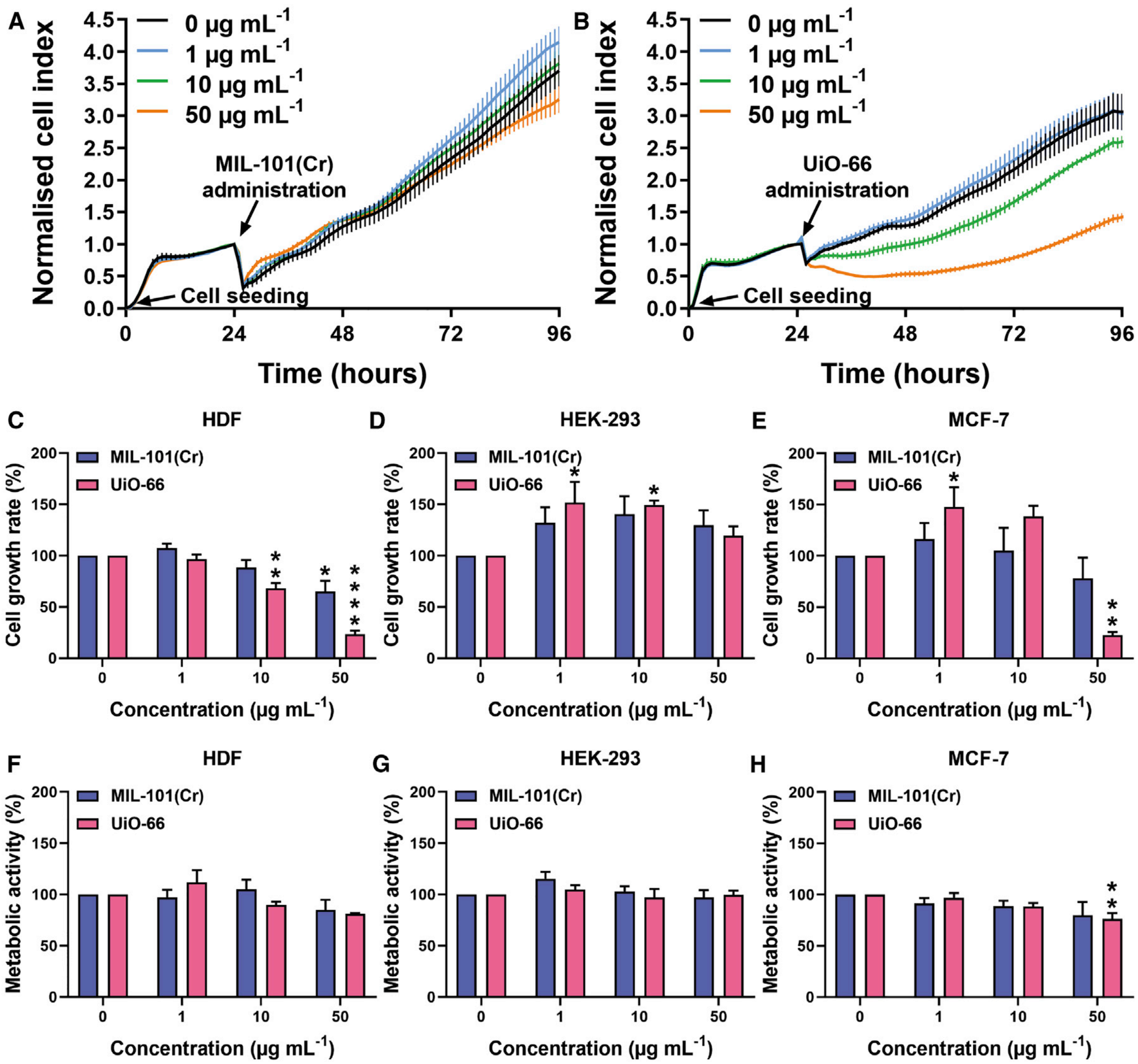

Figure 1. Biocompatibility Assessment of MOFs

(A and B) Real-time cell analysis (RTCA) screening of (A) MIL-101(Cr) and (B) UiO-66 in HDFs. Cells were seeded at 0 h and MOFs were administered at 24 h. Cell growth was then monitored until 96 h. The data are presented as means ± SDs (n = 6).

(C–E) The cell proliferative capacity was assessed by slope analysis of RTCA data from 24 to 96 h for (C) HDF, (D) HEK-293, and (E) MCF-7 cells. The data are presented as means ± SEMs. One-way ANOVA with Dunnett's test (n = 3).

(F–H) Direct comparison with cell metabolic activity, evaluated with Alamar blue assay, for (F) HDF, (G) HEK-293, and (H) MCF-7 cells, 72 h post-MOFs administration. Data are presented as means ± SEMs; One-way ANOVA with Dunnett's test (n = 3).

*p ≤ 0.05, **p ≤ 0.01, ***p ≤ 0.001, and ****p ≤ 0.0001.

assay was used (Figures 1F–1H). This assay is very similar to the widely used MTT/MTS assays, as it is based on the NAD(P)H-dependent metabolism of a reagent to generate a spectroscopic reporter; however, it is fluorescence based, whereas MTT/MTS assays are colorimetric, therefore precluding the possibility of interference by absorption from sedimented MOF particles. MIL-101(Cr) did not inhibit the metabolic activity of any of the 3 cell lines tested, while UiO-66 showed a statistically significant reduction in metabolic activity in MCF-7 cells when the highest concentration (50 μg mL^{-1}) was administered (Figure 1H). In general, there was good correlation in biocompatibility between RTCA and the Alamar blue measurements for HEK-293 (Figures 1D and 1G) and MCF-7 cells (Figures 1E and 1H). In HDFs,

CellPress
OPEN ACCESS

however, the RTCA and subsequent slope analysis (Figure 1C) demonstrated some negative MOF-associated effects on cell growth that the Alamar blue assay (Figure 1F) failed to report. The metabolic assay did not show any significant adverse effects following MOF treatment, whereas the more sensitive RTCA indicated significant disturbances in cell growth. Overall, the results are broadly comparable, showing that MIL-101(Cr) seems to be better tolerated across the cell lines than UiO-66 under these conditions, and confirming that doses of 1 $\mu g\ mL^{-1}$ (UiO-66) and 10 $\mu g\ mL^{-1}$ (MIL-101(Cr)) are suitable for further drug delivery experiments. They do, however, highlight the power and sensitivity of RTCA in assessing biocompatibility, validating its efficacy, and confirm that multiple techniques should be used to assess *in vitro* cytotoxicity of nanomaterials rather than standard, single-point assays.

MOFs Are Internalized by Cells and Enhance the Delivery of Calcein

The internalization of MOFs by HEK-293 and MCF-7 cells was investigated by flow cytometry, 24 and 72 h post-administration, using samples that had been calcein (Cal) stained using established protocols[73,74] (Figures S9–S12; Tables S2 and S3). Both Cal@UiO-66 (4.8 wt% calcein loading) and Cal@MIL-101(Cr) (6.7 wt% calcein loading) were internalized by both cell types and with dose dependence, which is indicative of successful MOF internalization (Figure S13). In HEK-293 cells, higher levels of internalization were observed after 24 h of treatment compared to 72 h (Figures 2A and 2B), indicating that either the MOFs or their calcein cargo are being externalized over time, an effect that suggests the potential of the low bioaccumulation of MOFs in healthy tissue. In MCF-7 cells, higher internalization levels were observed after 72 h of treatment. This was attributed to the enhanced metabolism of cancer cells, an observation that makes MOFs very promising candidates for use in drug delivery, as higher cargo (i.e., drug) concentrations can be internalized over time (Figures 2C and 2D). In general, both calcein-loaded MOFs resulted in higher cargo internalization compared to the free calcein molecule; improved cargo internalization is essential in drug delivery, as lower drug doses can be used, minimizing unwanted side effects and off-target toxicity. Cal@MIL-101(Cr) outperformed Cal@UiO-66 at concentrations >10 $\mu g\ mL^{-1}$, while their calcein delivery efficiency was similar for 1 $\mu g\ mL^{-1}$, and the enhanced uptake compared to free calcein was more pronounced after 24 h, suggestive of rapid nanoparticle endocytosis by different mechanisms to the free molecule.

As calcein is not toxic to the cells (confirmed by the flow cytometry experiments with free calcein and previous work[73,74]) cell viability could also be determined using the flow cytometry data, through live cell number measurements. These values correlate well with the RTCA data and support the hypothesis that the increase in cell index measured by the technique was due to an increase in cell size, rather than an increase in the number of cells (Figures 2E and 2F). Following UiO-66 administration of 1 and 10 $\mu g\ mL^{-1}$ in both HEK-293 and MCF-7 cells, the number of cells remained the same as that of the controls, indicating that there was no increase in proliferation rate, despite the apparent increase in growth rate by RTCA. In both cell lines, 50 $\mu g\ mL^{-1}$ UiO-66 caused a significant reduction in cell viability and increase in cell loss from as early as 24 h post-treatment. The cell cultures did not recover fully, and this effect was still observable after 72 h (Figures 2E and 2F). MIL-101(Cr), however, did not significantly affect cell viability. For both cell lines and for all of the concentrations that were tested, cell viability matched that of the controls at both time points of investigation. An increase in cell size as a result of MOF internalization was again observed with RTCA in these two cell types following MIL-101(Cr) administration, but this did not produce statistically significant changes in the overall cell

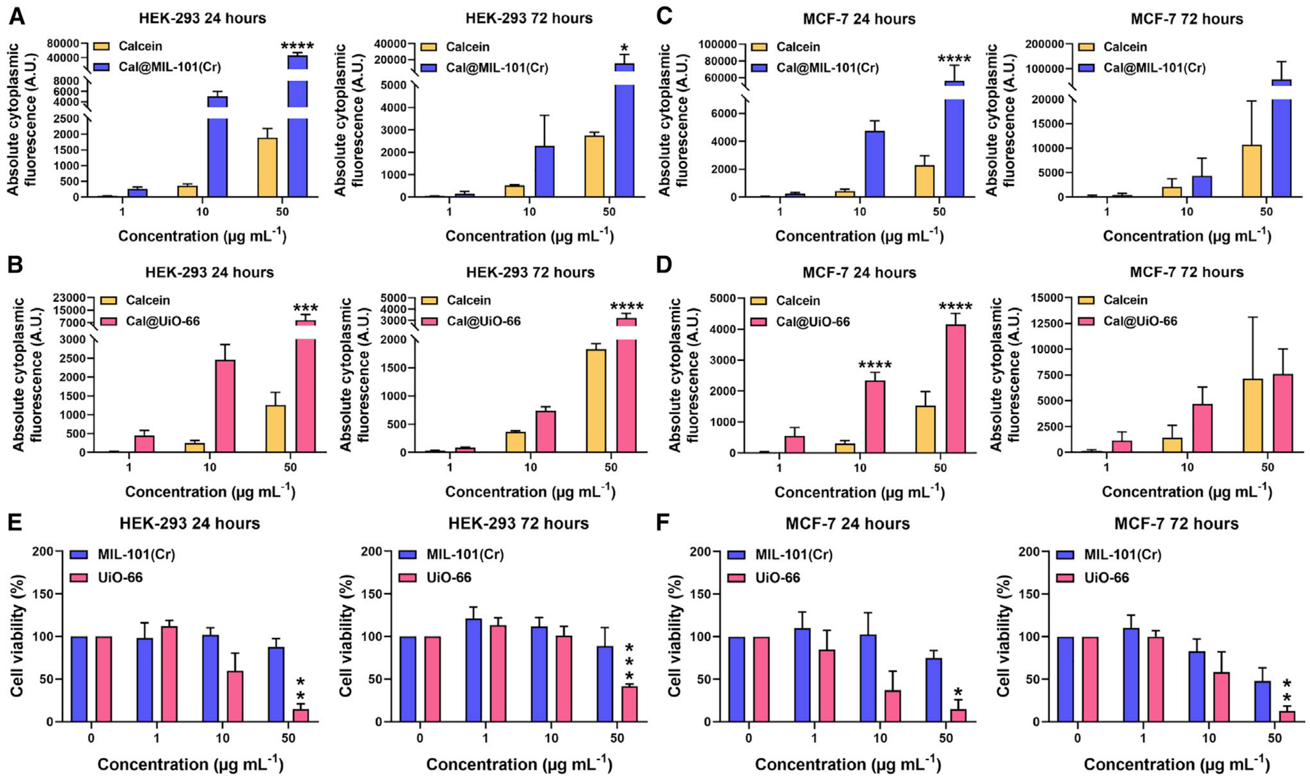

Figure 2. Cal@MIL-101(Cr) and Cal@UiO-66 Are Internalized by Cells and Enhance the Delivery of Calcein

(A–D) Flow cytometry of free calcein versus Cal@MOF in HEK-293 cells, for (A) Cal@MIL-101(Cr) and (B) Cal@UiO-66, and in MCF-7 cells, for (C) Cal@MIL-101(Cr) and (D) Cal@UiO-66, measured 24 and 72 h post-administration. The concentration that was used for free calcein corresponds to the amount of calcein loaded in Cal@MIL-101(Cr) and Cal@UiO-66, respectively. The data are presented as means ± SEMs; 1-way ANOVA with Sidak's test (n = 3).

(E and F) Cell viability assessed with flow cytometry in (E) HEK-293 and (F) MCF-7 cells, 24 and 72 h post-MOF administration with Cal@MIL-101(Cr) and Cal@UiO-66. The data are presented as means ± SEMs; 1-way ANOVA with Dunnett's test (n = 3).

*$p \leq 0.05$, **$p \leq 0.01$, ***$p \leq 0.001$, and ****$p \leq 0.0001$.

growth rate calculation (Figures 1D and 1E). Overall, the flow cytometry data at these two time points correlate well with the RTCA data, further validating its use for assessing biocompatibility.

Dox Loading in UiO-66 and MIL-101(Cr)

After the biocompatibility, internalization, and efficacy of cargo delivery by MIL-101(Cr) and UiO-66 were studied, their drug delivery potential and modes of action were tested. Dox was chosen as the chemotherapeutic agent, due to its clinical use against a broad spectrum of cancers, and it is also one of the few anticancer drugs currently administered as part of a DDS.[32,33] Both MOFs were postsynthetically loaded with Dox by immersion in a solution in Tris-buffered saline, to yield Dox@-MIL-101(Cr) and Dox@UiO-66 (Figures S14–S20; Tables S4–S8). Successful drug loading was indicated by a change in nanoparticle color, from white to red for UiO-66 and from green to red for MIL-101(Cr) (Figure S14). Although some minor peak broadening was observed in the powder X-ray diffraction (PXRD) patterns for both MOFs, no extra peaks were observed, confirming that Dox did not co-crystallize with the MOF nanoparticles (Figures 3A and 3B).

The Dox loading for each MOF was calculated by ultraviolet-visible light (UV-vis) spectroscopic analysis of supernatants, and the drug-loading capacity of the

CellPress
OPEN ACCESS

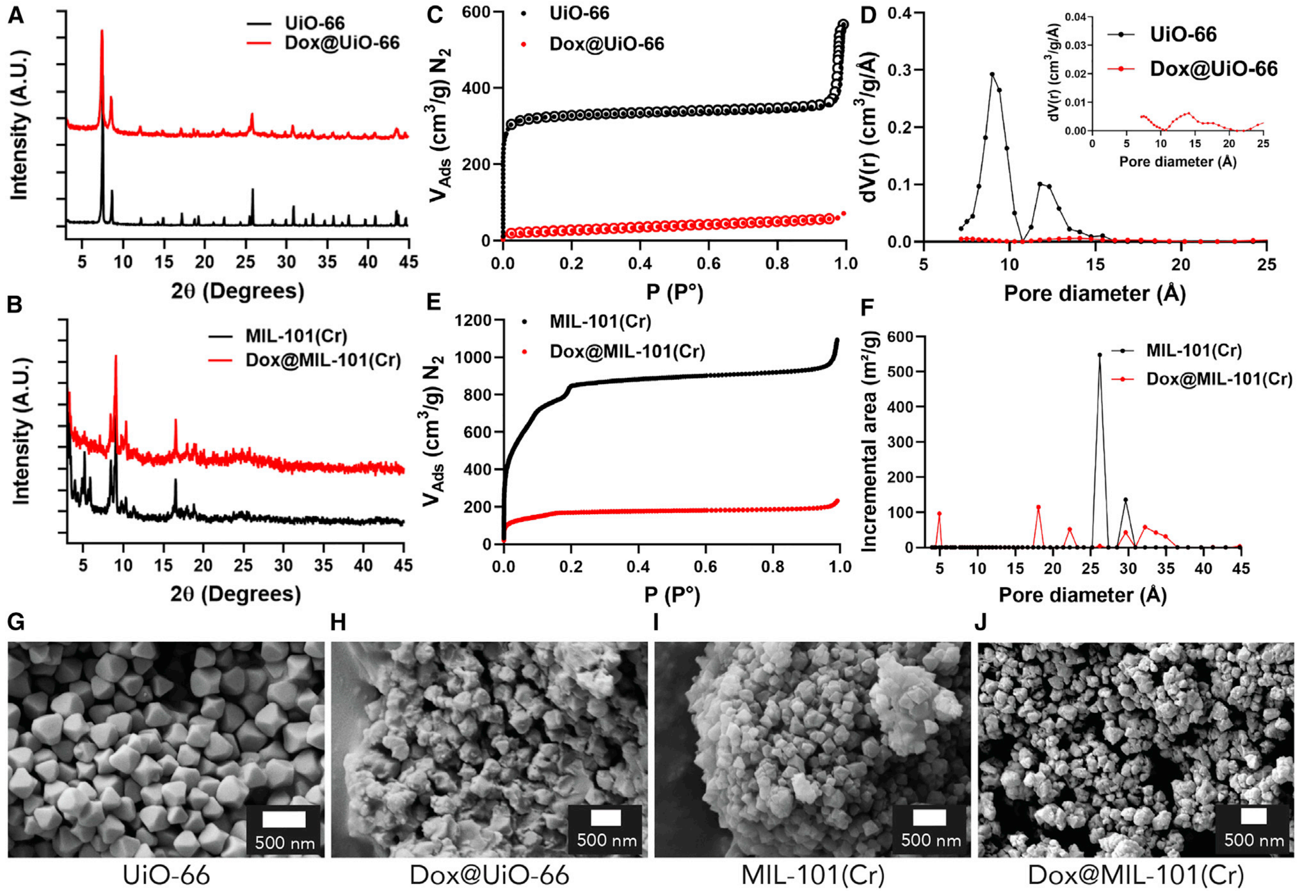

Figure 3. Characterization of Dox@MOF Nanoparticles

(A and B) PXRD patterns of (A) UiO-66 and (B) MIL-101(Cr) MOF nanoparticles, before and after Dox loading.

(C) N_2 adsorption isotherm (77 K) of UiO-66 and Dox@UiO-66.

(D) Pore size distribution plots taken from these isotherms, with a scaled-up trace for Dox@UiO-66 given as an inset (carbon, slit pore, quenched solid density functional theory [QSDFT], equilibrium calculation model).

(E) N_2 adsorption isotherm (77 K) of MIL-101(Cr) and Dox@MIL-101(Cr).

(F) Pore size distribution plots taken from these isotherms (N_2@77 carbon cylinder pores, multi-wall nanotube [MWNT], non-local DFT [NLDFT] calculation model).

(G–J) SEM images of (G) UiO-66, (H) Dox@UiO-66, (I) MIL-101(Cr), and (J) Dox@MIL-101(Cr). Scale bars represent 500 nm.

materials was calculated based on grams of drug per grams of drug-loaded material (wt%). This was calculated at 58 wt% for UiO-66 and 10 wt% for MIL-101(Cr), and the difference in loading values clearly indicates different loading mechanisms. The value for MIL-101(Cr) is commensurate with previous work on MIL-101(Fe) and derivatives (11–13 wt%),[64,65] while similar very high Dox-loading values have also been reported for UiO-66,[52] even on samples with previously modified external surfaces.[35] By thermogravimetric analysis (TGA) analysis, an increase in the organic content was obvious in both cases, indicative of successful drug loading (Figure S16). For Dox@UiO-66, thermal degradation was observed at a lower temperature than the non-loaded MOF, a general trend that has been observed for surface functionalized MOFs.[72,75] It is hypothesized that, due to the added organic functionality at the external surface having a lower thermal stability, combustion is initiated at a lower temperature, and that triggers earlier thermal decomposition of the material as a whole. For Dox@MIL-101(Cr) however, thermal decomposition began almost at the same stage as MIL-101(Cr), but the overall MOF degradation occurred at a

slightly higher temperature. We hypothesize that this observation is indicative of different modes of Dox loading across the two MOFs; MIL-101(Cr) has large enough pores to accommodate the Dox cargo, and having loaded pores could contribute extra thermal stability to the structure.

To further understand the drug-loading mechanism in each instance, the Brunauer-Emmett-Teller (BET) surface area of the drug-loaded materials was measured from N_2 adsorption isotherms at 77 K and compared against their non-loaded counterparts. In the case of Dox@UiO-66, the BET surface area showed a dramatic decrease from 1,324 m^2 g^{-1} for the empty nanoparticles to 95 m^2 g^{-1} for the drug-loaded MOFs (Figure 3C). Bearing in mind that 58 wt% of the Dox@UiO-66 is non-porous Dox, and therefore a significant decrease in gravimetric surface area would be expected in any case, it is clear that the majority of the porosity is blocked by the accumulation of Dox on the external surface of UiO-66; pore loading to this extent would completely remove any residual porosity. This was further validated by examination of the pore size distribution plot (Figure 3D). The average primary pore diameter for the unloaded material was 13 Å and for the secondary pore it was ~9 Å, correlating well with the theoretical values of 11 and 8 Å, respectively. For Dox@UiO-66, however, while the experimental pore size volume of this material was essentially zero, a small trace with similar pore dimensions could be observed, suggestive of blocking access to the majority of pores rather than modifying their geometry through occupation and further validating that Dox molecules have blocked the porosity of UiO-66 by covering its external surface. For MIL-101(Cr), a decrease in its BET surface area from 3,041 to 623 m^2 g^{-1} was observed after drug loading (Figure 3E). The fact that a significant proportion of the internal porosity is still accessible after drug loading suggests that, for Dox@MIL-101(Cr), Dox did not completely cover the external surface of the nanoparticles and block pore access, but penetrated the porosity without completely filling it, although some external surface deposition cannot be ruled out. This correlates with the TGA observations, and is further confirmed by the pore size distribution graph of Dox@MIL-101(Cr), where the main peak at 26 Å (representative of the 25 Å pore) in the bimodal distribution disappears and smaller residual peaks appear (Figure 3F), again suggestive of Dox localization within the pores.

A difference in drug-loading mode between the two materials is suggested by the physical characterization data and is also evident by scanning electron microscopy (SEM). The octahedral morphology of UiO-66 (Figure 3G) changes dramatically on loading, with Dox forming visible layers on the external surfaces of the UiO-66 nanoparticles (Figure 3H). For MIL-101(Cr), the MOF morphology (Figure 3I) was maintained on the formation of Dox@MIL-101(Cr), with Dox being mostly encapsulated inside the pores and not notably visible on the external surface of the material (Figure 3J). This is a plausible physicochemical characteristic for such MOFs, as the Dox molecule is too large (maximum diameter 15 Å)[58] to fit the pores of UiO-66 (pore window 6 Å). Therefore, any attachment to the nanoparticles occurs on their external surfaces, but can penetrate MIL-101(Cr), whose largest pore window is 16.0 Å in diameter, and thus Dox internalization is possible. In addition, the high affinity of Dox for the surface of UiO-66 could be a consequence of electrostatic interactions between the negatively charged surface of UiO-66, as confirmed by zeta potential measurement (Figure S6), and a cargo that would be expected to have residual protonation at neutral pH. In contrast, MIL-101(Cr) was measured to have a positive surface potential. We hypothesize that this major difference in drug-loading modes

could result in different drug delivery and cytotoxic efficiency mechanisms between the two systems.

To validate our hypothesis, we used grand canonical Monte Carlo (GCMC) simulations to investigate Dox loading (see Supplemental Experimental Procedures).[76] When exploring Dox loading in a UiO-66 model devoid of crystalline defects and completely activated, we observed zero uptake as a consequence of the size mismatch between the adsorbate and the pore cavities (i.e., Dox molecules do not fit inside the microporosity of UiO-66). Importantly, a GCMC simulation cannot distinguish between open and closed porosity since the molecules are inserted inside the pores and do not need to be transported, unlike in experiments, though the pore windows. This observation strongly suggests that the loading must occur through adsorption on the external surface of the UiO-66 particles. In contrast, simulations on MIL-101(Cr) yielded a theoretical maximum loading of 1.16 g of Dox per 1 g MOF, which is ~10 times higher than what we observed experimentally, confirming that the pore adsorption of Dox is possible in MIL-101(Cr). It should be noted that, when running these simulations, we did not take into account competing solvents. Although previous simulations showed that not including the solvent in similar systems had a negligible effect,[76,77] this could explain why experimental loading values are lower than the theoretical maximum. In any case, it is also noteworthy that the Dox theoretical maximum loading only occupies 58% of the available pore volume in MIL-101(Cr) due to inefficient packing of the rigid Dox molecule. This suggests that, in Dox@MIL-101(Cr), only 5.3% of the total pore volume is occupied, explaining the sizeable remaining porosity observed in its N_2 adsorption isotherm. These simulations clearly support the hypothesis of external surface loading in Dox@UiO-66 and pore loading in Dox@MIL-101(Cr).

Delivery Profiles of Dox@MIL-101(Cr) and Dox@UiO-66

For the drug delivery evaluation experiments, the highest completely non-toxic concentration of each MOF was used, 1 and 10 $\mu g\ mL^{-1}$ for UiO-66 and MIL-101(Cr), respectively. This entailed concentrations of 2.38 $\mu g\ mL^{-1}$ Dox@UiO-66 and 11.1 $\mu g\ mL^{-1}$ Dox@MIL-101(Cr) being used. In a similar manner, the concentrations of equivalent amounts of free Dox were calculated to be 1.38 $\mu g\ mL^{-1}$ for Dox@UiO-66 and 1.1 $\mu g\ mL^{-1}$ for Dox@MIL-101(Cr). Therefore, due to the significantly lower drug-loading capacity of Dox@MIL-101(Cr) compared to Dox@UiO-66, both systems could essentially transfer a similar amount of Dox to the cells at these concentrations. To identify the mode of action of the different DDSs, the effect of Dox@-MOFs administration over time was monitored by RTCA for 72 h in 3 different cancer cell lines, comprising MCF-7, HepG2, and human ovarian carcinoma (A2780ADR) (see Supplemental Experimental Procedures).

The administration of free Dox inhibited cell growth quickly, at ~4–12 h after addition depending on cell line, and killed all the cells present in the cultures (Figure 4). Dox@MIL-101(Cr) treatment, in comparison to free Dox, exhibited a small delay of 10–16 h before cytotoxicity was observed (Figures 4A–4C). After an immediate decrease in the cell index due to the agitation of the well plates upon the addition of material, which occurs in all of the experiments, an initial small increase in the cell index following Dox@MIL-101(Cr) administration was observed, which closely matched that of the empty MIL-101(Cr) control. This can be attributed to cells internalizing the drug-loaded nanoparticles and causing an increase in cell size, as well as uninhibited cell growth before Dox release. Hence, the observed delay in cytotoxicity is explained by the necessity for the release of the Dox cargo inside the cells

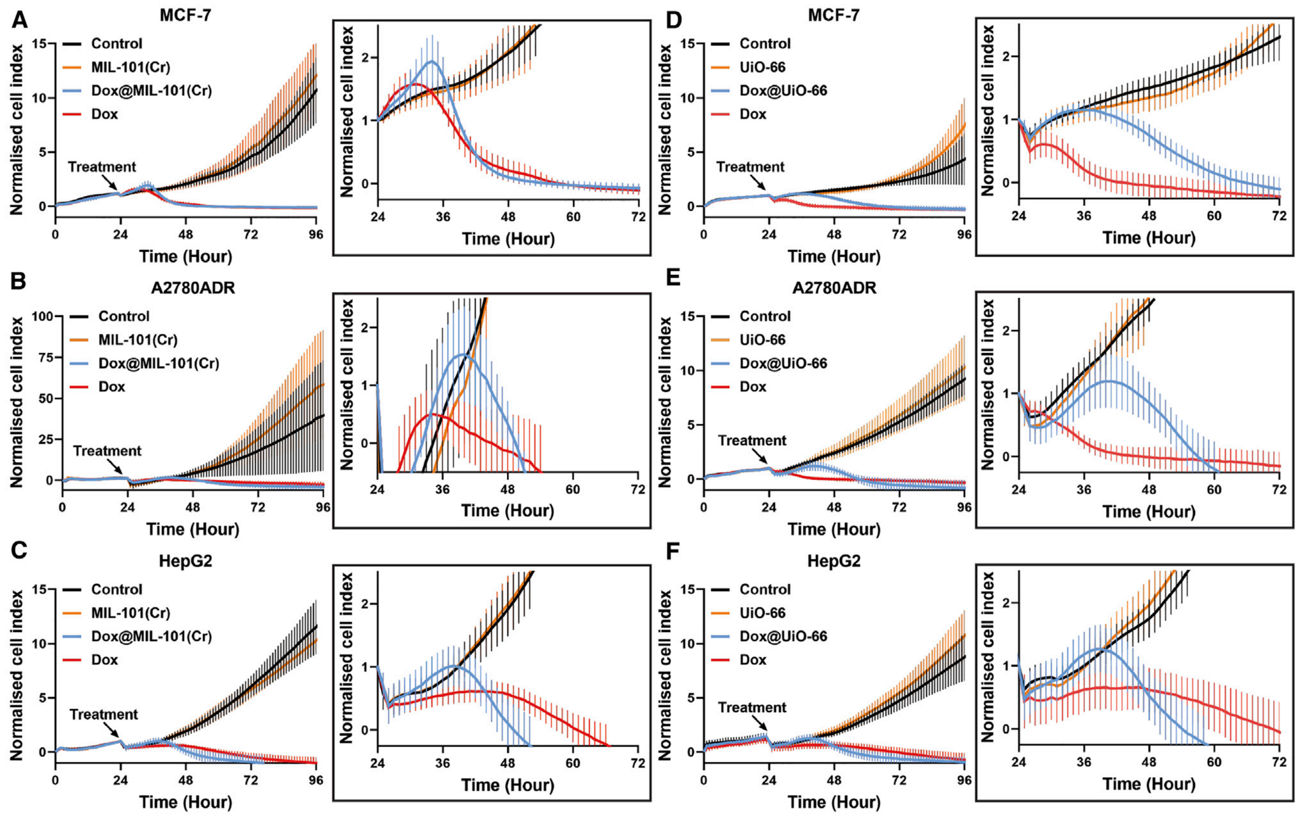

Figure 4. Dox@MIL-101(Cr) and Dox@UiO-66 Are Therapeutically Efficacious and Have Different Dox Delivery Profiles

(A–C) RTCA of 3 cancer cell lines treated with empty MIL-101(Cr), Dox@MIL-101(Cr), and free Dox. The cancer cell lines were allowed to grow for 24 h after the initial cell seeding at time point 0. Treatment administration took place at 24 h. The data are presented as means ± SDs (n = 6).

(D–F) RTCA of 3 cancer cell lines treated with empty UiO-66, Dox@UiO-66, and free Dox. The cancer cell lines were allowed to grow for 24 h after the initial cell seeding at time point 0. Treatment administration took place at 24 h. The data are presented as means ± SDs (n = 6).

and the subsequent delay in its transfer into the cell nucleus, where Dox is active. Nevertheless, once sufficient amounts of the Dox reached the cell nucleus, cell death ensued and the cell index quickly returned to a profile aligned with that of free Dox, or below it.

In contrast, Dox@UiO-66 demonstrated a significantly longer delay in mediating cytotoxicity in all of the cancer cell lines tested (Figures 4D–4F), compared to both free Dox and Dox@MIL-101(Cr). After the addition of Dox@UiO-66, the cell index continued to increase for 14–20 h, depending on the cell line, similar to the controls. This increased uninhibited cell growth period could be attributed to a slower release rate of Dox. The initial cell growth was eventually followed by inhibition, a subsequent cytotoxicity-associated decrease in cell index, and finally, cell death. The cell index profiles returned to track those of free Dox after ~36 h incubation for MCF-7 (Figures 4A and 4D) and A2780ADR (Figures 4B and 4E) cells, and after ~24 h incubation for HepG2 cells (Figures 4C and 4F). It is notable that the difference in cytotoxicity onset time between the two Dox-loaded MOFs is less significant for HepG2 cells, but still apparent, and both drug-loaded MOFs induce cytotoxicity faster overall compared to free Dox. Complete cell death takes ~24–26 h for Dox@-MIL-101(Cr) and 34–36 h for Dox@UiO-66. Single-point assays are typically measured 24 and 72 h after incubation with DDSs; it is, therefore, worthwhile to note that, in this case, assays after 24 h of incubation would suggest some cell-

specific selectivity in anticancer efficacy for Dox@UiO-66, despite this not being true overall, and if assessing cytotoxicity after 72 h this difference in behavior between Dox@UiO-66 and Dox@MIL-101(Cr) would not have been apparent. Nevertheless, both materials were therapeutically efficacious in facilitating cancer drug delivery and cytotoxicity toward cancer cells.

Since both materials are very efficiently internalized by cells (Figure 2), their different time-dependent cytotoxicities could be attributed to different Dox release mechanisms. The apparent faster drug release from Dox@MIL-101(Cr) compared to Dox@-UiO-66 is likely a consequence of the different mode of drug loading in each system. In the instance of Dox@UiO-66, Dox is deposited primarily on the outer surface of the nanoparticles. The particles, therefore, present an external surface layer of insoluble, aggregated Dox, which is likely to be difficult to digest once inside the cells, as this process would involve disassociation/dissolution of the strongly aggregated drug molecules. For Dox@MIL-101(Cr), Dox is primarily located inside the porosity of the framework and presumably well dispersed, as it only takes up a small fraction of the pore volume, making its release easier and faster, either through desorption or intracellular digestion of the MOF. To test this hypothesis, we used time-dependent confocal microscopy to monitor the intracellular release and subsequent nuclear localization of Dox from Dox@MIL-101(Cr) and Dox@UiO-66 in MCF-7 cells.

Dox@MIL-101(Cr) Enhances Delivery and Subsequent Release of Dox in MCF-7 Cells

Quantification of the intracellular and intranuclear fluorescence intensity of Dox by confocal microscopy (see Supplemental Experimental Procedures), following administration in MCF-7 cells as a free agent or as part of the MIL-101(Cr) drug delivery system, was performed at both 4 and 8 h post-administration, as RTCA demonstrated that significant cytotoxicity becomes apparent at ~12 h after incubation. After 4 h of treatment, the intracellular concentration of Dox was equivalent for both the free molecule and the DDS (Figure 5A). Nuclear quantification at 4 h revealed that intranuclear Dox was significantly higher for the free molecule, co-localizing with the DAPI stain, while Dox@MIL-101(Cr) could be seen in both cytoplasmic and nuclear localization. The red emission is visually more diffuse across the cell for Dox@MIL-101(Cr) compared to free Dox, but this is not directly reflected in our quantified data, as the intensity of Dox fluorescence only from the cytoplasm cannot be measured with a high level of confidence by our experimental protocol. This is consistent with Dox being held within the nanocarrier in the cell cytoplasm and then migrating to the nucleus on release, and in keeping with a thesis of an immediate cytotoxic effect for the free drug, supporting our hypothesis that a drug-release step delays Dox@MIL-101(Cr)-mediated cytotoxicity. At 8 h post-treatment, both intracellular and intranuclear Dox concentrations were significantly higher for Dox@-MIL-101(Cr), outperforming the free molecule (Figure 5B). The exceptional internalization capacity of MIL-101(Cr), along with the relatively rapid intracellular Dox release, result in higher amounts of Dox reaching the nucleus when administered as part of the DDS compared to free molecule administration. This suggests that lower concentrations of Dox could be used, and therefore its intrinsic off-target toxicity could be minimized or avoided.

Dox@UiO-66 Delays the Delivery and Subsequent Release of Dox in MCF-7 Cells

As with Dox@MIL-101(Cr), quantification of the intracellular and intranuclear Dox levels in MCF-7 cells after incubation with Dox@UiO-66 was achieved by calculation of the fluorescence intensity of Dox after co-localization with the cytoplasmic or

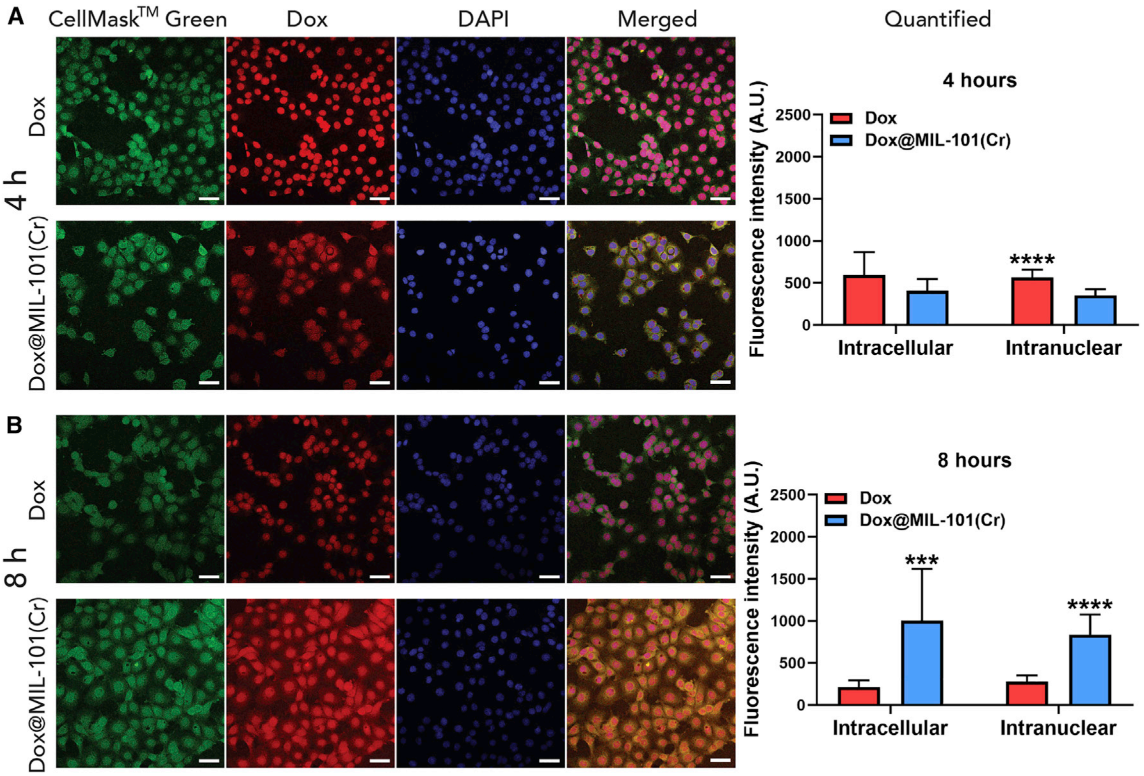

Figure 5. Dox@MIL-101(Cr) Enhances the Delivery and Subsequent Release of Dox in MCF-7 Cells
Confocal microscopy and fluorescence quantification of free Dox and Dox@MIL-101(Cr) delivery in MCF-7 cells (A) 4 and (B) 8 h post-treatment administration. The concentration that was used for free Dox corresponds to the amount of Dox loaded in Dox@UiO-66. Green: cell membrane stain, red: Dox, blue: nuclear DAPI stain. Scale bars, 50 μm. Quantification of Dox delivery capacity through measurements of total intracellular and intranuclear fluorescence. The data are presented as means ± SDs; Student's t test (n = 10).
*$p \leq 0.05$, **$p \leq 0.01$, ***$p \leq 0.001$, and ****$p \leq 0.0001$ (comparison between Dox and Dox@MIL-101(Cr) treatments).

nuclear dye, respectively (see Supplemental Experimental Procedures). After 4 h, the absolute intracellular fluorescence value of free Dox was significantly higher when administered as a free drug compared to the DDS (Figure 6A). However, after 12 (Figure 6B) and 24 h (Figure 6C) of treatment (times based on the delay and onset of cytotoxicity by RTCA), there was no statistical difference in Dox fluorescence between the free drug and Dox@UiO-66. This indicates that prolonged incubation times yield equally potent intracellular Dox concentrations as the free drug.

In addition, at all time points, free Dox was found primarily in the cell nucleus, strongly co-localizing with the nuclear stain, while Dox from Dox@UiO-66 was seen in both the nucleus and the cytoplasm of the cells, with a diffuse red emission similar to Dox@MIL-101(Cr), and indicative of Dox being associated with the nano-carrier in the cytoplasm before or during release. These observations suggest that Dox@UiO-66 releases Dox slowly into the cytoplasm, notably slower than Dox@MIL-101(Cr), further strengthening our hypothesis that the delayed cytotoxic effects observed with RTCA are associated with the drug-release profile of the material in question. The difference in intracellular Dox release kinetics points to different release mechanisms for the two MOFs, which reflects the fact that Dox is loaded in the internal porosity of Dox@MIL-101(Cr) and on the external surface of Dox@UiO-66. Moreover, at all time points, Dox demonstrated

CellPress
OPEN ACCESS

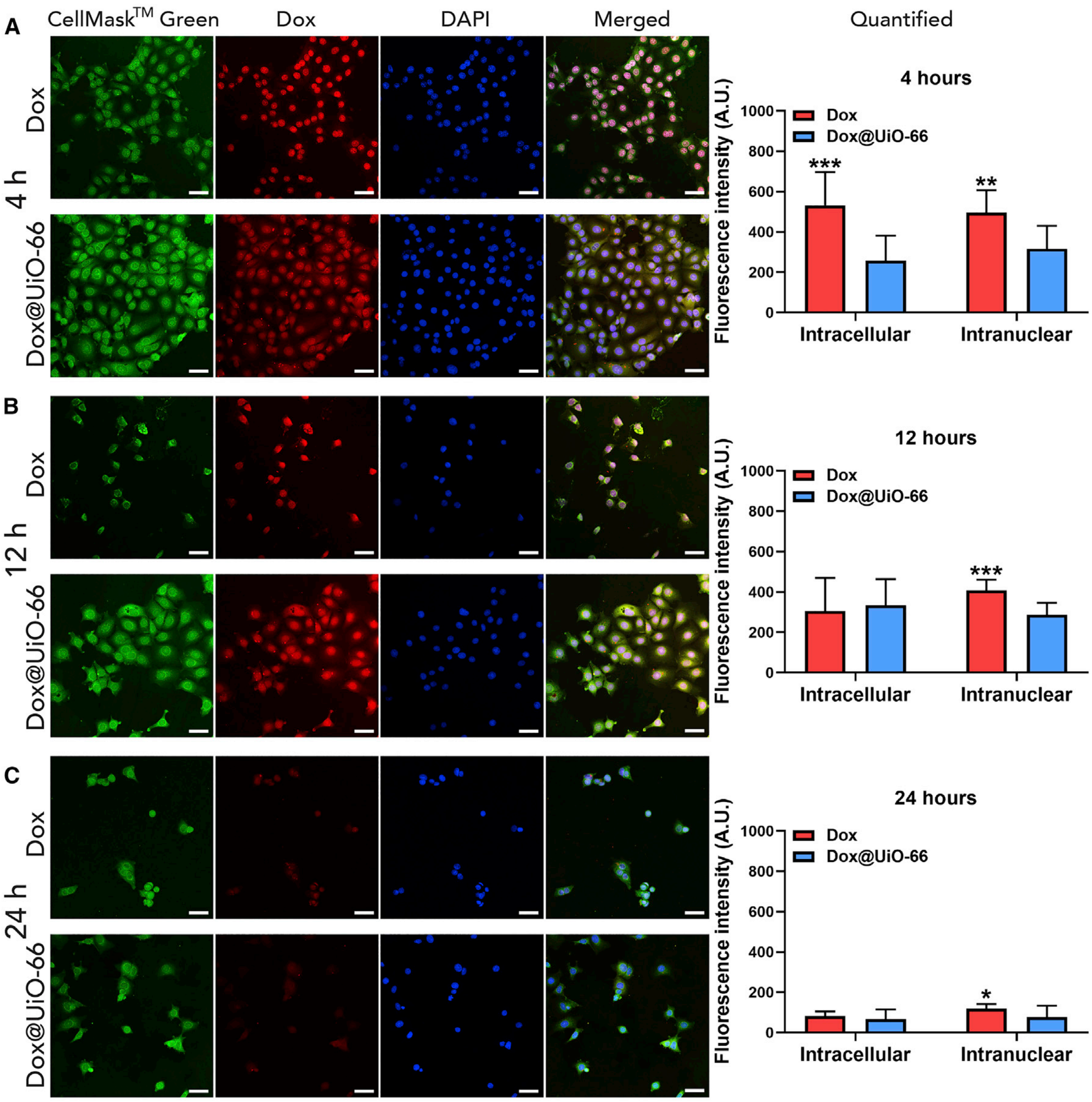

Figure 6. Dox@UiO-66 Delays the Delivery and Subsequent Release of Dox in MCF-7 Cells

(A–C) Confocal microscopy and fluorescence quantification of free Dox and Dox@UiO-66 delivery in MCF-7 cells (A) 4, (B) 12, and (C) 24 h post-treatment administration. The concentration that was used for free Dox corresponds to the amount of Dox loaded in Dox@UiO-66. Green: cell membrane stain, red: Dox, blue: nuclear DAPI stain. Scale bars, 50 μm. Quantification of Dox delivery capacity through measurements of total intracellular and intranuclear fluorescence. The data are presented as means ± SDs; Student's t test (n = 10).
*$p \le 0.05$, **$p \le 0.01$, ***$p \le 0.001$ (comparison between Dox and Dox@UiO-66 treatments).

higher nuclear internalization when administrated as a free molecule than as part of Dox@UiO-66, explaining the faster cytotoxic effect observed previously with the RTCA experiments.

By using a suite of complementary *in vitro* techniques, we have shown that RTCA is a highly informative technique for assessing the biocompatibility of nanoscale MOFs. Proof-of-concept work has shown that MIL-101(Cr) and UiO-66 have excellent biocompatibility, internalization efficiency, and significantly enhanced calcein cargo delivery across a series of cell types. Dox was chosen as a chemotherapeutic probe

for drug delivery, and was found to attach to the external surface of UiO-66 nanoparticles, forming layers on top of them, while the larger pore cavities of MIL-101(Cr) allowed for penetration to the internal porosity. RTCA was used to monitor drug delivery, demonstrating that these differences in loading have a direct effect on the drug-release rate within cancer cells, affecting cytotoxic efficacy in a time-dependent manner that could easily be missed by the single-point assays commonly used to probe cytotoxicity. Confocal fluorescence microscopy confirmed different intracellular Dox release rates that closely corresponded to the RTCA measurements. Due to the Dox loading mode of Dox@UiO-66, drug release more likely resembles the dissolution of a Dox nanoparticle than the degradation of the MOF carrier, subsequently delaying the release of the drug and its cytotoxic action. Conversely, Dox@MIL-101(Cr) nanoparticles display a more rapid drug release, occurring via a combination of Dox desorption and degradation of the MOF carrier, leading to a significantly large intracellular accumulation of Dox compared to the free drug. The characterization of drug loading in MOFs rarely extends to pinpointing its locality; our data indicate that the relationship between drug-loading mode and MOF-mediated drug delivery clearly must be taken into consideration in the development of therapeutically efficacious MOF DDSs, and that surface loading Dox on MOF nanoparticles may explain slow release trends observed previously.

This more controlled killing effect of Dox@MOFs, in contrast to the immediate effect of the free cancer drug, makes the translatability of these materials very promising for use as DDSs. As current DDSs do not typically achieve enhanced Dox efficiency, MIL-101(Cr) is a very promising candidate for this type of application, as higher levels of Dox internalization can be achieved. This indicates that a lower drug concentration could be used, allowing for the development of a therapeutic strategy with the potential to limit the undesirable off-target acute toxicity of chemotherapy. It is also compatible with locoregional drug administration strategies, such as in head and neck cancers, where it may enable the sustained local release of a chemotherapeutic while limiting collateral damage to surrounding healthy tissue. While these proof-of-concept mechanistic experiments have been carried out on bare MOFs, the pore loading of Dox in MIL-101(Cr) is also compatible with further functionalization to enhance targeting and biodistribution as we move toward *in vivo* experiments to assess clinical translatability.

EXPERIMENTAL PROCEDURES

Resource Availability

Lead Contact

Further information and requests for resources and reagents should be directed to and will be fulfilled by the Lead Contact, Prof. Ross Forgan (ross.forgan@glasgow.ac.uk).

Materials Availability

All solvents and reagents were purchased from Alfa Aesar, Acros Organics, Sigma-Aldrich, Merck, Tokyo Chemical Industry, Thermo Fisher Scientific, and Zymo Research USA, and used without further purification (see Supplemental Experimental Procedures).

Data and Code Availability

All of the data are presented within the article and Supporting Information and are available to download from https://dx.doi.org/10.5525/gla.researchdata.1072.

Synthesis and Characterization

All of the experimental procedures are listed in the Supplemental Experimental Procedures.

SUPPLEMENTAL INFORMATION

Supplemental Information can be found online at https://doi.org/10.1016/j.xcrp.2020.100254.

ACKNOWLEDGMENTS

R.S.F. and D.F.-J. thank The Royal Society for receipt of University Research Fellowships and acknowledge funding from EPSRC (EP/S009000/1) and the European Research Council (ERC) under the European Union's Horizon 2020 research and innovation programme, ERC-2016-COG 726380 (NanoMODdeli), and ERC-2015-STG-677289 (SCoTMOF). Computational analysis was supported by the Cambridge High-Performance Computing Service and the Cambridge Service for Data-Driven Discovery (CSD3).

AUTHOR CONTRIBUTIONS

P.M., N.P., D.F.-J., P.G.S., and R.S.F. wrote and edited the manuscript. P.M., N.P., P.G.S., and R.S.F. designed the study. P.M. synthesized the MOFs, performed the calcein and doxorubicin loading, characterized the materials, and performed the Alamar blue assays. P.M. and N.P. performed the flow cytometry and RTCA experiments, analyzed the data, and plotted the figures. N.P. performed the confocal microscopy experiments. A.L. and R.B.-P. carried out the computational simulations, and D.M. performed the N_2 adsorption measurements for MIL-101(Cr) and Dox@-MIL-101(Cr) in the D.F.-J. laboratory. S.B. provided technical assistance.

DECLARATION OF INTERESTS

The authors declare no competing interests.

Received: August 11, 2020
Revised: October 1, 2020
Accepted: October 16, 2020
Published: November 18, 2020

REFERENCES

1. Horcajada, P., Gref, R., Baati, T., Allan, P.K., Maurin, G., Couvreur, P., Férey, G., Morris, R.E., and Serre, C. (2012). Metal-organic frameworks in biomedicine. Chem. Rev. *112*, 1232–1268.

2. Simon-Yarza, T., Mielcarek, A., Couvreur, P., and Serre, C. (2018). Nanoparticles of Metal-Organic Frameworks: On the Road to In Vivo Efficacy in Biomedicine. Adv. Mater. *30*, e1707365.

3. Giménez-Marqués, M., Hidalgo, T., Serre, C., and Horcajada, P. (2016). Nanostructured metal–organic frameworks and their bio-related applications. Coord. Chem. Rev. *307*, 342–360.

4. Huxford, R.C., Della Rocca, J., and Lin, W. (2010). Metal-organic frameworks as potential drug carriers. Curr. Opin. Chem. Biol. *14*, 262–268.

5. Rojas, S., Arenas-Vivo, A., and Horcajada, P. (2019). Metal-organic frameworks: a novel platform for combined advanced therapies. Coord. Chem. Rev. *388*, 202–226.

6. Baeza, A., Ruiz-Molina, D., and Vallet-Regí, M. (2017). Recent advances in porous nanoparticles for drug delivery in antitumoral applications: inorganic nanoparticles and nanoscale metal-organic frameworks. Expert Opin. Drug Deliv. *14*, 783–796.

7. Forgan, R.S. (2020). Modulated self-assembly of metal–organic frameworks. Chem. Sci. (Camb.) *11*, 4546–4562.

8. Marshall, C.R., Staudhammer, S.A., and Brozek, C.K. (2019). Size control over metal-organic framework porous nanocrystals. Chem. Sci. (Camb.) *10*, 9396–9408.

9. Wang, S., McGuirk, C.M., d'Aquino, A., Mason, J.A., and Mirkin, C.A. (2018). Metal-Organic Framework Nanoparticles. Adv. Mater. *30*, e1800202.

10. McGuire, C.V., and Forgan, R.S. (2015). The surface chemistry of metal-organic frameworks. Chem. Commun. (Camb.) *51*, 5199–5217.

11. Furukawa, H., Go, Y.B., Ko, N., Park, Y.K., Uribe-Romo, F.J., Kim, J., O'Keeffe, M., and Yaghi, O.M. (2011). Isoreticular expansion of metal-organic frameworks with triangular and square building units and the lowest calculated density for porous crystals. Inorg. Chem. *50*, 9147–9152.

12. Yaghi, O.M., O'Keeffe, M., Ockwig, N.W., Chae, H.K., Eddaoudi, M., and Kim, J. (2003). Reticular synthesis and the design of new materials. Nature *423*, 705–714.

13. Cai, M., Chen, G., Qin, L., Qu, C., Dong, X., Ni, J., and Yin, X. (2020). Metal Organic Frameworks as Drug Targeting Delivery

Vehicles in the Treatment of Cancer. Pharmaceutics *12*, 232.

14. Haddad, S., Abánades Lázaro, I., Fantham, M., Mishra, A., Silvestre-Albero, J., Osterrieth, J.W.M., Kaminski Schierle, G.S., Kaminski, C.F., Forgan, R.S., and Fairen-Jimenez, D. (2020). Design of a Functionalized Metal-Organic Framework System for Enhanced Targeted Delivery to Mitochondria. J. Am. Chem. Soc. *142*, 6661–6674.

15. Röder, R., Preiß, T., Hirschle, P., Steinborn, B., Zimpel, A., Höhn, M., Rädler, J.O., Bein, T., Wagner, E., Wuttke, S., and Lächelt, U. (2017). Multifunctional Nanoparticles by Coordinative Self-Assembly of His-Tagged Units with Metal-Organic Frameworks. J. Am. Chem. Soc. *139*, 2359–2368.

16. Teplensky, M.H., Fantham, M., Poudel, C., Hockings, C., Lu, M., Guna, A., Aragones-Anglada, M., Moghadam, P.Z., Li, P., Farha, O.K., et al. (2019). A Highly Porous Metal-Organic Framework System to Deliver Payloads for Gene Knockdown. Chem *5*, 2926–2941.

17. Alsaiari, S.K., Patil, S., Alyami, M., Alamoudi, K.O., Aleisa, F.A., Merzaban, J.S., Li, M., and Khashab, N.M. (2018). Endosomal Escape and Delivery of CRISPR/Cas9 Genome Editing Machinery Enabled by Nanoscale Zeolitic Imidazolate Framework. J. Am. Chem. Soc. *140*, 143–146.

18. Zhuang, J., Gong, H., Zhou, J., Zhang, Q., Gao, W., Fang, R.H., and Zhang, L. (2020). Targeted gene silencing in vivo by platelet membrane-coated metal-organic framework nanoparticles. Sci. Adv. *6*, eaaz6108.

19. Hidalgo, T., Alonso-Nocelo, M., Bouzo, B.L., Reimondez-Troitiño, S., Abuin-Redondo, C., de la Fuente, M., and Horcajada, P. (2020). Biocompatible iron(iii) carboxylate metal-organic frameworks as promising RNA nanocarriers. Nanoscale *12*, 4839–4845.

20. Cai, W., Wang, J., Chu, C., Chen, W., Wu, C., and Liu, G. (2018). Metal-Organic Framework-Based Stimuli-Responsive Systems for Drug Delivery. Adv. Sci. (Weinh.) *6*, 1801526.

21. Wang, Y., Yan, J., Wen, N., Xiong, H., Cai, S., He, Q., Hu, Y., Peng, D., Liu, Z., and Liu, Y. (2020). Metal-organic frameworks for stimuli-responsive drug delivery. Biomaterials *230*, 119619.

22. Carrillo-Carrión, C. (2020). Nanoscale metal-organic frameworks as key players in the context of drug delivery: evolution toward theranostic platforms. Anal. Bioanal. Chem. *412*, 37–54.

23. Pala, R., Pattnaik, S., Zeng, Y., Busi, S., Nauli, S.M., and Liu, G. (2020). Functional MOFs as molecular imaging probes and theranostics. In Metal-Organic Frameworks for Biomedical Applications, M. Mozafari, ed. (Woodhead Publishing), pp. 425–443.

24. Zhang, Z., Sang, W., Xie, L., and Dai, Y. (2019). Metal-organic frameworks for multimodal bioimaging and synergistic cancer chemotherapy. Coord. Chem. Rev. *399*, 213022.

25. Lu, K., Aung, T., Guo, N., Weichselbaum, R., and Lin, W. (2018). Nanoscale Metal-Organic Frameworks for Therapeutic, Imaging, and Sensing Applications. Adv. Mater. *30*, e1707634.

26. Zhao, H., Serre, C., Dumas, E., and Steunou, N. (2020). Functional MOFs as theranostics. In Metal-Organic Frameworks for Biomedical Applications, M. Mozafari, ed. (Woodhead Publishing), pp. 397–423.

27. Lismont, M., Dreesen, L., and Wuttke, S. (2017). Metal-Organic Framework Nanoparticles in Photodynamic Therapy: Current Status and Perspectives. Adv. Funct. Mater. *27*, 1606314.

28. Guan, Q., Li, Y.-A., Li, W.-Y., and Dong, Y.-B. (2018). Photodynamic Therapy Based on Nanoscale Metal-Organic Frameworks: From Material Design to Cancer Nanotherapeutics. Chem. Asian J. *13*, 3122–3149.

29. Grall, R., Hidalgo, T., Delic, J., Garcia-Marquez, A., Chevillard, S., and Horcajada, P. (2015). In vitro biocompatibility of mesoporous metal (III; Fe, Al, Cr) trimesate MOF nanocarriers. J. Mater. Chem. B Mater. Biol. Med. *3*, 8279–8292.

30. Wuttke, S., Braig, S., Preiß, T., Zimpel, A., Sicklinger, J., Bellomo, C., Rädler, J.O., Vollmar, A.M., and Bein, T. (2015). MOF nanoparticles coated by lipid bilayers and their uptake by cancer cells. Chem. Commun. (Camb.) *51*, 15752–15755.

31. Motlagh, N.S.H., Parvin, P., Ghasemi, F., and Atyabi, F. (2016). Fluorescence properties of several chemotherapy drugs: doxorubicin, paclitaxel and bleomycin. Biomed. Opt. Express *7*, 2400–2406.

32. Barenholz, Y. (2012). Doxil®–the first FDA-approved nano-drug: lessons learned. J. Control. Release *160*, 117–134.

33. Swenson, C.E., Perkins, W.R., Roberts, P., and Janoff, A.S. (2001). Liposome technology and the development of Myocet™ (liposomal doxorubicin citrate). Breast *10*, 1–7.

34. Feng, J., Xu, Z., Dong, P., Yu, W., Liu, F., Jiang, Q., Wang, F., and Liu, X. (2019). Stimuli-responsive multifunctional metal-organic framework nanoparticles for enhanced chemo-photothermal therapy. J. Mater. Chem. B Mater. Biol. Med. *7*, 994–1004.

35. Chen, D., Yang, D., Dougherty, C.A., Lu, W., Wu, H., He, X., Cai, T., Van Dort, M.E., Ross, B.D., and Hong, H. (2017). In Vivo Targeting and Positron Emission Tomography Imaging of Tumor with Intrinsically Radioactive Metal-Organic Frameworks Nanomaterials. ACS Nano *11*, 4315–4327.

36. Chen, W.-H., Yu, X., Cecconello, A., Sohn, Y.S., Nechushtai, R., and Willner, I. (2017). Stimuli-responsive nucleic acid-functionalized metal-organic framework nanoparticles using pH- and metal-ion-dependent DNAzymes as locks. Chem. Sci. (Camb.) *8*, 5769–5780.

37. Chen, W.-H., Yang Sung, S., Fadeev, M., Cecconello, A., Nechushtai, R., and Willner, I. (2018). Targeted VEGF-triggered release of an anti-cancer drug from aptamer-functionalized metal-organic framework nanoparticles. Nanoscale *10*, 4650–4657.

38. Yang, X., Li, L., He, D., Hai, L., Tang, J., Li, H., He, X., and Wang, K. (2017). A metal-organic framework based nanocomposite with co-encapsulation of Pd@Au nanoparticles and doxorubicin for pH- and NIR-triggered synergistic chemo-photothermal treatment of cancer cells. J. Mater. Chem. B Mater. Biol. Med. *5*, 4648–4659.

39. Zhang, H., Jiang, W., Liu, R., Zhang, J., Zhang, D., Li, Z., and Luan, Y. (2017). Rational Design of Metal Organic Framework Nanocarrier-Based Codelivery System of Doxorubicin Hydrochloride/Verapamil Hydrochloride for Overcoming Multidrug Resistance with Efficient Targeted Cancer Therapy. ACS Appl. Mater. Interfaces *9*, 19687–19697.

40. Sene, S., Marcos-Almaraz, M.T., Menguy, N., Scola, J., Volatron, J., Rouland, R., Grenèche, J.-M., Miraux, S., Menet, C., Guillou, N., et al. (2017). Maghemite-nanoMIL-100(Fe) Bimodal Nanovector as a Platform for Image-Guided Therapy. Chem *3*, 303–322.

41. Shu, F., Lv, D., Song, X.-L., Huang, B., Wang, C., Yu, Y., and Zhao, S.-C. (2018). Fabrication of a hyaluronic acid conjugated metal organic framework for targeted drug delivery and magnetic resonance imaging. RSC Advances *8*, 6581–6589.

42. Zheng, H., Zhang, Y., Liu, L., Wan, W., Guo, P., Nyström, A.M., and Zou, X. (2016). One-pot Synthesis of Metal-Organic Frameworks with Encapsulated Target Molecules and Their Applications for Controlled Drug Delivery. J. Am. Chem. Soc. *138*, 962–968.

43. Lei, J., Wang, H., Zhu, D., Wan, Y., and Yin, L. (2020). Combined effects of avasimibe immunotherapy, doxorubicin chemotherapy, and metal-organic frameworks nanoparticles on breast cancer. J. Cell. Physiol. *235*, 4814–4823.

44. Wang, Z., Tang, X., Wang, X., Yang, D., Yang, C., Lou, Y., Chen, J., and He, N. (2016). Near-infrared light-induced dissociation of zeolitic imidazole framework-8 (ZIF-8) with encapsulated CuS nanoparticles and their application as a therapeutic nanoplatform. Chem. Commun. (Camb.) *52*, 12210–12213.

45. He, M., Zhou, J., Chen, J., Zheng, F., Wang, D., Shi, R., Guo, Z., Wang, H., and Chen, Q. (2015). Fe_3O_4@carbon@zeolitic imidazolate framework-8 nanoparticles as multifunctional pH-responsive drug delivery vehicles for tumor therapy in vivo. J. Mater. Chem. B Mater. Biol. Med. *3*, 9033–9042.

46. Chen, R., Zhang, J., Wang, Y., Chen, X., Zapien, J.A., and Lee, C.-S. (2015). Graphitic carbon nitride nanosheet@metal-organic framework core-shell nanoparticles for photo-chemo combination therapy. Nanoscale *7*, 17299–17305.

47. Kang, Y., Yu, X., Fan, X., Aodenggerile, Zhao, S., Tu, C., Yan, Z., Wang, R., Li, W., and Qiu, H. (2020). Tetramodal Imaging and Synergistic Cancer Radio-Chemotherapy Enabled by Multiple Component-Encapsulated Zeolitic Imidazolate Frameworks. ACS Nano *14*, 4336–4351.

48. Zhang, L., Gao, Y., Sun, S., Li, Z., Wu, A., and Zeng, L. (2020). pH-responsive metal-organic framework encapsulated gold nanoclusters with modulated release to enhance photodynamic therapy/chemotherapy in breast cancer. J. Mater. Chem. B Mater. Biol. Med. *8*, 1739–1747.

49. Jia, X., Yang, Z., Wang, Y., Chen, Y., Yuan, H., Chen, H., Xu, X., Gao, X., Liang, Z., Sun, Y., et al. (2018). Hollow Mesoporous Silica@Metal-Organic Framework and Applications for pH-Responsive Drug Delivery. ChemMedChem *13*, 400–405.

50. Vasconcelos, I.B., da Silva, T.G., Militão, G.C.G., Soares, T.A., Rodrigues, N.M., Rodrigues, M.O., da Costa, N.B., Freire, R.O., and Junior, S.A. (2012). Cytotoxicity and slow release of the anti-cancer drug doxorubicin from ZIF-8. RSC Advances *2*, 9437–9442.

51. Park, K.S., Ni, Z., Côté, A.P., Choi, J.Y., Huang, R., Uribe-Romo, F.J., Chae, H.K., O'Keeffe, M., and Yaghi, O.M. (2006). Exceptional chemical and thermal stability of zeolitic imidazolate frameworks. Proc. Natl. Acad. Sci. USA *103*, 10186–10191.

52. Zhao, H.-X., Zou, Q., Sun, S.-K., Yu, C., Zhang, X., Li, R.-J., and Fu, Y.-Y. (2016). Theranostic metal-organic framework core-shell composites for magnetic resonance imaging and drug delivery. Chem. Sci. (Camb.) *7*, 5294–5301.

53. Chen, S., Chen, Q., Dong, S., Ma, J., Yang, Y.-W., Chen, L., and Gao, H. (2018). Polymer Brush Decorated MOF Nanoparticles Loaded with AIEgen, Anticancer Drug, and Supramolecular Glue for Regulating and In Situ Observing DOX Release. Macromol. Biosci. *18*, e1800317.

54. Li, T., Wu, Q., Wang, W., Chen, Z., Tan, L., Yu, J., Fu, C., Ren, X., Liang, P., Ren, J., et al. (2020). MOF-derived nano-popcorns synthesized by sonochemistry as efficient sensitizers for tumor microwave thermal therapy. Biomaterials *234*, 119773.

55. Cavka, J.H., Jakobsen, S., Olsbye, U., Guillou, N., Lamberti, C., Bordiga, S., and Lillerud, K.P. (2008). A new zirconium inorganic building brick forming metal organic frameworks with exceptional stability. J. Am. Chem. Soc. *130*, 13850–13851.

56. Fairen-Jimenez, D., Moggach, S.A., Wharmby, M.T., Wright, P.A., Parsons, S., and Düren, T. (2011). Opening the gate: framework flexibility in ZIF-8 explored by experiments and simulations. J. Am. Chem. Soc. *133*, 8900–8902.

57. Moggach, S.A., Bennett, T.D., and Cheetham, A.K. (2009). The effect of pressure on ZIF-8: increasing pore size with pressure and the formation of a high-pressure phase at 1.47 GPa. Angew. Chem. Int. Ed. Engl. *48*, 7087–7089.

58. Bilalis, P., Tziveleka, L.-A., Varlas, S., and Iatrou, H. (2016). pH-Sensitive nanogates based on poly(l-histidine) for controlled drug release from mesoporous silica nanoparticles. Polym. Chem. *7*, 1475–1485.

59. Abánades Lázaro, I., and Forgan, R.S. (2019). Application of zirconium MOFs in drug delivery and biomedicine. Coord. Chem. Rev. *380*, 230–259.

60. Gao, S., Hou, J., Deng, Z., Wang, T., Beyer, S., Guilherme Buzanich, A., Richardson, J.J., Rawal, A., Seidel, R., Zulkifli, M.Y., et al. (2019). Improving the Acidic Stability of Zeolitic Imidazolate Frameworks by Biofunctional Molecules. Chem *5*, 1597–1608.

61. Luzuriaga, M.A., Benjamin, C.E., Gaertner, M.W., Lee, H., Herbert, F.C., Mallick, S., and Gassensmith, J.J. (2019). ZIF-8 degrades in cell media, serum, and some—but not all—common laboratory buffers. Supramol. Chem. *31*, 485–490.

62. Velásquez-Hernández, M.J., Ricco, R., Carraro, F., Limpoco, F.T., Linares-Moreau, M., Leitner, E., Wiltsche, H., Rattenberger, J., Schrottner, H., Frühwirt, P., et al. (2019). Degradation of ZIF-8 in phosphate buffered saline media. CrystEngComm *21*, 4538–4544.

63. Férey, G., Mellot-Draznieks, C., Serre, C., Millange, F., Dutour, J., Surblé, S., and Margiolaki, I. (2005). A chromium terephthalate-based solid with unusually large pore volumes and surface area. Science *309*, 2040–2042.

64. Liu, Y., Fan, L., Xu, C., Sun, K., Shi, Z., and Li, L. (2018). MIL-101/CDs/MIL-101 for potential fluorescence imaging and pH-responsive drug delivery. Mater. Lett. *211*, 32–35.

65. Wang, X.-G., Dong, Z.-Y., Cheng, H., Wan, S.-S., Chen, W.-H., Zou, M.-Z., Huo, J.-W., Deng, H.-X., and Zhang, X.-Z. (2015). A multifunctional metal-organic framework based tumor targeting drug delivery system for cancer therapy. Nanoscale *7*, 16061–16070.

66. Wang, J., Chen, D., Li, B., He, J., Duan, D., Shao, D., and Nie, M. (2016). Fe-MIL-101 exhibits selective cytotoxicity and inhibition of angiogenesis in ovarian cancer cells via downregulation of MMP. Sci. Rep. *6*, 26126.

67. Dong, Z., Sun, Y., Chu, J., Zhang, X., and Deng, H. (2017). Multivariate Metal-Organic Frameworks for Dialing-in the Binding and Programming the Release of Drug Molecules. J. Am. Chem. Soc. *139*, 14209–14216.

68. EFSA Panel on Food Additives and Nutrient Sources added to Food (ANS) (2010). Scientific opinion on the safety of trivalent chromium as a nutrient added for nutritional purposes to foodstuffs for particular nutritional uses and foods intended for the general population (including food supplements). EFSA J. *8*, 1882.

69. Wuttke, S., Zimpel, A., Bein, T., Braig, S., Stoiber, K., Vollmar, A., Müller, D., Haastert-Talini, K., Schaeske, J., Stiesch, M., et al. (2017). Validating Metal-Organic Framework Nanoparticles for Their Nanosafety in Diverse Biomedical Applications. Adv. Healthc. Mater. *6*, 1600818.

70. Latifi, L., and Sohrabnezhad, S. (2020). Drug delivery by micro and meso metal-organic frameworks. Polyhedron *180*, 114321.

71. Liu, C.-H., Chiu, H.-C., Sung, H.-L., Yeh, J.-Y., Wu, K.C.W., and Liu, S.-H. (2019). Acute oral toxicity and repeated dose 28-day oral toxicity studies of MIL-101 nanoparticles. Regul. Toxicol. Pharmacol. *107*, 104426.

72. Abánades Lázaro, I., Haddad, S., Sacca, S., Orellana-Tavra, C., Fairen-Jimenez, D., and Forgan, R.S. (2017). Selective Surface PEGylation of UiO-66 Nanoparticles for Enhanced Stability, Cell Uptake, and pH-Responsive Drug Delivery. Chem *2*, 561–578.

73. Orellana-Tavra, C., Marshall, R.J., Baxter, E.F., Lázaro, I.A., Tao, A., Cheetham, A.K., Forgan, R.S., and Fairen-Jimenez, D. (2016). Drug delivery and controlled release from biocompatible metal-organic frameworks using mechanical amorphization. J. Mater. Chem. B Mater. Biol. Med. *4*, 7697–7707.

74. Orellana-Tavra, C., Baxter, E.F., Tian, T., Bennett, T.D., Slater, N.K.H., Cheetham, A.K., and Fairen-Jimenez, D. (2015). Amorphous metal-organic frameworks for drug delivery. Chem. Commun. (Camb.) *51*, 13878–13881.

75. Abánades Lázaro, I., Haddad, S., Rodrigo-Muñoz, J.M., Orellana-Tavra, C., Del Pozo, V., Fairen-Jimenez, D., and Forgan, R.S. (2018). Mechanistic Investigation into the Selective Anticancer Cytotoxicity and Immune System Response of Surface-Functionalized, Dichloroacetate-Loaded, UiO-66 Nanoparticles. ACS Appl. Mater. Interfaces *10*, 5255–5268.

76. Bernini, M.C., Fairen-Jimenez, D., Pasinetti, M., Ramirez-Pastor, A.J., and Snurr, R.Q. (2014). Screening of bio-compatible metal-organic frameworks as potential drug carriers using Monte Carlo simulations. J. Mater. Chem. B Mater. Biol. Med. *2*, 766–774.

77. Bueno-Perez, R., Martin-Calvo, A., Gómez-Álvarez, P., Gutiérrez-Sevillano, J.J., Merkling, P.J., Vlugt, T.J.H., van Erp, T.S., Dubbeldam, D., and Calero, S. (2014). Enantioselective adsorption of ibuprofen and lysine in metal-organic frameworks. Chem. Commun. (Camb.) *50*, 10849–10852.

Cell Reports Physical Science, Volume *1*

Supplemental Information

Identifying Differing Intracellular Cargo Release Mechanisms by Monitoring *In Vitro* Drug Delivery from MOFs in Real Time

Panagiota Markopoulou, Nikolaos Panagiotou, Aurelia Li, Rocio Bueno-Perez, David Madden, Sarah Buchanan, David Fairen-Jimenez, Paul G. Shiels, and Ross S. Forgan

SUPPLEMENTAL EXPERIMENTAL PROCEDURES

General Experimental Remarks

Powder X-ray diffraction (PXRD): PXRD measurements were carried out at 298 K using a PANalytical X'Pert PRO diffractometer (λ (CuKα) = 1.4505 Å) on mounted bracket sample stage. Data were collected over the range 3-45˚. PXRD patterns were predicted from single crystal data using Mercury 4.0.[S1]

Thermogravimetric analysis (TGA): Measurements were carried out using a TA instruments Q500 Thermogravimetric Analyser. Measurements were collected from room temperature to 800˚C with a heating rate of 10˚C / min under an air atmosphere. Data were analysed with TA Instruments Universal Analysis 2000.

Gas uptake: For UiO-66 samples, N_2 adsorption isotherms were carried out at 77 K on a Quantachrome Autosorb IQ gas sorption analyser. Samples were degassed under vacuum at 393 K for 20 h on the instrument prior to analysis. For MIL-101(Cr) samples, N_2 isotherms were collected using a Micromeritics 3Flex at 77 K. Prior to analysis, samples were degassed in a vacuum oven at 393 K for 12 h. In situ degas (393 K, 12 h) was further performed after sample loading into the instrument,

Pore size distribution: : For UiO-66 samples, pore size distributions were calculated using the N_2 at 77 K on a carbon (slit pore, QSDFT, equilibrium model) calculation model within the Quantachrome ASiQwin operating software. For MIL-101(Cr) samples, the N_2@77-Carb Cyl Pores, MWNT, NLDFT wthin the Micromeritics operating software was used.

Dynamic light scattering (DLS): Colloidal stability analysis was performed by DLS with a Zetasizer Nano ZS potential analyser equipped with non-invasive Backscatter optics (NIBS) and 50 mW laser at 633 nm. Data were analysed with Zetasizer Nano software v3.30.

Scanning electron microscopy (SEM): The powder samples were deposited onto carbon tabs and coated with Pd for 150 seconds using Polaron SC7640 sputter coater and imagined using a Carl Zeiss Sigma Variable Pressure Analytical SEM with Oxford Microanalysis. Particle size distribution was analysed manually using ImageJ v1.52 software.

Fourier transform infrared spectroscopy (FT-IR): Infrared spectra of solids were collected using Schimadzu Fourier Transform Infrared Spectrometer, FTIR-8400S, fitted with a Diamond ATR unit.

UV-Vis spectroscopy: UV-Vis spectra were recorded using a Schimadzu UV-1800 and analysis was carried out using the software UVProbe v2.51. In some cases, a ThermoFischer Nanodrop One was used (stated) and data were analysed with Graphpad Prism 7.

Real time cell analysis (RTCA): RTCA cell proliferation assays were performed on an xCELLigence® RTCA MP machine, with E-plate VIEW 96. The RTCA normalised cell index over time data, which were collected, were analysed with the use of the RTCA software version 1.2.1 (ACEA Biosciences Inc.).

Plate reader: The UV-Vis spectrometry and fluorescence intensity experiments for the biological assays were performed on a CLARIOstar plate reader (BMG LABTECH). Data were analysed with Graphpad Prism 7.

Flow cytometry: All the flow cytometry experiments were carried out with an Attune NxT (Thermo Fischer). Excitation laser 488 nm, configuration filters FITC 530/30 nm and PerpCP-Cy5.5 695/40 nm. The analysis was carried out with FCS express 6 software.

Confocal microscopy: For confocal microscopy, a ZEISS LSM 780 Confocal microscope was employed. Images were captured with the aid of ZEN black software. Analysis was performed with the custom Fiji macro BatchQuantify software.[S2]

MOF Synthesis and Characterisation

All solvents and reagents were purchased from Alfa Aesar, Acros Organics, Sigma-Aldrich, Merck, Tokyo Chemical Industry, Thermo Fischer Scientific and Zymo Research USA, and used without further purification. All MOFs were dried *in vacuo* at room temperature for at least 24 hours and subsequently in the oven at 120 ˚C overnight before any characterisation or further experiments took place.

Synthesis of UiO-66: Zirconium (IV) chloride (0.6839 g, 3 mmol) was dissolved in 30 mL DMF (sonication until complete dissolution). In a separate container, terephthalic acid (0.4486 g, 3 mmol) was dissolved in 30 mL DMF. The two solutions were mixed in a glass pyrex jar, 4.2 mL (7% tot. vol.) acetic acid was added, and the jar was heated at 120 ˚C for 24 hours. The jar was allowed to cool down to ambient temperature, the nanoparticles were collected by centrifugation (4500 rpm for 20 minutes), washed with DMF (×2) and MeOH (×3) (sonication and centrifugation cycles) and were dried *in vacuo* for 72 hours. This synthesis was adapted from a literature source.[S3]

Synthesis of MIL-101(Cr): Chromium (III) nitrate nonahydrate (1.6 eq, 8 mmol) was dissolved in 25 mL deionised water (sonication until complete dissolution). Then, terephthalic acid (1 eq, 5 mmol) was added and the suspension was sonicated for another 10 minutes. Acetic acid (1 eq, 5 mmol) was added and the suspension was transferred into a Teflon lined steel autoclave bomb and was heated at 220 °C for 6 hours. The autoclave bomb was left to cool down to ambient temperature and the nanoparticles formed were collected with centrifugation and dried *in vacuo* for 24 hours. The solid was loaded into a 100 mL round bottom flask and 50 mL DMF were added. The suspension was refluxed under stirring at 140 °C for 4 hours. Then it was centrifuged and dried *in vacuo* overnight. The nanoparticles were washed once with DMF and once with MeOH (sonication and centrifugation cycles) and were dried *in vacuo* for 72 hours. This synthesis was adapted from a literature source.[S4]

Powder X-Ray Diffraction (PXRD): The first part of characterisation of the materials involved verifying their crystallinity and validating their identity. Both goals were achieved with powder X-ray diffraction (PXRD). **Figure S1** shows the PXRD patterns of the materials compared to the calculated patterns based on the crystal structures found in the Cambridge Crystallographic Data Centre (CCDC) database. The high crystallinity of all the samples is prominent and a very close match of the experimentally acquired patterns to the calculated ones is obvious in both cases.

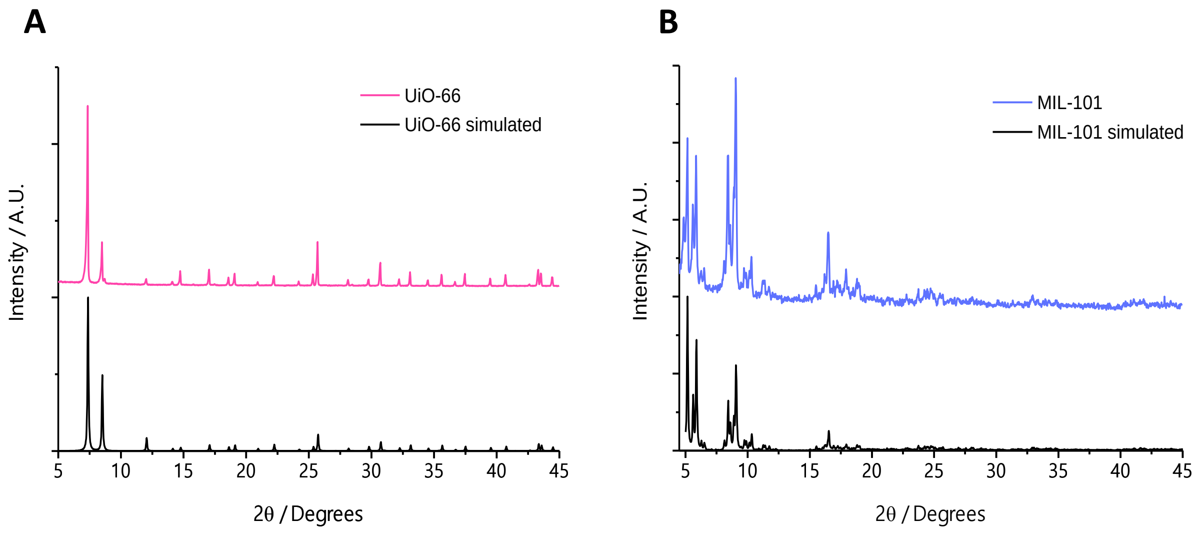

Figure S1: PXRD patterns of the as synthesised MOFs compared to their corresponding calculated patterns. (A) UiO-66. (B) MIL-101(Cr).

Thermogravimetric Analysis (TGA): The thermal stability of the materials was tested with TGA in air. As expected, UiO-66 was the sample with the higher thermal stability with a thermal decomposition temperature of 534 °C, which also matches the thermal stability reported for this material in the literature.[S3] There was not a mass loss step before 200 °C, which indicated that there were no residues of the synthesis or washing solvents. A small mass loss step was present at 250–300°C. This is characteristic for the UiO family MOFs and is attributed to the dehydration of the zirconium oxoclusters, $[Zr_6O_4(OH)_4L_6]$ (where L=ligand) losing two water molecules and adopting the formation $[Zr_6O_6L_6]$.[S5] The thermal decomposition of MIL-101(Cr) started at approximately 345 °C. Cr^{3+} MOFs are generally very chemically stable as well, due to Cr^{3+} being a hard Lewis acid and therefore having a favourable stable bond with the hard Lewis basic groups of the carboxylate linker. A mass loss step of 7.5% was present at a lower temperature. This was due to evaporation of adsorbed ambient moisture **(Figure S2)**. This mass loss step could not correspond to residual DMF (synthesis solvent) as it begins at ambient temperature which is very low for DMF evaporation. The chance for this step corresponding to residual methanol (washing solvent) was also eliminated as the material was thoroughly dried *in vacuo* and at 120 °C before the characterisation took place. However, the sample was not stored *in vacuo* or under dry conditions and therefore ambient moisture could be adsorbed. This was also confirmed by FT-IR spectroscopy where the characteristic peak of absorbed water is present at 3250 cm^{-1} **(Figure S3)**.

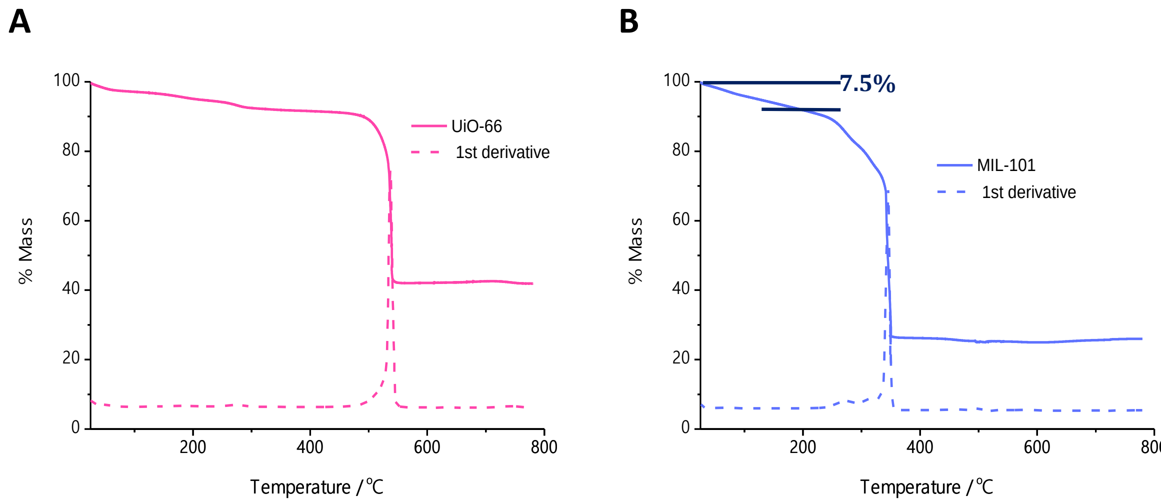

Figure S2: TGA profiles and first derivative. (A) UiO-66 and (B) MIL-101(Cr).

FT-IR: Further characterisation of the materials was achieved with solid state FT-IR. from the FT-IR spectra, the absence of solvent traces was further validated. A wide peak at approximately 3000-3500 cm^{-1} is present, this corresponds to adsorbed water on the MOF structure **(Figure S3)**.

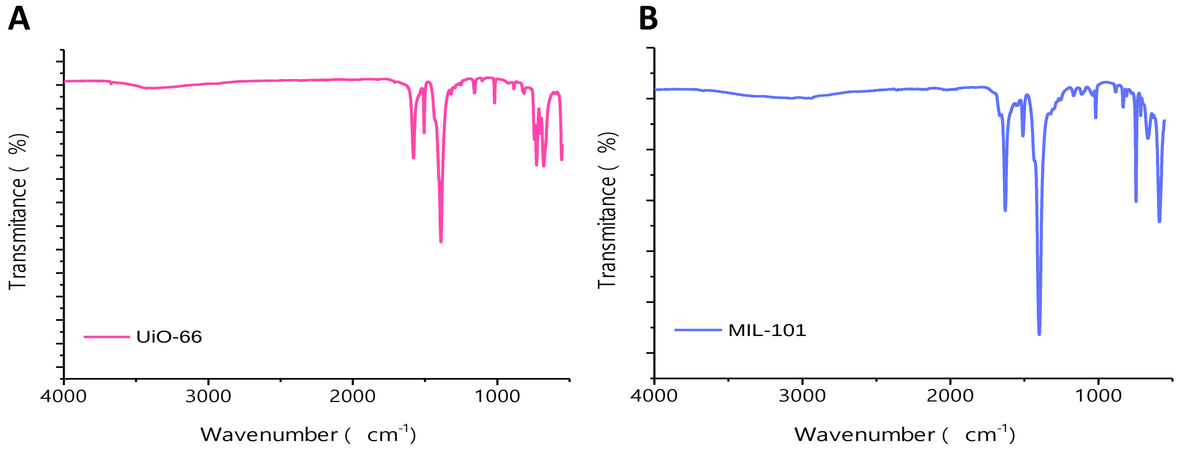

Figure S3: Solid state FT-IR spectra. (A) UiO-66 and (B) MIL-101(Cr).

SEM: The size and surface morphology of the synthesised materials was studied with SEM. The average diameter of the nanoparticles was calculated manually with the aid of ImageJ software. The diameter of an average of 200 nanoparticles was measured for each material and bins of 20 nm were used. For both materials, a characteristic octahedral shape was observed and the average particle dimeter for UiO-66 was 391±103 nm and for MIL-101(Cr) was 188±47 nm **(Figure S4)**.

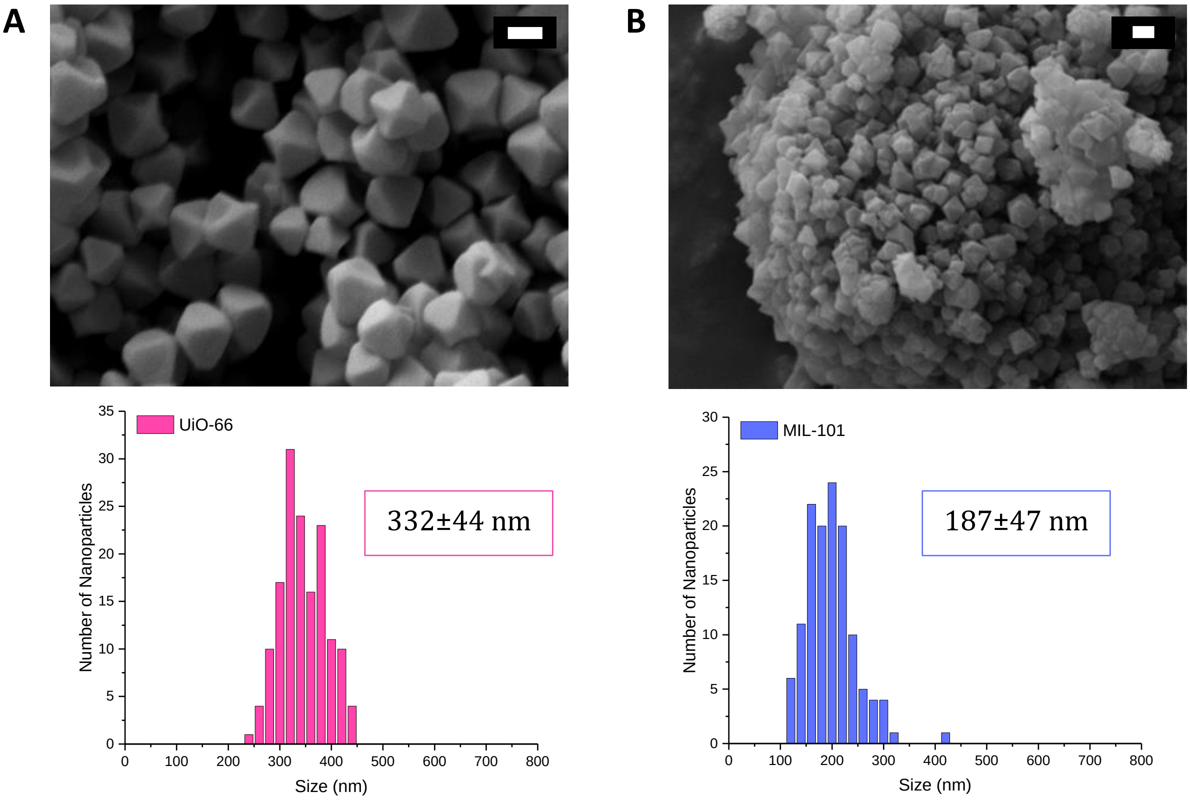

Figure S4: SEM images of (A) UiO-66 and (B) MIL-101(Cr). Scale bars represent 200 nm. Diameter size distribution graphs and average diameter size of (A) UiO-66 (PDI = 0.380) and (B) MIL-101(Cr) (PDI = 0.462) particles. Data represented as mean ± SD.

Dynamic Light Scattering (DLS): The hydrodynamic radius of the nanoparticles was also measured with DLS. In the case of UiO-66, minimal aggregation was observed between the three measurements. This was not significant however, as the intensity of the three consecutive measurements is not decreasing, as it is expected when serious aggregation occurs and the PDI value of the measurements was low (0.380). The average hydrodynamic radius of this material was 904 nm indicating that the colloidal suspension of the nanoparticles consists of small aggregates and not individual nanoparticles as their diameter was approximately 330 nm according to SEM. MIL-101(Cr) nanoparticles showed an interesting behaviour. Initially, during the first measurement, two populations were observed, one with a diameter of approximately 140 nm, which is very close to the diameter of individual nanoparticles according to SEM, and one of aggregates with diameter of approximately 955 nm. However, in the following measurements, the large hydrodynamic radius population disappeared. Moreover, the intensity of the population with small hydrodynamic radius increased, indicating that the nanoparticles were getting better suspended with potential large aggregate clusters breaking apart and reaching equilibrium at aggregates with lower hydrodynamic size of approximately 369 nm. No further aggregation of the material was observed which along with the low PDI of the measurement suggest that the material forms a stable colloidal suspension **(Figure S5)**.

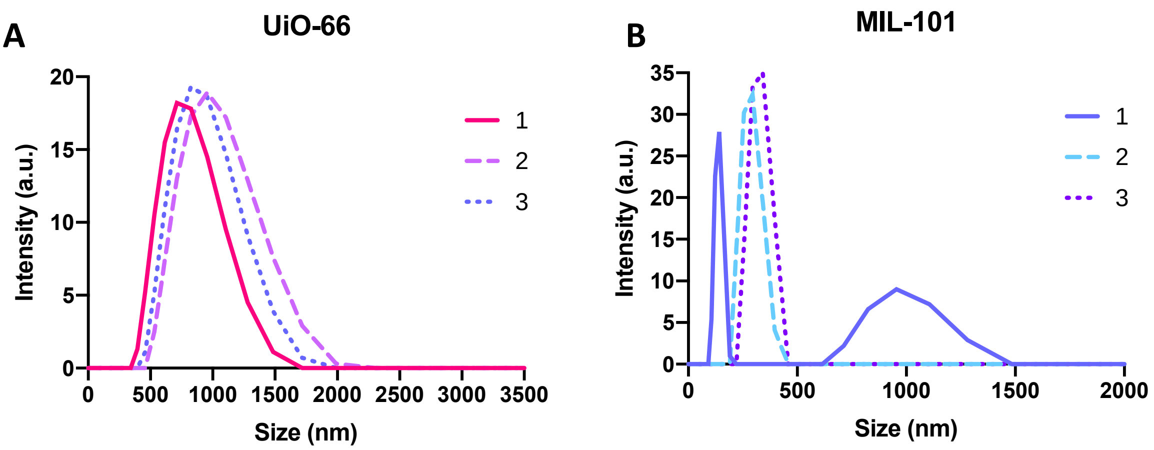

Figure S5: Three consecutive DLS measurements of MOF nanoparticles suspended in PBS 1X pH 7.4 spiked with 2% v/v FBS. (A) UiO-66 and (B) MIL-101(Cr).

Zeta potentials in deionized water and cell culture media were collected **(Figure S6)**, showing that while significant differences in the bare MOFs are evident, on suspension in media a protein corona formed to yield similar surface charge for both MOFs.[S6-S8]

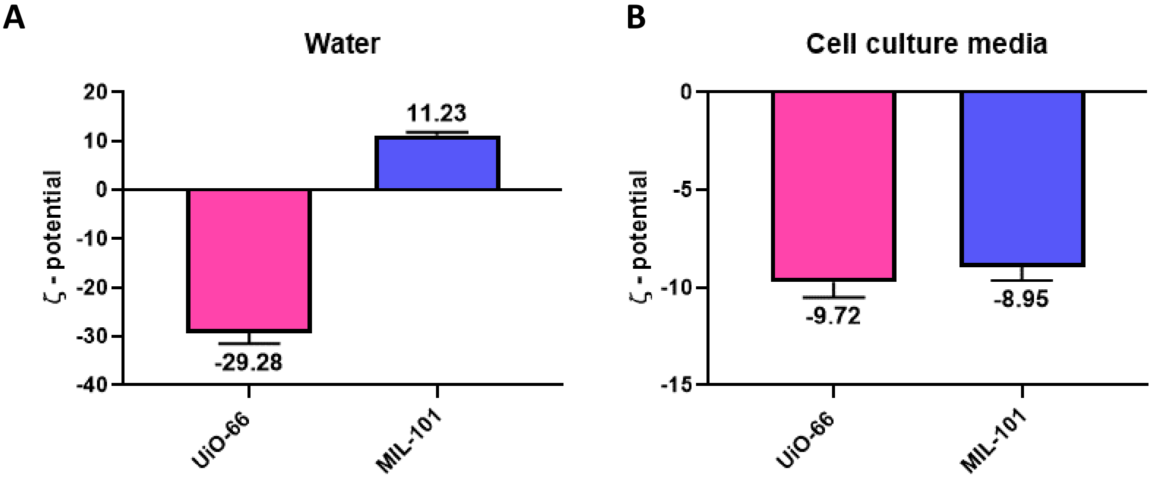

Figure S6: Surface ζ-potential measurements of MOF nanoparticles suspended in (A) water and (B) cell culture media.

In Vitro Biocompatibility Assays

Cell culture: All cell culture procedures were performed under sterile conditions. The cells that were used were cultured in Corning® T75 flasks at 37 ˚C, in a humidified incubator atmosphere maintained at 5% CO_2. For passaging, the cells were washed twice with PBS 1X and trypsinised at 37 °C for 7 minutes with Trypsin-EDTA (0.25%). Complete media in a 3:1 ratio was used to deactivate the effect of Trypsin, which was removed by centrifugation, and then the cells were seeded in new Corning® T75 flasks. The corresponding cell growth media used for each cell line as well as the seeding ratios can be found in **Table S1**.

Table S1: Cell growth media used for each cell line and their passaging seeding ratios.

<table>
<tr><th>Cell type</th><th>Media and supplements</th><th>Seeding ratio</th></tr>
<tr><td>HDF
(primary)</td><td>DMEM with GlutaMAX™-I and 10% (v/v) heat inactivated FBS, 100 units mL^{-1} penicillin, 100 units mL^{-1} streptomycin, 1% Amphotericin B</td><td>1:3</td></tr>
<tr><td>HEK-293
(ECACC 85120602)</td><td rowspan="2">DMEM-high glucose and 10% (v/v) heat inactivated FBS, 2 mM L-glutamine, 100 units mL^{-1} penicillin, 100 units mL^{-1} streptomycin</td><td>1:10</td></tr>
<tr><td>MCF-7
(ECACC 86012803)</td><td>1:5</td></tr>
<tr><td>A2780ADR
(ECACC 93112520)</td><td>RPMI-1640 and 10% (v/v) heat inactivated FBS, 2 mM L-glutamine, 100 units mL^{-1} penicillin, 100 units mL^{-1} streptomycin</td><td>1:5</td></tr>
<tr><td>HepG2
(ECACC 85011430)</td><td>MEM and 10% (v/v) heat inactivated FBS, 2 mM L-glutamine, 100 units mL^{-1} penicillin, 100 units mL^{-1} streptomycin, 1% (v/v) non-essential amino acids solution (100X)</td><td>1:5</td></tr>
</table>

RTCA: RTCA assays require the use of xCELLigence® RTCA instrument and E-plate VIEW 96 well plates. These plates are covered with gold microelectrodes at the bottom of the wells and an electrical signal generated by the instrument is allowed to pass through the electrodes. The presence of cells disrupts and delays the electrical signal. Hence, the disruption of the signal correlates very well with the number of cells present. By measuring this delay, we can estimate cell index, which reflects the number of cells present at a given point. Measuring cell index overtime allows us to observe cell proliferation, cell loss or cytostatic effects, as well as cell morphology. Control experiments showed that the MOFs themselves did not interfere with the impedance measurements (Figure S7). 100 µL complete media per well were added to the E-plate VIEW 96 and it was incubated at room temperature for 10 minutes. The plate was added to the RTCA instrument in the incubator (37˚C, 5% CO_2), to measure background impedance. The cells were counted and added to the plate at a concentration of 5×10^3 cells per well to a total volume of 200 µL per well. The plate was returned to the RTCA instrument and the cells were allowed to sediment for 30 minutes before the start of the measurement. After 24 hours of incubation, the media were carefully aspirated. The MOF nanoparticles were suspended in complete media and were sonicated for 5

minutes in the various concentrations. The MOF suspension treatment was added (200 µL per well) - 6 technical replicates for each concentration. Complete media were added in the untreated controls (200 µL per well) – 6 technical replicates. Typically, the addition of material and agitation results in a temporary small drop in cell index, which recovers quickly. The plate was returned to the RTCA instrument where cell growth was recorded for a further 72 hours. Overall, 3 biological replicates were performed. The RTCA traces for an indicative biological repeat using human dermal fibroblasts (HDFs) are shown in the main manuscript **(Figures 1A and 1B)** and for MCF-7 and HEK-293 cells in **Figure S8**. The slope analyses in **Figures 1C–1E** of the main manuscript combine all biological replicates.

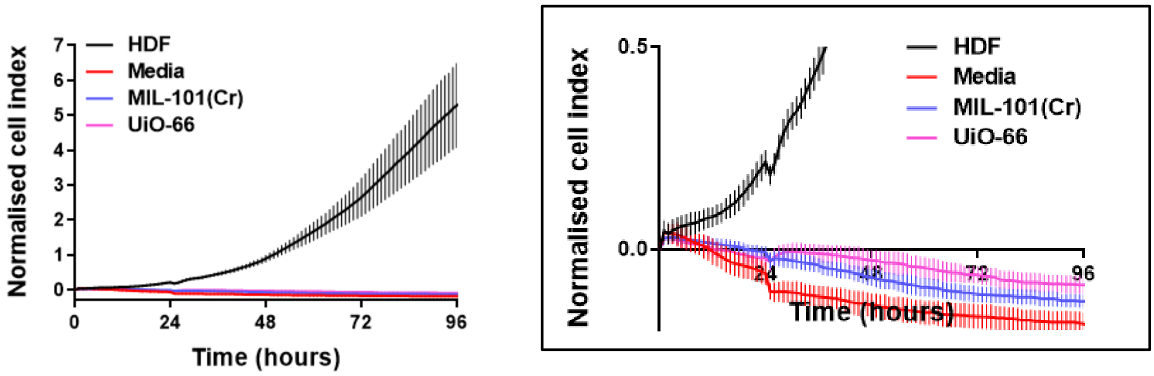

Figure S7: MOF addition does not increase cell index in the absence of HDF cells (right – inset).

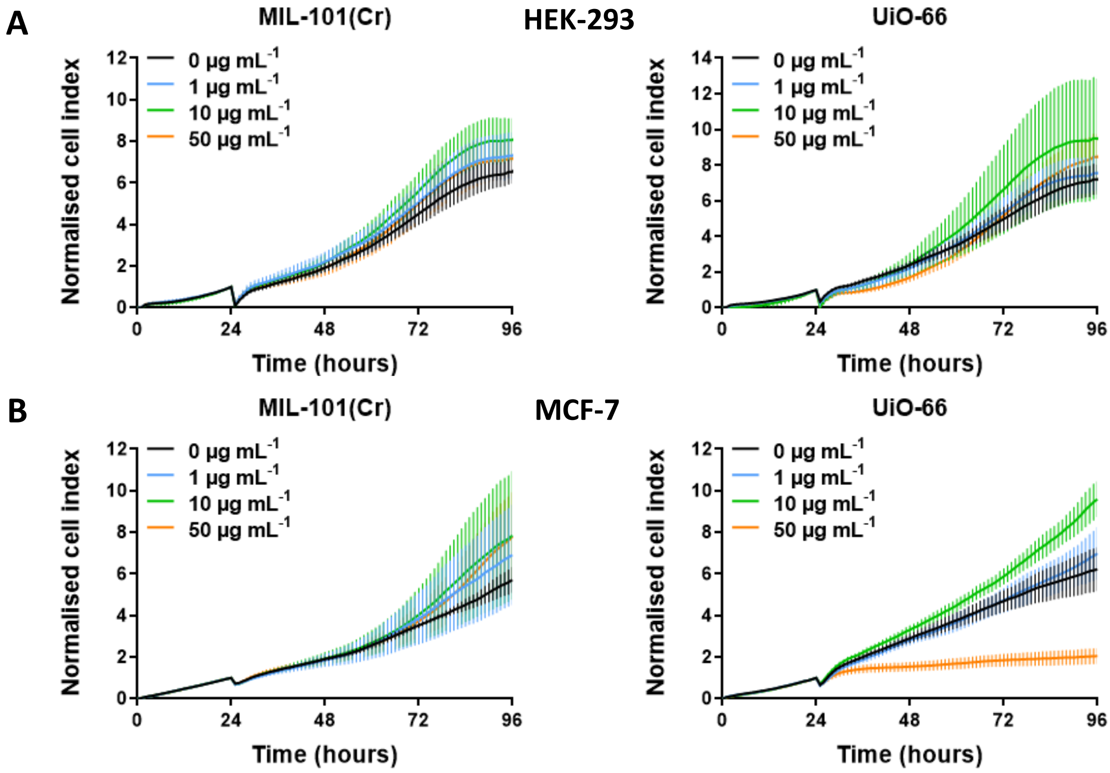

Figure S8: Biocompatibility assessment of MOFs. Real time cell analysis screening of MIL-101(Cr) and UiO-66 in (A) HEK-293 and (B) MCF-7 cells.

Alamar blue assays: Cells were seeded into a VWR treated 96 well plate at a density of 5×10^3 cells per well (200 µL media per well). The cells were incubated for 24 hours in a humidified incubator atmosphere maintained at 37 ˚C and 5% CO_2. The MOF nanoparticles were suspended in complete media and were sonicated for 5 minutes in the various concentrations. The media were aspirated from the cells and the MOF suspension treatment was added (200 µL per well) - 3 to 6 technical replicates for each concentration. Complete media were added in the untreated controls (200 µL per well) - 6 technical replicates. The cells were incubated for 24 or 72 hours in a humidified incubator atmosphere maintained at 5% CO_2. To measure the metabolic activity, 20 µL per well Alamar Blue™ Cell Viability Reagent were added and the cells were incubated for 3.5-5 hours (depending on the cell line) in a humidified incubator atmosphere with 5% CO_2. Then the fluorescence intensity was measured on the plate reader (Exc 557±10 nm, Ems 593±10 nm). Overall, 3 biological replicates were performed. The data are presented in **Figures 1F–1H** of the main manuscript.

Calcein Loading and Characterisation

Synthesis of Cal@MOFs: The MOF nanoparticles **(Table S2)** were suspended in a calcein solution in ethanol/water 1:1 *v/v* at a concentration of 1 $mgmL^{-1}$. The suspension was sonicated for 10 minutes and then was left stirring at room temperature for 96 hours. The nanoparticles were isolated with centrifugation (4500 rpm, 20 min) and were washed with ethanol until the supernatant was clear. For calcein loading determination the nanoparticles were degraded in PBS 1X pH 5.5 for 5 days and their corresponding calcein content was calculated with UV-Vis spectroscopy with the use of a calibration curve.

Table S2: MOF mass and calcein solution volume.

MOF	Mass (g)	Calcein solution volume (mL)
UiO-66	0.06709	30
MIL-101(Cr)	0.05177	25

The successful calcein staining was visually identified by the colour change of the materials from white to orange-yellow for UiO-66 and from green to brown for MIL-101(Cr) **(Figure S9)**.

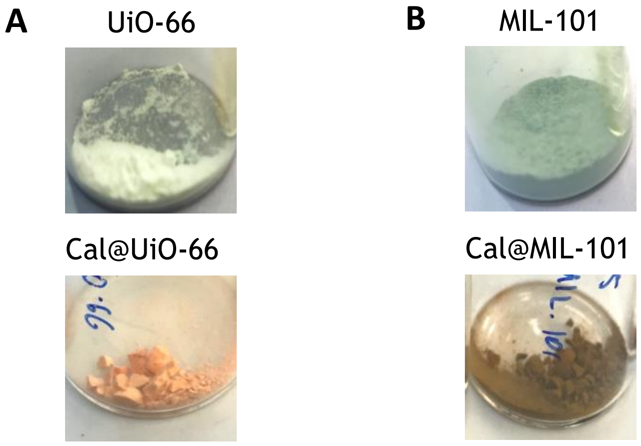

Figure S9: Colour change of the MOF nanoparticle powders after calcein staining. (A) UiO-66 and Cal@UiO-66. (B) MIL-101(Cr) and Cal@MIL-101(Cr).

The calcein loaded materials were highly crystalline and their structure was not altered as a very similar pattern to the as synthesised MOFs was obtained by PXRD **(Figure S10)**.

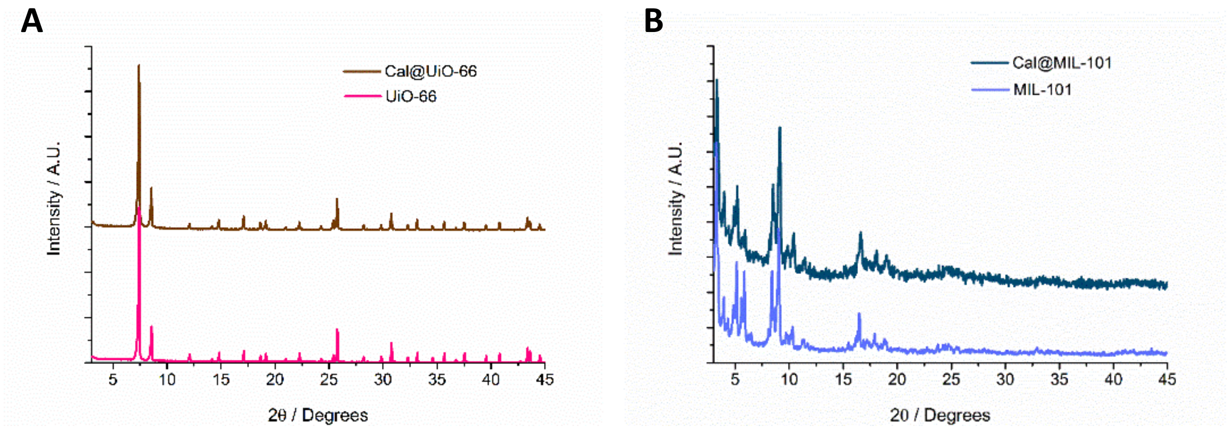

Figure S10: PXRD patterns. (A) UiO-66 and (B) MIL-101(Cr) nanoparticles before and after calcein staining.

The calcein stained materials had a very similar thermal stability as their “empty” precursors. This, along with the crystallinity of the materials, further confirms that the MOF structure was not compromised during calcein loading. The 100% mass of each material was normalised to 150°C to avoid taking solvent evaporation into account of organic mass loss. A small organic mass addition is observed indicating the successful incorporation of calcein to the MOF structure **(Figure S11)**.

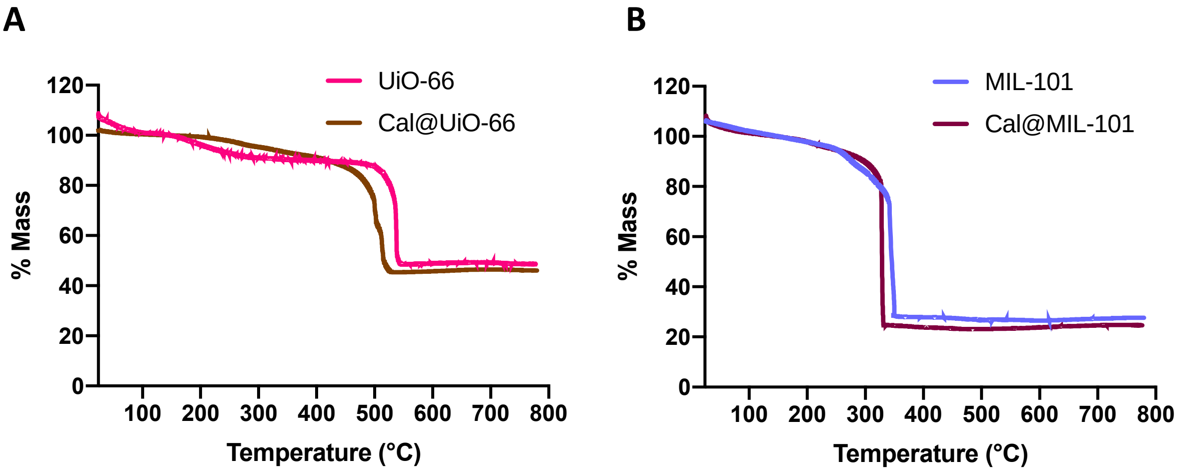

Figure S11: Thermogravimetric analysis profiles. (A) UiO-66 and (B) MIL-101(Cr) nanoparticles before and after calcein staining.

The calcein content of each MOF was calculated quantitatively by UV-Vis spectroscopy. The calcein stained nanoparticles were suspended in PBS 1X pH 5.5 in a concentration of 0.5 $mgmL^{-1}$ with sonication for 10 minutes. The suspension was left stirring at room temperature until the solid residue was not stained orange (5-8 days). Then, the solid was removed with centrifugation and the calcein content of the nanoparticles was calculated based on the absorbance of the supernatant with the use of the calibration curve in **Figure S12** and using equation **S1**. The exact calcein content of each material can be found in **Table S3**.

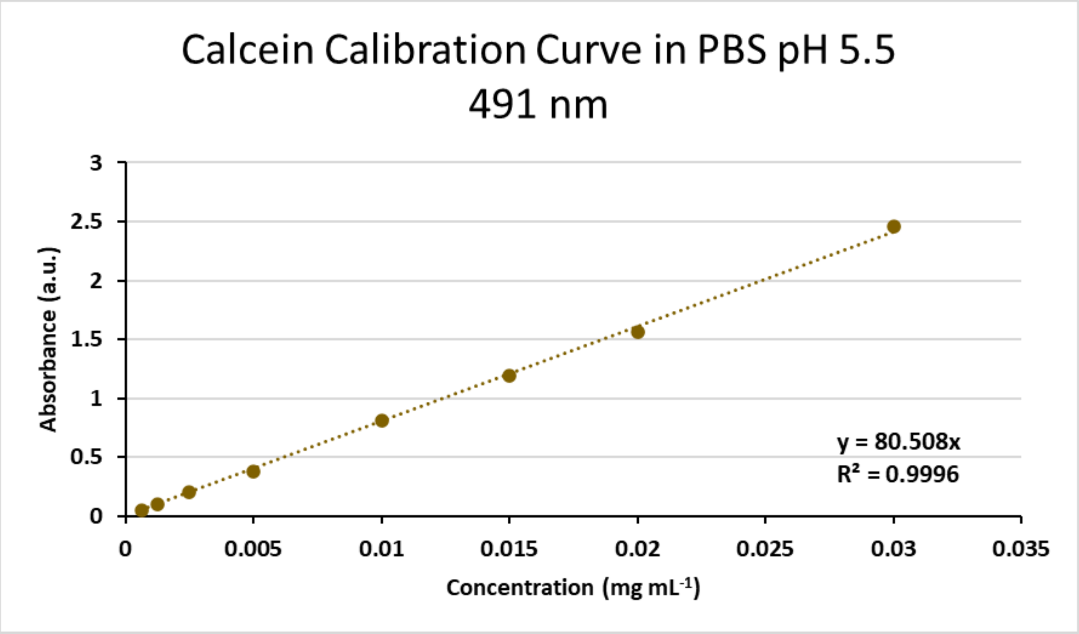

Figure S12: Calcein calibration curve in PBS 1X pH 5.5 based on absorbance at 491 nm.

$$\text{Calcein Content } (\%\, w/w) = \frac{\text{Loaded calcein mass}}{\text{MOF initial mass}} \times 100\% = \frac{\text{Abs@491 nm}/80.508}{0.5 \text{ mgmL}^{-1}} \times 100\% \quad \textbf{(S1)}$$

Table S3: Calcein content of each stained MOF.

MOF	Calcein content (*wt%*)
Cal@UiO-66	4.8
Cal@MIL-101(Cr)	6.7

Internalisation and Biocompatibility by Flow Cytometry

Flow cytometry: Cells were seeded into a VWR treated 96 well plate at a density of 5×10^3 cells per well (200 µL media per well). The cells were incubated for 24 hours in a humidified incubator atmosphere maintained at 37 ˚C and 5% CO_2. The MOF nanoparticles were suspended in complete media and were sonicated for 5 minutes in the various concentrations. The media were aspirated from the cells and the MOF suspension treatment was added (200 µL per well) – 3 to 6 technical replicates for each concentration. Complete media were added to the untreated controls (200 µL per well) – 6 technical replicates. The cells were incubated for 24 or 72 hours in a humidified incubator atmosphere maintained at 37 ˚C and 5% CO_2. The media were carefully aspirated and 50 µL per well Trypsin-EDTA (0.25%) were added. The plate was incubated (37 ˚C, 5% CO_2) for 5 minutes. 150 µL per well fresh media were added and the cells were resuspended by pipetting and transferred to a Corning® 96 round bottom well plate. Then, the cells were centrifuged at 1200 rpm for 10 minutes. The supernatant media was removed by flipping the plate over a piece of tissue paper. 200 µL of the Flow Buffer (PBS 1X, 2% FBS and 2 mM EDTA diluted in PBS 1X) per well were added with SYTOX™ AADvanced™ Dead Cell Stain at a concentration of 1 µL of 1 mM stain per mL of Flow Buffer. In the samples of untreated cells and FITC channel control used for the compensation of the instrument, the Flow Buffer solution added did not contain the SYTOX™ AADvanced™ Dead Cell Stain. For the sample used for the PeprCp-Cy5.5 channel control used for the compensation, 160 µL of the Flow Buffer containing the SYTOX™ AADvanced™ Dead Cell Stain and 40 µL of DMSO were added per well. Overall, 3 biological replicates were performed using the gating strategy in **Figure S13**.

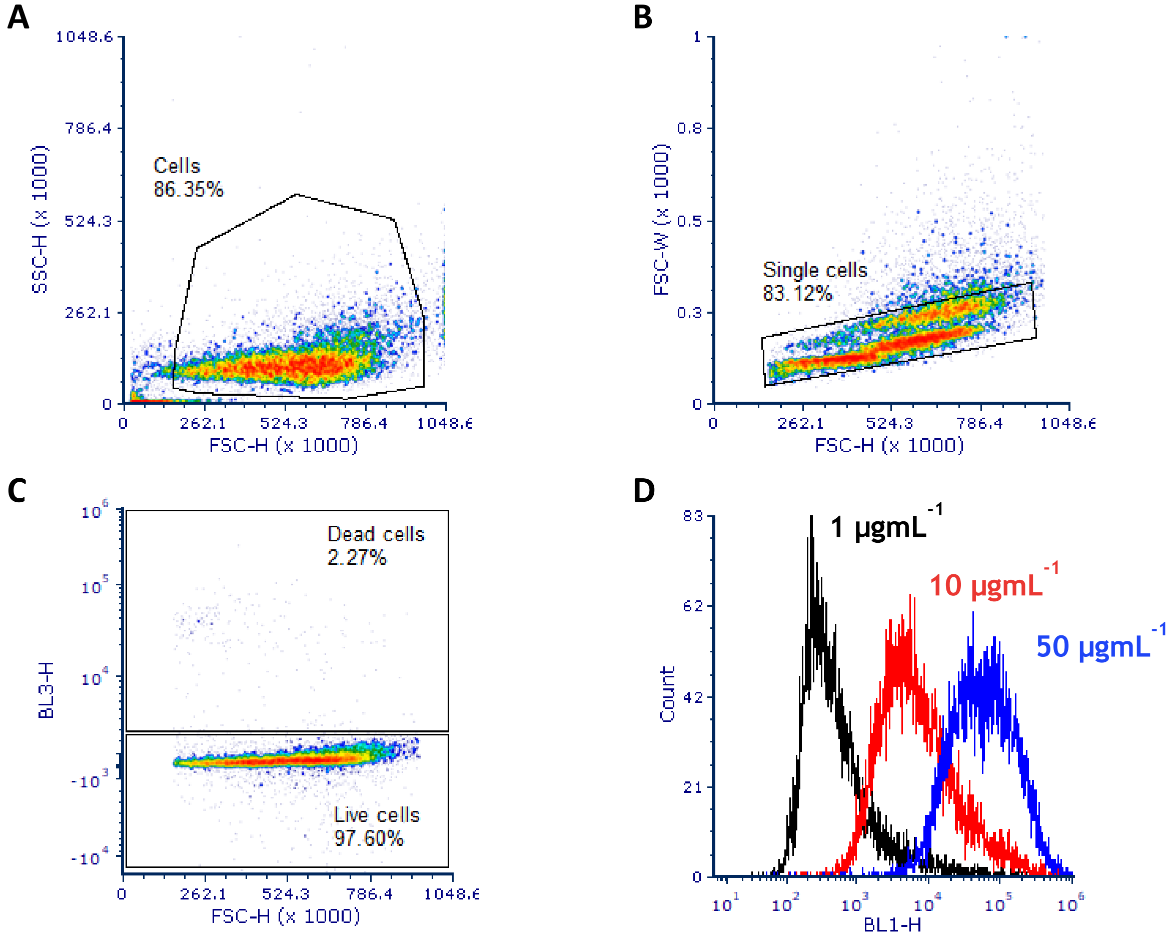

Figure S13: Gating strategy. (A) Gating of cells. (B) Gating of single cells. (C) Gating of live and dead cells. (D) Fluorescence intensity histogram of calcein stained live cells, with representative example of dose depended cytoplasmic fluorescence intensity increase after treatment with increasing concentrations of Cal@MIL-101(Cr).

The cell viability measurements were based on the absolute number of data points in the “live cells” gate instead of the percentage. This is due to the fact that the percentage represents “live cells” as a fraction of the total “cells” gate data points. In the case however where a treatment is causing a cytotoxic effect, the total “cells” data points are significantly less compared to the untreated control. This however does not necessarily mean that the percentage of “live cells” in the sample, as a fraction of the total “cells” number, is going to be significantly lower compared to the untreated control. Moreover, it needs to be noted that before running the experiment in the flow cytometer, a series of multiple preparation steps take place. During these steps, already dead cells are removed as they are no longer adherent to the bottom of the wells and this results in samples with significantly different total “cells” populations having similar percentages of “live cells”. Hence absolute numbers were used for the cell viability analysis.

Doxorubicin Loading and Characterisation

Synthesis of Dox@MOFs: 0.1 g doxorubicin was dissolved in 60 mL Tris buffered saline (sonication until complete dissolution). The MOF nanoparticles **(Table S4)** were added and the suspension was sonicated at room temperature for ten minutes and then was left stirring at room temperature for 5 days protected from light. The doxorubicin loaded nanoparticles **(Figure S14)** were isolated with centrifugation (4500 rpm for 20 minutes) and washed with Tris buffered saline until the supernatant was colourless. The supernatant loading solution and all the washings were collected and diluted up to 500 mL with Tris buffered saline. The drug loading capacity mass percentage (DLC% w/w) was calculated based on the concentration difference of the drug solution before and after the loading. This was done with UV-Vis spectroscopy with the use of a calibration curve. The drug loaded nanoparticles were dried *in vacuo* for 48 hours and then at 120 °C overnight before any characterisation or further experiments took place.

Table S4: MOF weight and doxorubicin solution volume.

MOF	Weight (g)	Doxorubicin solution volume (mL)
**UiO-66**	**0.0055**	**60**
**MIL-101(Cr)**	**0.0070**	**60**

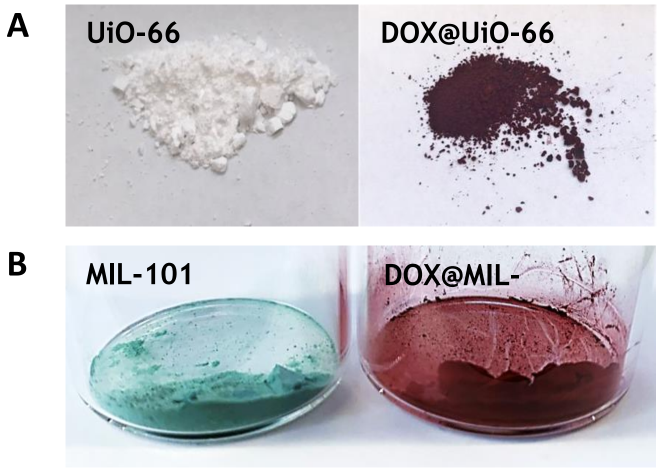

Figure S14: Colour change of the MOF nanoparticle powders after doxorubicin loading. (A) UiO-66 and Dox@UiO-66. (B) MIL-101(Cr) and Dox@MIL-101(Cr).

The mass-based drug loading capacity (DLC% wt) of each MOF was calculated based on the doxorubicin supernatant solution concentration difference before and after the loading. The doxorubicin concentration was calculated by UV-Vis spectroscopy with the use of the calibration curve in **Figure S15**.

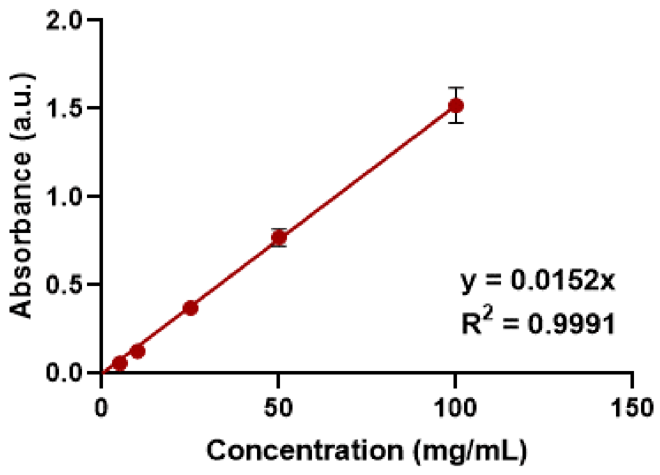

Figure S15: Doxorubicin calibration curve in Tris Buffered Saline pH 7.4 based on absorbance at 481 nm. Data represented as mean ± SD (n=3).

The wt% drug loading capacity of the materials was calculated based on grams of drug per grams of loaded material, using equation **S2**, and were 58% wt for UiO-66 and 10% wt for MIL-101(Cr).

$$\text{DLC\% wt} = \frac{\text{weight of loaded doxorubicin}}{\text{weight of loaded doxorubicin + MOF initial weight}} \times 100\% \quad \textbf{(S2)}$$

By TGA analysis, an increase of the organic content was obvious in both cases, indicative of successful drug loading **(Figure S16)**. For Dox@UiO-66, thermal degradation was observed at a lower temperature than the non-loaded MOF. This is a general trend that has been observed for surface functionalised MOFs.67,68 It is hypothesised that, due to the added organic surface functionality having a lower thermal stability, combustion is initiated at a lower temperature and that triggers earlier thermal decomposition of the material as a whole. For Dox@MIL-101(Cr) however, thermal decomposition began almost at the same stage as MIL-101(Cr), but the overall MOF degradation occurred at a slightly higher temperature.

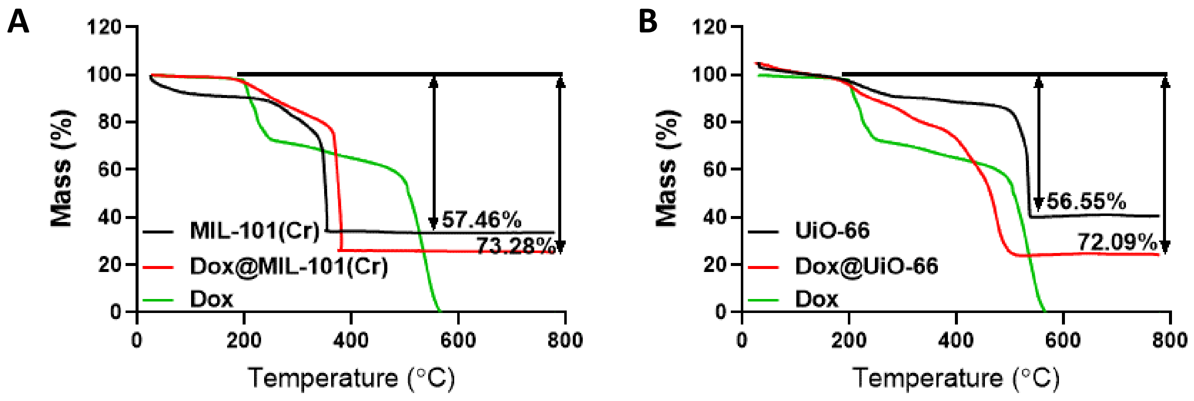

Figure S16: Thermogravimetric analysis profiles of **(G)** UiO-66 and **(H)** MIL-101(Cr), before and after Dox loading and compared to the profile of free Dox.

To computationally assess the different Dox uptakes in the two MOFs, we performed grand canonical Monte Carlo (GCMC) simulations of doxorubicin in UiO-66 and MIL-101(Cr) at 310 K with the multi-purpose code RASPA.[S9] We used an atomistic model of UiO-66 and MIL-101(Cr) for which the framework atoms were kept fixed at the crystallographic positions. We used the standard Lennard-Jones (LJ) 12-6 potential to model the Van der Waals interactions, using Lorentz-Berthelot mixing rules to define the interactions between the framework and adsorbate atoms, and a Coulomb potential to describe the electrostatic interactions. The parameters for the framework atoms **(Table S5)** were derived from the Universal Force Field[S10] and the Dreiding Force Field.[S11] Doxorubicin was modelled with the OPLS-AA forcefield **(**

Table S*6*),[S12] as a flexible model **(Figure S17)**, except for the rings that were kept rigid. The bonds, bends and torsion definitions used in RASPA for this molecule are described in

Table S7. EQeq was used to assign the partial charges of the framework.[S13] LJ interactions beyond 12.8 Å were neglected. The Ewald sum method was used to compute the electrostatic interactions in the system. Up to 200,000 Monte Carlo cycles were performed, the first 10% of which were used for equilibration, and the remaining steps were used to calculate the ensemble averages. Monte Carlo moves consisted of insertions, deletions, displacements, and rotations. In a cycle, *N* Monte Carlo moves are attempted, where *N* is defined as the maximum of 20 or the number of adsorbates in the system.

Table S5: Force field parameters for MIL-101 (Cr) and UiO-66.

**Atom label**	**$\varepsilon\ k_B^{-1}$ / K**	**σ / Å**	**Average charge (e^-)**
**MIL-101(Cr)**			
Cr	7.54829	2.69319	1.529
C1	47.8562	3.47299	0.496
C2	47.8562	3.47299	-0.062
O1	48.1581	3.03315	-0.853
O2	48.1581	3.03315	-0.574
O3	48.1581	3.03315	-0.438
F	36.4834	3.0932	-0.547
H	7.64893	2.84642	0.108
**UiO-66**			
Zr	34.7221	2.78317	3.197
C	47.8562	3.47299	0.103
O	48.1581	3.03315	-0.844
H	7.64893	2.84642	0.0492

Table S6: Force field parameters for doxorubicin.

Atom label	Atom type	ε k_B^{-1} / K	σ / Å	Charge (e^-)
C_ARO	CA	35.22	3.55	-0.115
C_ANI	CA	35.22	3.55	0.085
C_ANIt	CA	35.22	3.55	0.170
C_NAF	CA	35.22	3.55	0.0
C_KET	C_2	52.83	3.75	0.470
C_FEN	CA	35.22	3.55	0.150
C_PET	CT	33.21	3.5	0.170
C_CH2	CT	33.21	3.5	-0.120
C_CH3	CT	33.21	3.5	-0.180
C_CH3t	CT	33.21	3.5	-0.065
C_CHOH	CT	33.21	3.5	0.205
C_CH2OH	CT	33.21	3.5	-0.120
C_COH	CT	33.21	3.5	-0.265
C_PAM	CT	33.21	3.5	0.120
O_DET	OS	70.45	2.90	-0.300
O_ANI	OS	70.45	2.90	-0.285
O_KET	O_2	105.67	2.96	-0.470
O_FEN	OH	85.54	3.07	-0.585
O_OH	OH	85.54	3.12	-0.683
H_ARO	HA	15.09	2.42	0.115
H_FEN	HO	0.0	0.0	0.435
H_ET	HC	15.09	2.5	0.030
H_C	HC	15.09	2.5	0.060
H_OH	HO	0.0	0.0	0.418
H_CPA	HC	7.548	2.5	0.060
H_NPA	H	0.0	0.0	0.36
N_PAM	NT	85.54	3.3	-0.9

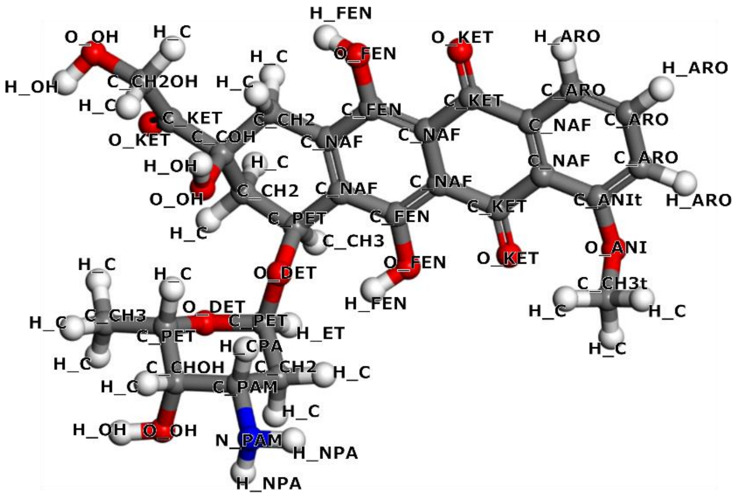

Figure S17: Indexing of the atoms in the doxorubicin model.

Table S7: Bonds, bends and torsion definitions for doxorubicin.

**Harmonic bond[1]**	**k_{eq} / K Å^{-2}**	**r_{eq} / Å**		
CT CT	269724.87	1.529		
CT HC	342188.27	1.090		
CT OH	322059.55	1.410		
OH HO	556558.68	0.945		
CT OS	322059.55	1.41		
CT C_2	319040.24	1.522		
CT NT	384458.59	1.448		
NT H	436793.27	1.010		
C_2 O_2	573668.57	1.229		
**Harmonic bend[2]**	**k_{eq} / K rad^{-2}**	**ϑ_{eq} / °**		
CT CT CT	58725.57	112.7		
CT CT HC	37741.35	110.7		
HC CT HC	33212.26	107.8		
CA CT HC	35225.26	109.5		
CT CT OH	50321.80	109.5		
CT OH HO	55353.99	108.5		
HC CT OH	35225.26	109.5		
CA OS CT	75482.71	111.0		
CT CT OS	50321.80	109.5		
HC CT OS	35225.26	109.5		
CT CT C_2	63405.47	110.10		
OH CT C_2	50321.80	109.5		
CT C_2 CT	70450.53	116.0		
CT C_2 O_2	80514.89	120.40		
HC CT C_2	35225.26	109.5		
CT CT NT	56561.64	109.47		
HC CT NT	35225.26	109.5		
CT NT H	35225.26	109.5		
H NT H	43880.64	106.4		

Torsion[3]	K_0 / kcal mol^{-1}	K_1 / kcal mol^{-1}	K_2 / kcal mol^{-1}	K_3 / kcal mol^{-1}
CT CT CT CT	1.299	-0.500	0.200	0.0
CT CT CT HC	0.0	0.0	0.300	0.0
HC CT CT HC	0.0	0.0	0.300	0.0
CT CT CT OH	1.710	-0.500	0.663	0.0
HC CT CT OH	0.0	0.0	0.468	0.0
CT CT OH HO	-0.356	-0.174	0.492	0.0
HC CT OH HO	0.0	0.0	0.450	0.0
CT CT OS CT	0.650	-0.250	0.670	0.0
HC CT OS CT	0.0	0.0	0.759	0.0
HC CT CT OS	0.0	0.0	0.468	0.0
OH CT CT OS	4.316	0.0	0.0	0.0
CA CA OS CT	0.0	2.998	0.0	0.0
CA OS CT HC	0.0	0.0	0.759	0.0
CA CA CT HC	0.0	0.0	0.0	0.0
CA CT CT HC	0.0	0.0	0.462	0.0
C_2 CT CT HC	0.0	0.0	-0.076	0.0
CT C_2 CT CT	1.453	-0.144	-0.774	0.0
CT C_2 CT HC	0.0	0.0	0.275	0.0
C_2 CT OH HO	-0.899	0.0	0.0	0.0
CT C_2 CT OH	1.710	-0.500	0.663	0.0
CT CT C_2 O_2	-0.277	1.227	-0.694	0.0
HC CT C_2 O_2	0.0	0.0	0.0	0.0
OH CT C_2 O_2	0.500	0.0	0.0	0.0
NT CT CT HC	-1.012	-0.708	0.468	0.0
CT CT NT H	-0.190	-0.417	0.418	0.0
HC CT NT H	0.0	0.0	0.400	0.0
NT CT CT OH	7.994	0.0	0.0	0.0

(1) Harmonic bond: $U = \frac{1}{2}k_{eq}\left(r_{ij} - r_{eq}\right)^2$

(2) Harmonic bend: $U = \frac{1}{2}k_{eq}\left(\theta_{ijk} - \theta_{eq}\right)^2$

(3) Torsion: $U = \frac{1}{2}K_0 + \frac{1}{2}K_1\left[1 + \cos\left(\varphi_{ijkl}\right)\right] + \frac{1}{2}K_2\left[1 - \cos\left(2\varphi_{ijkl}\right)\right] + \frac{1}{2}K_3\left[1 + \cos\left(3\varphi_{ijkl}\right)\right]$

The GCMC simulations carried out at 310 K and up to 150,000 Pa show zero uptake in UiO-66. **Figure S18** shows two snapshots of doxorubicin in UiO-66. These snapshots show that doxorubicin hardly fits inside the porosity of UiO-66, thus explaining the zero uptake.

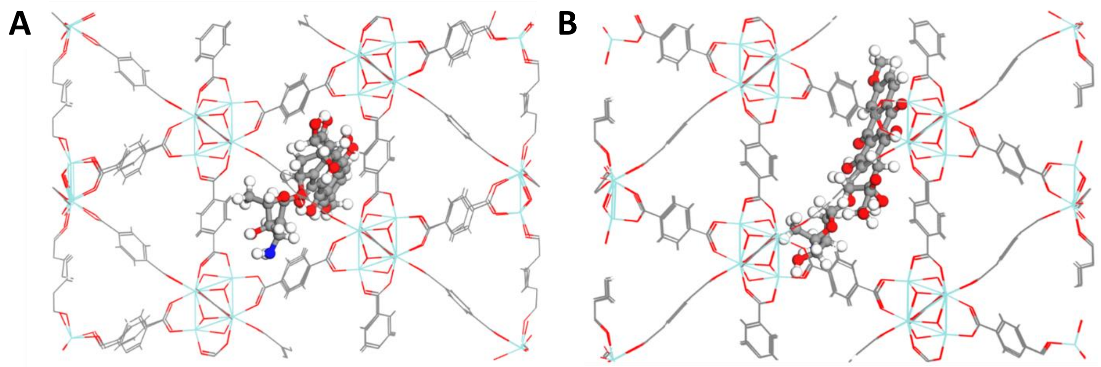

Figure S18: Snapshots of doxorubicin in UiO-66 from two perspectives.

The GCMC simulations on MIL-101(Cr) **(Figure S19)** lead to a saturation uptake of 2.1 mol kg^{-1} (or 1160 g kg^{-1}), which is ten times the amount adsorbed experimentally (110 g kg^{-1}). According to our calculation, the molecules adsorbed in the structure occupy 55.8% (1.046 cm^3 g^{-1}) of the pore volume (1.874 cm^3 g^{-1}). Therefore, given that the experimental adsorption is 9.5% of the maximum theoretical adsorption, this could occupy around 5.3% of the pore volume.

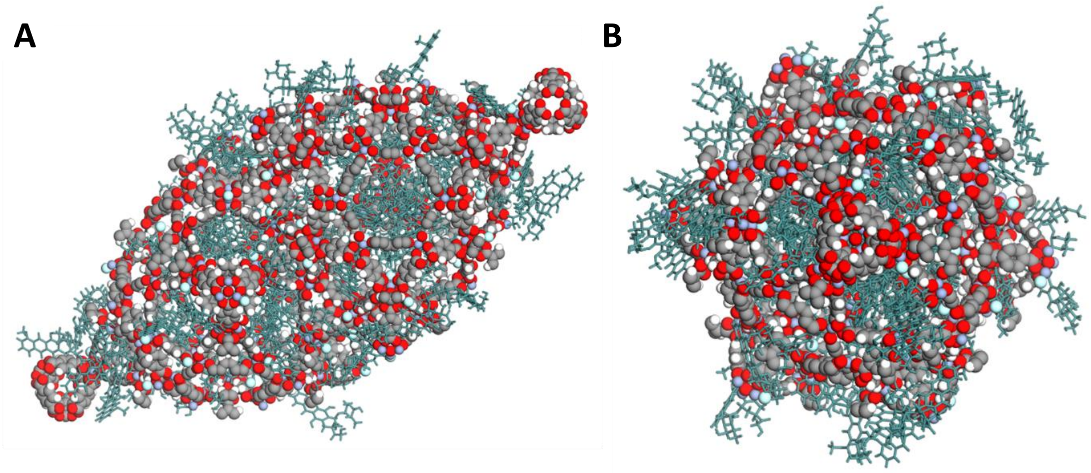

Figure S19: Snapshots of doxorubicin in the primitive cell of MIL-101(Cr) from two perspectives.

In these snapshots, the free volume within the structure seems completely full, which might be unexpected given that the 55.8% of the pore volume is occupied. However, the rigidity of the molecules and the difficulty to pack within the pores of the channel means some space is left between molecules, which explains the value of occupied pore volume.

For the drug delivery evaluation experiments the highest non-toxic concentration of each MOF was used. These were 1 μgmL^{-1} and 10 μgmL^{-1} for UiO-66 and MIL-101(Cr) respectively. In the case of the drug loaded materials (Dox@MOF), a normalisation of the weight to those previous concentrations was performed by taking into consideration their corresponding drug loading capacities, using equation **S3**.

$$\text{Dox@MOF}(\mu gmL^{-1}) = \frac{100 \text{ x } (\text{MOF } \mu gmL^{-1})}{(\text{MOF\% wt in Dox@MOF})} = \frac{100 \text{ x } (\text{MOF } \mu gmL^{-1})}{(100\text{-DLC\% wt})} \quad \textbf{(S3)}$$

This yielded concentrations of 2.38 μgmL^{-1} Dox@UiO-66 and 11.1 μgmL^{-1} Dox@MIL-101(Cr) being used. In a similar manner, the concentrations of equivalent amounts of free doxorubicin were calculated to be 1.38 μgmL^{-1} for Dox@UiO-66 and 1.1 μgmL^{-1} for Dox@MIL-101(Cr). A summary of the concentrations used for each material can be found on **Table S8**.

Table S8: Summary of concentrations used for each material for drug delivery experiments.

MOF	DLC (% wt)	MOF concentration (μgmL^{-1})	Dox@MOF concentration (μgmL^{-1})	Equivalent Dox concentration (μgmL^{-1})
UiO-66	58	1	2.38	1.38
MIL-101(Cr)	10	10	11.1	1.1

Drug release control: Fluorescence-based drug release experiments in cell culture media were carried out as a control. This was done to establish that any effect observed from the following experiments in this study corresponds to internalisation of the drug loaded nanoparticles and not to doxorubicin being released extracellularly and then being internalised by the cells.

A suspension of 100 μgmL^{-1} of DDS nanoparticles in complete Dulbecco's Modified Eagle cell culture Medium (DMEM) without phenol red was prepared with sonication for 15 minutes. A solution of an equivalent amount of free doxorubicin was also prepared for each system. The concentrations of doxorubicin used were 58 μgmL^{-1} for Dox@UiO-66 and 10 μgmL^{-1} for Dox@MIL-101(Cr). The fluorescence of the free drug was measured to establish the maximum fluorescence that could potentially be obtained from each DDS sample. Then, the fluorescence of the DDS suspension was measured over the time course of 8-9 days. In both cases there was a minimal initial burst release of about 20% and 10% of the drug for Dox@UiO-66 and Dox@MIL-101(Cr) respectively. This was to be expected as these are bare nanoparticles with no surface functionalisation adding protection against burst drug release. However, for both DDSs even after 8 days the drug release does not surpass 32% for Dox@UiO-66 and 20% for Dox@MIL-101(Cr) **(Figure S20)**. This like due to a combination of the stability of the MOF / drug coating or proteins present in the cell culture media (mainly albumin) forming a protective corona around the nanoparticles, inhibiting further drug release.[6-8]

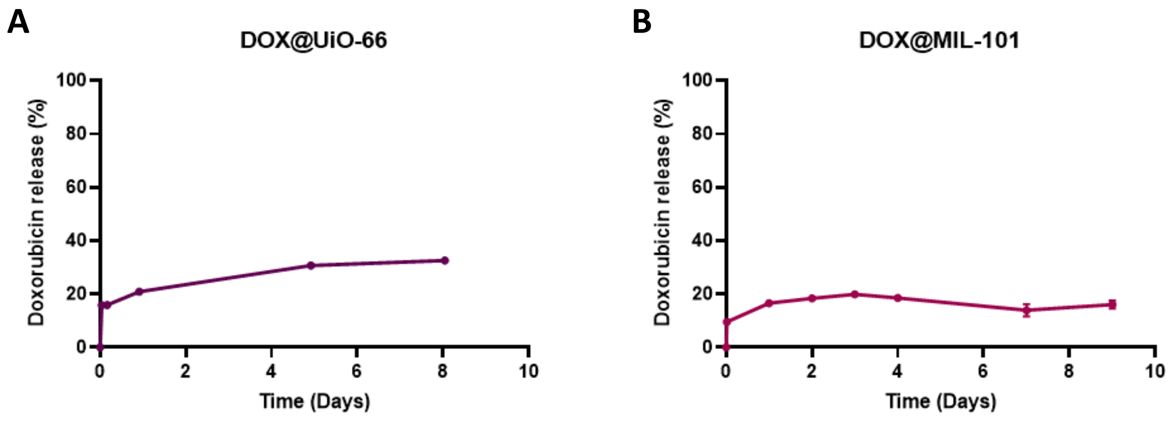

Figure S20: Fluorescence based doxorubicin release from (A) Dox@UiO-66 and (B) Dox@MIL-101(Cr) in complete cell culture media without phenol red. Data represented as mean ± SD (n=3).

Monitoring Drug Delivery by RTCA and Confocal Microscopy

RTCA: 100 µL per well complete cell culture media were added to the E-plate VIEW 96 and it was incubated at room temperature for 10 minutes. The plate was added to the RTCA instrument in the incubator (37 ˚C, 5% CO_2), to measure background impedance. The cells were counted and added to the plate at a concentration of 5×10^3 cells per well to a total volume of 200 µL per well. The plate was returned to the RTCA instrument and the cells were allowed to sediment for 30 minutes before the start of the measurement. After 24 hours of incubation, the media were carefully aspirated. The MOF and Dox@MOF nanoparticles were suspended in complete media and were sonicated for 10 minutes in the appropriate concentrations. Free doxorubicin was dissolved in complete cell culture media at the appropriate concentration and was sonicated until complete dissolution. The MOF suspension or free Dox treatment was added (200 µL per well) - 6 technical replicates for each concentration. Complete media were added in the untreated controls (200 µL per well) - 6 technical replicates. The plate was returned to the RTCA instrument where cell growth was recorded for a further 72 hours. Overall, 3 biological replicates were performed.

Confocal microscopy: MCF-7 cells were seeded in a Nunc™ Lab-Tec™ II CC2™ Chamber Slide with removable walls system at a density of $2x10^5$ cells per well (400 µL media per well). The cells were incubated for 24 hours in a humidified incubator atmosphere maintained at 37 ˚C and 5% CO_2. The Dox@UiO-66 and Dox@MIL-101(Cr) nanoparticles were suspended in complete media and were sonicated for 10 minutes in the appropriate concentrations. Free doxorubicin was dissolved in complete cell culture media at the appropriate concentration and was sonicated until complete dissolution. The media were removed from the wells by turning the slide over a beaker containing Virkon disinfectant. Complete cell culture media were added to the controls (400 µL per well) and the Dox@MOF suspension or free drug solution treatment was added to the cells (400 µL per well) - 2 technical replicates. The cells were incubated in a humidified incubator atmosphere maintained at 37 ˚C and 5% CO_2 for the appropriate time (4, 6 and 12 hours for Dox@UiO-66 and 4 and 8 hours for Dox@MIL-101(Cr)). The media were removed from the wells by turning the slide over a beaker containing Virkon disinfectant. The cells were washed twice with PBS 1X with $CaCl_2$ and $MgCl_2$ (400 µL per well). Then, 400 µL per well CellMask™ Green plasma membrane stain diluted 1:500 in warm media were added to all the samples apart from the nucleus stain controls where 400 µL of complete media were added. The cells were incubated for 30 min in a humidified incubator atmosphere maintained at 37 ˚C and 5% CO_2. The stain was removed by flipping over Virkon disinfectant and the cells were washed twice with PBS 1X with $CaCl_2$ and $MgCl_2$ (400 µL per well). Subsequentially, 100% methanol was added to the wells (400 µL per well) and the cells were incubated at room temperature for 5 minutes. The methanol was removed by flipping over Virkon disinfectant and the cells were washed twice with PBS 1X with $CaCl_2$ and $MgCl_2$ (400 µL per well). After the final wash, the wall framework of the slide was removed and one drop of ProLong™ Glass Antifade Mountant with NucBlue™ nuclei stain was added over each sample and the slide was covered with a thin glass slide. The slide was let dry in the dark at room temperature overnight and then was stored at 4 ˚C. Overall, 3 biological replicates were performed.

The cells were imaged using a ZEISS LSM 780 Confocal Microscope and ZEN Black software. The NucBlue™ nuclear stain was observed using laser line 405, emitting at 410-504 nm. The CellMask™ Green membrane stain was observed using laser line 514, emitting at 517-553 nm. Doxorubicin was observed using laser line 488, emitting at 569-627 nm. All images were captured using a 20X Plan Apochromat objective and the gain was set at 700. During image analysis, doxorubicin fluorescence intensity was quantified by employing the macro Batch_quantify_v3392[S2] on Fiji. To quantify doxorubicin fluorescence intensity within the cells, the CellMask™ Green stain was used as

colocalization mask, while the NucBlue stain was used as a colocalization mask to quantify doxorubicin within the cell nucleus.

Supplemental References

S1. Macrae, C.F., et al. (2020). Mercury 4.0: from visualization to analysis, design and prediction. J. Appl. Cryst., *53*, 226-235.

S2. Johansson, J., et al. (2019). RAL GTPases Drive Intestinal Stem Cell Function and Regeneration through Internalization of WNT Signalosomes. Cell Stem cell, *24*, 592-607.e7.

S3. Cavka, J.H., Jakobsen, S., Olsbye, U., Guillou, N., Lamberti, C., Bordiga, S., and Lillerud, K.P. (2008). A New Zirconium Inorganic Building Brick Forming Metal Organic Frameworks with Exceptional Stability. J. Am. Chem. Soc., *130*, 13850-13851.

S4. Férey, G., Mellot-Draznieks, C., Serre, C., Millange, F., Dutour, J., Surblé, S., and Margiolaki, I. (2005). A Chromium Terephthalate-Based Solid with Unusually Large Pore Volumes and Surface Area. Science, *309*, 2040.

S5. Valenzano, L., Civalleri, B., Chavan, S., Bordiga, S., Nilsen, M.H., Jakobsen, S., Lillerud, K.P., and Lamberti, C. (2011). Disclosing the Complex Structure of UiO-66 Metal Organic Framework: A Synergic Combination of Experiment and Theory. Chem. Mater., *23*, 1700-1718.

S6. Nazarenus, M., et al. (2014). In vitro interaction of colloidal nanoparticles with mammalian cells: What have we learned thus far? Beilstein J. Nanotechnol., *5*, 1477-1490.

S7. del Pino, P., Pelaz, B., Zhang, Q., Maffre, P., Nienhaus, G.U., and Parak, W.J. (2014). Protein corona formation around nanoparticles – from the past to the future. Mater. Horiz., *1*, 301-313.

S8. Rojas, S., Carmona, F.J., Maldonado, C.R., Horcajada, P., Hidalgo, T., Serre, C., Navarro, J.A.R., and Barea, E. (2016). Nanoscaled Zinc Pyrazolate Metal–Organic Frameworks as Drug-Delivery Systems. Inorg. Chem., *55*, 2650-2663.

S9. Dubbeldam, D., Calero, S., Ellis, D.E., and Snurr, R.Q. (2016). RASPA: molecular simulation software for adsorption and diffusion in flexible nanoporous materials. Mol. Simulat., *42*, 81-101.

S10. Rappe, A.K., Casewit, C.J., Colwell, K.S., Goddard, W.A., and Skiff, W.M. (1992). UFF, a full periodic table force field for molecular mechanics and molecular dynamics simulations. J. Am. Chem. Soc., *114*, 10024-10035.

S11. Mayo, S.L., Olafson, B.D., and Goddard, W.A. (1990). DREIDING: a generic force field for molecular simulations. J. Phys. Chem., *94*, 8897-8909.

S12. Jorgensen, W.L., Maxwell, D.S., and Tirado-Rives, J. (1996). Development and Testing of the OPLS All-Atom Force Field on Conformational Energetics and Properties of Organic Liquids. J. Am. Chem. Soc., *118*, 11225-11236.

S13. Rappe, A.K. and Goddard, W.A. (1991). Charge equilibration for molecular dynamics simulations. J. Phys. Chem., *95*, 3358-3363.
